# Intrapleural Administration With Rh-Endostatin and Chemical Irritants in the Control of Malignant Pleural Effusion: A Systematic Review and Meta-Analysis

**DOI:** 10.3389/fonc.2021.649999

**Published:** 2021-08-03

**Authors:** Cheng-Qiong Wang, Xiao-Rong Huang, Min He, Xiao-Tian Zheng, Hong Jiang, Qian Chen, Teng-Yan Fan, Lin Zhan, Juan Ling, Ji-Hong Feng, Xue Xiao, Xiao-Fan Chen, Zheng Xiao

**Affiliations:** ^1^Department of General Practice, Affiliated Hospital of Zunyi Medical University, Zunyi, China; ^2^Evidence-Based Medicine Center, MOE Virtual Research Center of Evidence-based Medicine at Zunyi Medical College, Affiliated Hospital of Zunyi Medical University, Zunyi, China; ^3^GCP Center, Affiliated Hospital of Zunyi Medical University, Zunyi, China; ^4^Department of Nursing, Affiliated Hospital of Zunyi Medical University, Zunyi, China; ^5^Evidence-Based Medicine Research Centre, Jiangxi University of Traditional Chinese Medicine, Nanchang, China; ^6^Laboratory Research Center, Guizhou Provincial People’s Hospital, Guizhou University, Guiyang, China; ^7^Department of Infection Management, Gansu Provincial People’s Hospital, Lanzhou, China; ^8^Department of Oncology, Lishui People’s Hospital, Sixth Affiliated Hospital of Wenzhou Medical University, Lishui, China

**Keywords:** endostatin, recombinant human endostatin (Rh-endostatin), chemical irritants, cisplatin, optimal adjuvant strategy, meta-analysis

## Abstract

**Introduction:**

A modified and recombinant human endostatin (Rh-endostatin) is often used in the control of malignant pleural effusion (MPE) through intrapleural infusion.

**Objectives:**

To demonstrate the clinical response, survival, and safety of Rh-endostatin plus chemical irritants, their optimal combinations, treatment threshold, and optimal usage, we performed a new systematic review and meta-analysis.

**Methodology:**

All randomized controlled trials (RCTs) were collected from Chinese and English electronic databases (from inception until August 2020). We pooled the data using a series of meta-analyses and summarized the evidence quality following the Grading of Recommendations Assessment, Development and Evaluation (GRADE) approach.

**Results:**

We included 75 RCTs recruiting 4,678 patients, which reported six combinations for Rh-endostatin plus chemical irritants. Among the six combinations, only Rh-endostatin plus cisplatin (DDP) with enough trials might improve the complete response [2.29 (1.93, 2.71)] and quality of life [3.01 (2.49, 3.63)] and reduce treatment failure [0.29 (0.25, 0.33)] and progressive disease [0.27 (0.22, 0.34)]. It might not increase the risk of adverse drug reactions. For patients with lung cancer, moderate to massive effusion, initial treatment, Karnofsky Performance Status (KPS) score ≥60, or anticipated survival time ≥3 months, Rh-endostatin (30–45 mg each time, once or twice a week 3–4 times) plus DDP (30–60 mg/m^2^) obtained a significant improvement in clinical response and a reduction of failure and progressive disease. Most results had good robustness and moderate quality.

**Conclusions:**

Current evidence suggests that Rh-endostatin with DDP may be an optimal combination, which may improve clinical response and reduce failure and progressive disease with good safety. Rh-endostatin (30–40 mg each time, once or twice a week 3–4 times) with DDP (30–40 mg/m^2^) may be an optimal usage for achieving an ideal response.

## Introduction

Malignant pleural effusion (MPE) is a common clinical problem in patients with malignant tumors, with an estimated annual incidence of at least 150,000 in the USA (1). Based on postmortem records, MPE was found in 15% of patients who died with malignant tumors (2). Most patients often suffered from breathlessness and chest pain. The quality of life (QOL) was poor, and the median survival time was only 3–12 months (2, 3). Chemical pleurodesis is a first-line treatment for symptomatic patients with MPE and suspected expandable lung (4, 5) and a procedure performed to obliterate the pleural space to prevent recurrent MPE using a chemical irritant as platinum, bleomycin (BLM), tetracycline, doxycycline, or silver nitrate, among others (3–6). However, these strategies are mostly of palliative value and focus on the control of symptoms and improvement of QOL and fail to improve survivals. So, new control strategies are urgently needed.

Proangiogenic factors have been implicated as a critical cytokine in the occurrence, development, and transferring of MPE (7–10). Endostatin, a 20-kDa C-terminal fragment of type XVIII collagen, is one of the most potent inhibitors of angiogenesis (11). Endostatin and its derivatives have been reported to be more effective when combined with chemotherapy, radiotherapy, or gene transfer in the treatment of malignant tumors (12, 13). Endostar, a modified and recombinant human endostatin (Rh-endostatin), was the approved regimen in non-small-cell lung cancer (NSCLC) by the State Food and Drug Administration of China in 2005 (14). The expert consensus also recommends Rh-endostatin plus first-line chemotherapy to treat stage III/IV NSCLC (15, 16). Interestingly, eight systematic reviews (SRs)/meta-analyses had reported that intrapleural administration of Rh-endostatin with platinum (17, 18), cisplatin (DDP) (19–23), or chemotherapeutic agents (24) might improve the objective response rate [complete response (CR), partial response (PR)], disease control rate [CR + PR+ no response (NR)/stable disease (SD)], and QOL, without an increase in the incidence of adverse drug reactions (ADRs) in MPE. Three meta-analyses (25–27) had reported that Rh-endostatin with DDP also might obtain the same effects in MPE from lung cancer. Based on the above evidence, Rh-endostatin alone or plus chemical irritants was recommended in the control of MPE by expert consensus from China (28). However, strong clinical heterogeneity was found in the patient features, types, combinations, and usages of Rh-endostatin/chemical irritants. The drug usages are complex, diverse, and even inappropriate. Obviously, the current studies ignored clinical heterogeneity. Current evidence (17–27) failed to conclusively demonstrate whether Rh-endostatin plus chemical irritants improves clinical response, survival, and safety. Their optimal combinations, therapeutic threshold, and optimal usage remain unclear. In addition, no evidence revealed their thoracentesis-related adverse events (TRAEs). All these have become the new bottleneck of rational drug use decision.

Recently, many new trials (29–31) have been published. So, we performed a new SR and meta-analysis to further demonstrate the clinical response, survival, and safety of Rh-endostatin with chemical irritants, reveal their optimal combinations, therapeutic thresholds, and optimal usage for achieving a desired response, and provide evidence for developing an optimal control strategy of MPE.

## Methods

According to the principle of underestimating efficacy and overestimating risk, we designed, implemented, and reported this SR and meta-analysis following the Preferred Reporting Items for Systematic reviews and Meta-Analyses (PRISMA) guidelines **(** **Supplementary Material S1** **)** (32). The retrieval, selection, assessment, data collection, statistical analysis, and summary of evidence quality were implemented by two independent evaluators. Any disagreements of implementations between evaluators were resolved by discussions, and further disagreements were resolved by a third party (ZX).

### Inclusion Criteria

All subjects were patients with MPE that was diagnosed using thorax imaging, pleural fluid analysis, cytology, or pleural biopsy, without any restrictions on the tumor types. All subjects had normal heart, liver, or kidney function. The intervention used was Rh-endostatin through intrapleural administration instead of intravenous injection. Patients in the experimental group received Rh-endostatin plus chemical irritant, and the control group received chemical irritant alone, which included platinum, BLM, tetracycline, doxycycline, or silver nitrate, among others. During perfusion, all subjects did not receive hyperthermia, radiotherapy, chemotherapy, chemoradiotherapy, traditional Chinese medicine injections (TCMIs), or other biological response modifiers (BRMs). The main outcomes were clinical responses, survivals, and QOL, and the secondary outcomes were ADRs and TRAEs. The trials were randomized controlled trials (RCTs), with no restrictions on follow-up and research institutions.

### Exclusion Criteria

Excluded studies included the duplicates; studies about non-MPE and non-Rh-endostatin; studies about Rh-endostatin plus hyperthermia, radiotherapy, chemotherapy, chemoradiotherapy, TCMIs, or BRMs; meeting abstracts and reviews without any specific data; non-RCTs as cohort studies, cross-sectional studies, case series, or case reports; unrelated SRs or meta-analyses; and studies without primary or secondary outcome data.

### Search Strategies

Based on the principle of patients (P) plus intervention (I), we applied the MeSH and free word to build the search strategies as (“Pleural Effusion” [Mesh]**** OR Pleural Effusion OR Pleural Effusions OR Hydrothorax OR MPEs OR MPE) AND (“Endostatins”**** [Mesh] OR Endostatins OR Endostatin OR Recombinant human endostatin injection OR rhES OR Rh-endostatin OR Endostar OR Sulijia OR YH-16). Two independent evaluators (C-QW and HJ) collected all the published studies of “Rh-endostatin plus chemical irritants for MPE” from Embase, PubMed, Web of Science (ISI), China Biological Medicine Database (CBM), Wanfang Database, China National Knowledge Infrastructure Database (CNKI), Chinese Scientific Journals Full-text Database (VIP), and Cochrane Central Register of Controlled Trials (CENTRAL, Issue 8 of 12, August 2020) and ongoing trials from the Chinese clinical trial registry (Chi-CTR, http://www.chictr.org.cn), WHO International Clinical Trials Registry Platform (WHO-ICTRP, http://apps.who.int/trialsearch/), and US-clinical trials (https://clinicaltrials.gov/, up to August 2020). In addition, we critically evaluated all the SRs/meta-analyses of Rh-endostatin in MPE and selected eligible trials from the references.

### Selection of Studies

Two evaluators (C-QW and MH) were asked to collect the qualified trials about Rh-endostatin plus chemical irritants for MPE according to the preestablished inclusion and exclusion criteria.

### Assessment of Methodological Bias Risk

Two evaluators (X-RH and QC) were asked to assess the bias risk of methodology using the Cochrane Collaboration’s risk of bias assessment tool for RCTs (33). The bias risk was assessed as a judgment (high, low, or unclear) for individual elements of five domains (selection, performance, attrition, reporting, and other).

### Indicator Definition

The clinical responses were evaluated using CR, treatment failure, and progressive disease (PD). Based on previous studies (34–37), we integrated all the criteria as follows: (i) CR, (ii) PR, (iii) NR or SD; and (iv) PD **(** **Supplementary Material S2** **)**. Treatment failure was defined as NR/SD plus PD (38). Survival was defined as overall survival (OS) rate, progression-free survival (PFS) rate, or hazard ratio (HR) of the OS and PFS. Using the Karnofsky Performance Status (KPS) scale, if the KPS score increased ≥10 after perfusion, the QOL was improved.

The secondary outcomes were ADRs and TRAEs. According to the World Health Organization (WHO) (39) or Common Terminology Criteria for Adverse Events (CTCAE) standards (40), ADR was defined as neutropenia, thrombocytopenia, anemia, cardiotoxicity, hepatotoxicity, nephrotoxicity, gastrointestinal reactions, alopecia, peripheral neuritis, chest pain, and fever, among others. TRAE was defined as treatment-related mortality (TRM) and a series of clinical symptoms such as respiratory failure, pneumothorax, cutaneous emphysema, or catheter-related infection/chest infection, among others.

### Data Collection

Two evaluators (X-TZ and T-yF****) collected all the data using a predesigned data extraction form. The data included the first author, year of publication, and demographic information of patients; baseline characteristics such as primary tumors, pleural fluid volume, KPS score, treatment history (initial treatment, retreatment, or both), anticipated survival time (AST), sample size, drainage methods [indwelling pleural catheters (IPCs) or thoracocentesis]; combinations and usages of Rh-endostatin and chemical irritants; evaluation time and follow-up protocols; and outcomes including CR, treatment failure, PD, OS, PFS, QOL, ADRs, and TRAEs. Additionally, we contacted the corresponding author to obtain the available survival data. If the authors were unavailable, we adopted the Engauge Digitizer 4.1 to transform the Kaplan–Meier survival curves into available data (41, 42).

### Statistical Analysis

According to the data features, the odds ratio (OR) or hazard ratio (HR) and their 95% CI were used to quantify the CR, treatment failure, PD, OS, PFS, QOL, ADRs, and TRAEs, and p < 0.05 was considered a statistical significance. Two evaluators (C-QW and X-RH) conducted a series of meta-analyses using the Review Manager 5.4.1 (as recommended by the Cochrane Collaboration). The Cochran’s χ^2^ test and I^2^ statistic were conducted to analyze the potential statistical heterogeneity. If p ≥ 0.1 and I^2^ ≤ 50%, a fixed-effects model (FEM) was used to pool the OR or HR and their 95% CI. Otherwise, a random-effects model (REM) was used. If the number of trials was larger than 10, a funnel plot and Egger/Begg’s test were used to examine the potential publication bias.

When at least one item was considered a high risk, the trial was defined as poor quality. When the result was statistically different and beneficial to Rh-endostatin infusion, the trial was defined as an underestimated or overestimated trial following our experiences (38, 43, 44). According to the principle of underestimating efficacy and overestimating risk, we established a sensitivity analysis model to analyze the robustness of the results before and after eliminating the trials with poor quality, underestimation, or overestimation.

### Subgroup Analysis

Following the guideline (45) and our previous experiences (38, 43, 44), we established a subgroup analysis model to analyze the clinical heterogeneity and the effects of variables on CR, treatment failure, and PD and to reveal their treatment thresholds and optimal usage for achieving an ideal response. The variables included patient features, drainage methods, and combinations of Rh-endostatin plus chemical irritants and their dose, treatment frequency, and times. Finally, a univariable random-effects meta-regression was conducted to reveal the relevance between each variable and CR, treatment failure, or PD and a post-hoc multiple regression analysis adjusting for their OR under all variables.

### Summary of Evidence Quality

Following the Grading of Recommendations Assessment, Development and Evaluation (GRADE) approach and integrating the results of the sensitivity analysis, we developed a quality summary model to summarize the evidence quality and classify them as “high,” “moderate,” “low,” or “very low” (38, 43, 44) **(** **Supplementary Material S3** **)**. The quality was downgraded according to five domains as follows: (1) methodological bias risk; (2) statistical heterogeneity; (3) indirectness; (4) imprecision; and (5) publication bias. Two evaluators (X-FC and C-QW) used the GRADE profiler to summarize the quality and generate the absolute estimates for the CR, treatment failure, PD, OS rate, PFS rate, QOL, ADRs, and TRAEs (46).

## Results

### Search Results

**Figure 1:**
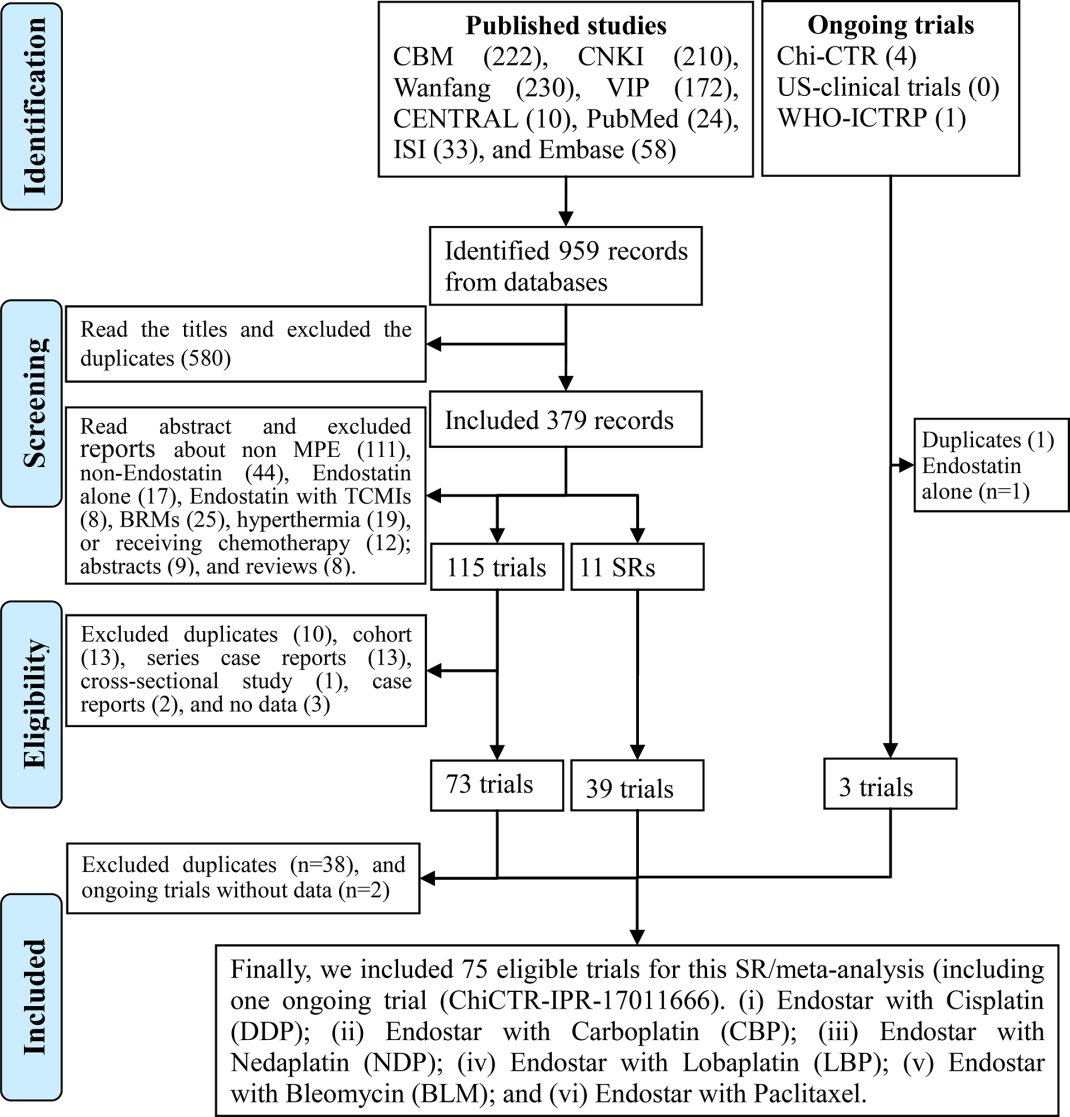
Preferred Reporting Items for Systematic reviews and Meta-Analyses (PRISMA) 2009 flow diagram for the identification of eligible trials.

A literature search conducted from inception to August 17, 2020, identified 959 studies. After duplicates were removed, 379 studies remained for a review of abstracts. After reviewing the abstracts, we identified 115 reports and 11 SRs/meta-analyses (16, 47–54). After reviewing full texts, we identified 73 qualified trials (29–31, 54–123). After reviewing the SRs/meta-analyses, we identified 39 trials (55–60, 62, 64, 66–69, 71–78, 81–92, 95, 97, 100, 101, 104, 109, 110). Excluding two ongoing trials without data (124, 125), we included one ongoing trial (ChiCTR-IPR-17011666) (126). Finally, we identified 75 trials (29–31, 54–123, 126, 127) for this SR/meta-analysis **(** **Supplementary Material S4** **;** **Tables S1–S4** **;** **Figure 1** **).**

### Characteristics of the Included Trials

The 75 trials, published from 2010 to 2020, recruited 4,678 patients with MPE from China, which included 2,512 males and 1,738 females aged 18–89 years **(** **Table 1** **)**. Forty-seven trials recruited patients with lung cancers (30, 31, 55, 58, 59, 64–67, 70, 74, 75, 77–84, 89–95, 97, 100–107, 109–111, 113–116, 118, 120, 121, 127), three trials recruited patients with breast cancer (99, 112, 117), and the remaining 25 trials (29, 54, 56, 57, 60–63, 68, 69, 71–73, 76, 85–88, 96, 98, 108, 119, 122, 123, 126) recruited patients with malignant tumors such as lung cancer, breast cancer, malignant lymphoma, gastric cancer, hepatic carcinoma, and ovarian cancer, among others. Trials were of varied sample sizes, from 30 to 130. The patients had small to massive effusion, KPS ≥40, and AST ≥2 months. Fifteen trials reported the treatment history as initial treatment, retreatment, or both. After the drainage of hydrothorax using an IPC or thoracocentesis, 2,352 cases accepted the intrapleural administration of Rh-endostatin plus chemical irritants, while 2,326 other cases accepted the chemical irritants alone. We found six combinations of Rh-endostatin plus DDP in 60 trials (30, 31, 54–56, 58, 60, 62, 64, 66–68, 71–73, 75–78, 80–82, 84–92, 94, 95, 97–101, 103, 104, 106–123, 126, 127), nedaplatin (NDP) (69, 74, 83, 102) and BLM in four trials (57, 61, 65, 70), lobaplatin (LBP) in three trials (29, 93, 105), carboplatin (CBP) in two trials (59, 96), paclitaxel (79) or DDP/BLM in one trial (63). Rh-endostatin (30–90 mg each time) was used once or twice a week, 1–12 times by intrapleural administration. The chemical irritant was mainly DDP and used with 20–100 mg/m^2^ each time. Three to 10 weeks after perfusion, the trials evaluated clinical responses using a Millar or Ostrowskimj criterion, ADRs using a WHO criterion, and QOL using a KPS scale. In addition, only nine trials reported the survivals (29–31, 63, 71, 75, 98, 105, 116) and TRAEs (56, 66, 76, 88, 95, 98, 99, 116, 127), and six trials (66, 88, 95, 98, 99, 116) reported the TRM.

**Table 1:** Characteristics of the included trials.

First author, Year	Malignant pleural effusions									Interventions		ET	Criteria A/B	O
	Tumor	Volume	TH	KPS	AST	IPC	E/C	M/F	Years	Rh-Endostatin	Chemical irritants			
**Rh-Endostatin with Cisplatin (DDP)**														
Huang, D.2010 (55)	NSCLC	Unclear	PT/RT	≥60	Un	Yes	18/18	20/16	27–65	45 mg/time, 1 time/w, 3 times	40 mg/m^2^	3 weeks	Millar, Unclear	O1-3
Li, J.2010 (56)	MTs	Moderate to large	Un	≥60	>3	Yes	33/34	Un	40–70	30 mg/time, 1 time/w, 3 times	40 mg/m^2^	3 weeks	Millar, NCICTC	O1-3
Li, W.2011 (58)	NSCLC	Un	PT/RT	≥60	Un	Yes	21/21	25/17	25–68	45 mg/time, 1 time/w, 3 times	60 mg/m^2^	3 weeks	Millar, Unclear	O1-3
Mao, L.2011 (60)	MTs	Large	PT	≥60	≥3	Yes	45/45	49/41	27–70	45 mg/time, 2 times/w, 4 times	40mg/m^2^	8 weeks	Millar, NCICTC	O1-3
Jiang, B.2012 (62)	MTs	Moderate to large	Un	>60	>3	Yes	30/30	37/23	59+11	30 mg/time, 2 times/w, 2 times	60 mg/m^2^	2 months	Millar, NCICTC	O1-3
Liu, X.2012 (64)	NSCLC	Moderate to large	Un	≥60	>3	Yes	30/30	37/23	52–68	60 mg/time, 2 times/w, Un	60 mg/m^2^	Un	Millar, WHO	O1-3
Miao, H.2012 (66)	LC	Moderate to large	Un	≥60	≥2	Yes	24/24	Un	29–70	45–60 mg/time, 1 time/w,3 times	40 mg/m^2^	3 weeks	Millar, WHO	O1-3
Shen, Q.2012 (67)	NSCLC	Moderate to large	PT	≥60	>3	Yes	40/40	42/38	37–79	30 mg/time, 2 times/w, 6 times	40 mg/m^2^	4 weeks	Millar, WHO	O1-3
Wang, X.2012 (68)	MTs	Moderate to large	Un	Un	Un	Yes	21/25	28/18	28–76	45 mg/time, 1 time/w, 2 times	50 mg/m^2^	2 months	Ostrowskimj, Un	O1,3
Han, Z.2013 (71)	MTs	Moderate to large	Un	≥60	>3	Yes	20/20	24/16	52–70	45 mg/time, 1 time/w, 1–3 times	20–60 mg/m^2^	4-7 weeks	Millar, NCICTC	O1-4
He, L.2013 (72)	MTs	Un	Un	>60	Un	Yes	32/32	40/24	37–75	30 mg/time, 2 times/w, 8 times	40 mg/m^2^	4 weeks	Millar, Un	O1-3
Kang, L.2013 (73)	MTs	Moderate to large	Un	Un	>3	Yes	30/30	39/21	36–75	45 mg/time, 3 times/w, 3 times	40 mg/m^2^	5 weeks	Millar, NCICTC	O1-3
Yang, Y.2013 (75)	NSCLC	Moderate to large	Un	Un	Un	Un	21/21	27/15	37–80	30 mg/time, 2 times/w, 6 times	40 mg/m^2^	3 w-2 years	Millar, Un	O1-4
Zheng, Q.2013 (76)	MTs	Un	Un	≥60	≥3	Yes	60/60	73/47	32–75	90 mg/time, 1 time/w, 3–12 times	30–40 mg/m^2^	12 weeks	Millar, NCICTC	O1-3
Chen, J.2014 (77)	NSCLC	Un	Un	Un	Un	Yes	30/30	44/16	46–66	45 mg/time, 2 times/w, 6 times	40 mg/m^2^	Un	Millar, Un	O1,3
Huang, L.2014 (78)	NSCLC	Moderate to large	Un	>60	Un	Yes	25/25	30/20	37–80	30 mg/time, 2 times/w, 4 times	50 mg/m^2^	4 weeks	Millar, Un	O1,3
Li, Y.2014 (80)	LC	Un	Un	>60	>6	Yes	42/42	46/38	62–84	60 mg/time, Un, Un	60 mg/m^2^	4 weeks	Millar, NCICTC	O1-3
Lu, H. 2014 (81)	LC	Moderate to large	Un	≥60	≥3	Yes	30/30	41/19	37–75	45 mg/time, 1 time/w, 4 times	100 mg/m^2^	4 weeks	Millar, WHO	O1,3
Tu, J.2014 (82)	NSCLC	Moderate to large	Un	≥60	≥3	Yes	45/45	48/42	45–70	45 mg/time, 2 times/w, 6 times	40 mg/m^2^	7 weeks	Millar, WHO	O1-3
Yue, G.2014 (84)	NSCLC	Large	PT	≥60	≥3	Un	43/43	47/39	38–69	30 mg/time, 2 times/w, 4–6 times	60 mg/m^2^	4 weeks	Ostrowskimj, Un	O1-3
Dong, M.2015 (85)	MTs	Small to large	Un	≥50	>3	Yes	23/23	25/21	Un	30 mg/time, 2 times/w, 4 times	60 mg/m^2^	6 weeks	Millar, Un	O1-3
Hu, X.2015 (86)	MTs	Moderate to large	PT/RT	≥60	≥3	Yes	43/41	62/22	28–76	60 mg/time, 2 times/w, 2–4 times	40–50mg/m^2^	Un	Millar, NCICTC	O1-3
Pang, Z.2015 (87)	MTs	Moderate to large	Un	≥60	≥3	Yes	21/25	30/16	40–75	45 mg/time, 3 times/w, 3 times	40 mg/m^2^	Un	Ostrowskimj, Un	O1,3
Zhao, W.2015 (88)	MTs	Moderate to large	Un	≥50	≥3	Yes	18/18	Un	Un	60 mg/time, 3 times/w, 6 times	60 mg/m^2^	3 weeks	Millar, NCICTC	O1-3
Chang, Y.2016 (89)	LC	Un	Un	Un	>2	Yes	26/26	33/19	38–77	90 mg/time, 2 times/w, 2 times	60 mg/m^2^	Un	Millar, Un	O1,3
Chen, F.2016 (90)	NSCLC	Moderate to large	Un	≥60	≥2	Yes	30/30	39/21	Un	45 mg/time, 1 time/w, 3 times	40 mg/m^2^	7 weeks	Millar, WHO	O1-3
Chen, R.2016 (91)	NSCLC	Moderate to large	Un	≥60	>3	Yes	45/45	53/37	44–76	45 mg/time, 2 times/w, 6 times	40mg/m^2^	3 weeks	Millar, Un	O1-3
He, J.2016 (92)	NSCLC	Moderate to large	Un	≥70	>3	Yes	27/25	32/20	54–74	30 mg/time, 2 times/w, 6 times	40 mg/m^2^	7 weeks	Millar, NCICTC	O1-3
Li, Y.2016 (94)	NSCLC	Un	Un	>60	Un	Un	31/31	35/27	36–80	30 mg/time, 2 times/w, 4 times	50 mg/m^2^	4 weeks	Millar, Un	O1,3
Lu, J.2016 (95)	LC	Moderate to large	PT	≥60	>3	Yes	30/30	28/32	Un	30 mg/time, 3 times/w, 3–6 times	30 mg/m^2^	4 weeks	Millar, NCICTC	O1-3
Qin, M.2016 (97)	NSCLC	Moderate to large	Un	≥60	Un	Yes	21/21	24/18	42–78	60 mg/time, 1 time/w, 3 times	50 mg/m^2^	7 weeks	Millar, NCICTC	O1,3
Song, X. 2016 (98)	MTs	Moderate to large	PT	≥70	≥3	Yes	19/17	20/16	31–78	45–60 mg/time, 2 times/w, 2 times	50 mg/m^2^	2 weeks	Millar, NCICTC	O1-4
Zhang, P.2016 (99)	BC	Small to large	Un	≥60	>3	Yes	26/25	0/51	31–64	45 mg/time, 3 times/w, 9 times	40 mg/m^2^	3 weeks	Millar, NCICTC	O1-3
Zheng, W.2016 (100)	NSCLC	Moderate to large	Un	≥60	>3	Yes	46/46	71/21	49–72	45 mg/time, 3 times/w, 3–6 times	40 mg/m^2^	1 week	Millar, NCICTC	O1-3
Zhou, J.2016 (101)	NSCLC	Un	Un	>60	>6	Yes	53/53	74/32	61–83	45 mg/time, Un, Un	60 mg/m^2^	1 week	Millar	O1
Zou, J.2016 (103)	LC	Un	Un	>60	>3	Yes	36/36	41/31	44–79	30 mg/time, 2 times/w, Un	50 mg/m^2^	Un	Millar, Un	O1,3
Che, X.2017 (104)	LC	Large	Un	≥50	>3	Yes	40/40	58/22	Un	90 mg/time, 1 time/w, 4 times	50 mg/m^2^	Un	Millar, Un	O1-3
ChiCTR.2017 (126)	MTs	Un	Un	≥40	>3	Un	29/24	35/18	Un	Un, Un, Un	Un	Un	Millar, Un	O1-3
Feng, Z. 2017 (106)	NSCLC	Moderate to large	Un	Un	Un	Yes	27/27	32/22	Un	30 mg/time, 1 time/w, 3 times	30 mg/m^2^	4 weeks	Millar,	O1
Gui, P.2017 (127)	NSCLC	Moderate to large	Un	Un	≥3	Yes	65/65	73/57	43–72	30 mg/time, 2 times/w, Un	50 mg/m^2^	4 weeks	Millar, Un	O1-3
Han, Z.2017 (107)	NSCLC	Large	Un	Un	Un	Yes	15/15	16/24	37–66	30 mg/time, 2 times/w, 1–3 times	20–40 mg/m^2^	1 weeks	Millar, NCICTC	O1,3
Jia, X.2017 (108)	MTs	Un	PT/RT	≥70	≥3	Yes	22/18	21/19	Un	45 mg/time, 2 times/w, 4 times	40 mg/m^2^	4 weeks	Millar, NCICTC	O1,3
Lu, X.2017 (109)	NSCLC	Moderate to large	Un	≥60	≥3	Yes	31/31	35/27	Un	45 mg/time, 2 times/w, 4 times	40 mg/m^2^	7 weeks	Millar, WHO	O1-3
Zhao, Q.2017 (110)	LC	Un	Un	Un	Un	Yes	34/34	37/31	46–76	45 mg/time, 1 time/w, 4 times	60 mg/m^2^	4 weeks	Ostrowskimj, Un	O1,3
Chen, X.2018 (111)	LC	Un	Un	Un	Un	Yes	50/50	58/42	28–75	45 mg/time, 1 time/w, 3 times	40 mg/m^2^	4 weeks	Millar, Un	O1-3
Fan, Y.2018 (112)	BC	Un	Un	≥60	≥3	Yes	45/45	Un	41–75	60 mg/time, 2 times/w, Un	60 mg/m^2^	Un	Millar, Un	O1-3
Li, T.2018 (113)	LC	Un	Un	Un	Un	Yes	30/30	41/19	50–80	45 mg/time, 2 times/w, 6 times	40 mg/m^2^	Un	Millar, Un	O1,3
Liu, H.2018 (114)	NSCLC	Moderate to large	PT	Un	≥3	Yes	26/26	23/29	39–75	45 mg/time, 2 times/w, 4–6 times	30 mg/m^2^	3–4 weeks	Millar, WHO	O1,3
Liu, Y.2018 (115)	NSCLC	Moderate to large	Un	>60	≥3	Yes	34/34	38/30	53–72	60 mg/time, 2 times/w, Un	60 mg/m^2^	Un	Millar, WHO	O1-3
Qing, S.2018 (116)	NSCLC	Moderate to large	Un	Un	≥2	Yes	28/23	22/29	49–77	35 mg/time, 2 times/w, Un	60 mg/m^2^	3 w-1 year	Millar, WHO	O1-4
Qiu, H.2018 (117)	BC	Moderate to large	Un	Un	Un	Yes	23/23	Un	Un	60 mg/time, 2 times/w, 8 times	60 mg/m^2^	4 weeks	Millar, Un	O1,3
Rao, X.2018 (54)	MTs	Moderate to large	PT	≥60	≥6	Yes	40/40	47/33	48–75	30 mg/time, 1 time/w, 3 times	60 mg/m^2^	3 weeks	Millar, Un	O1,3
Reng, D.2018 (118)	LC	Moderate to large	Un	≥60	>3	Yes	20/20	17/23	18–75	60 mg/time, 1 time/w, 6 times	40 mg/m^2^	8 weeks	Millar, WHO	O1-3
Song, W.2018 (119)	MTs	Moderate to large	Un	>60	>3	Un	30/30	43/17	Un	45 mg/time, 2 times/w, 12 times	60 mg/m^2^	12 weeks	Millar, WHO	O1-3
Wang, R. 2018 (120)	NSCLC	Un	Un	≥60	≥3	Yes	30/30	35/25	44–75	45 mg/time, 2 times/w, 6 times	40 mg/m^2^	3 weeks	Millar, WHO	O1,3
Jiang, W.2019 (121)	LC	Un	Un	Un	Un	Un	40/40	56/24	53–79	40 mg/time, 1 time/w, 4 times	40 mg/m^2^	4 weeks	Millar, WHO	O1,3
Tian, L.2019 (122)	MTs	Moderate to large	Un	>60	>2	Yes	48/48	57/39	50–70	40 mg/time, 4 times/w, Un	30–40 mg/m^2^	4 weeks	Millar	O1,3
Zheng, D.2019 (123)	MTs	Un	Un	Un	Un	Yes	24/24	25/23	26–75	30 mg/time, 2 times/w, 4 times	30 mg/m^2^	6 weeks	Ostrowskimj, Un	O1,3
Li, S.2020 (31)	NSCLC	Moderate to large	Un	Un	Un	Yes	20/20	24/16	43–71	45 mg/time, 1 time/w, 3 times	40 mg/m^2^	7 w-1 year	Millar, Un	O1-4
Xu, M.2020 (30)	NSCLC	Large	PT/RT	≥50	>2	Yes	20/20	27/13	Un	60 mg/time, 2 times/w, 4 times	40–50 mg/m^2^	5 w-1 year	Millar, NCICTC	O1-4
**Rh-endostatin with carboplatin (CBP)**														
Liu, Z.2011 (59)	LC	Moderate to large	PT	≥40	>3	Yes	23/23	26/20	26–79	45 mg/time, 1 time/w, 4 times	400 mg/m^2^	5 weeks	Millar, Unclear	O1,3
Pang, H.2016 (96)	MTs	Moderate to large	Un	>60	>3	Yes	33/30	31/32	Un	60 mg/time, 1 time/w, 2 times	400 mg/m^2^	4 weeks	Millar, NCICTC	O1,3
**Rh-Endostatin with Nedaplatin (NDP)**														
Yao, Q.2012 (69)	MTs	Moderate to large	Un	≥60	≥3	Yes	30/30	42/18	35–78	45 mg/time, 1 time/w, Un	40 mg/m^2^	Un	Millar, WHO	O1-3
Yang, K. 2013 (74)	LC	Moderate to large	PT	≥70	>3	Yes	28/28	38/20	44–65	7.5 mg/m^2^, 1 time/w, 6 times	100 mg/m^2^	10 weeks	Ostrowskimj, Un	O1,3
Xu, J.2014 (83)	NSCLC	Moderate to large	Un	Un	>3	Yes	35/35	43/27	44–70	60 mg/time, 1 time/w, 2 times	60 mg/m^2^	4 weeks	Millar, NCICTC	O1,3
Zhou, Y.2016 (102)	LC	Moderate to large	Un	≥60	≥3	Yes	24/24	Un	36–72	30 mg/time, 1 time/w, 3 times	60 mg/m^2^	3 weeks	Millar, NCICTC	O1-3
**Rh-Endostatin with Lobaplatin (LBP)**														
Li, H.2016 (93)	NSCLC	Large	PT/RT	≥60	≥3	Yes	50/50	57/43	36–89	30 mg/time,1 time/w, 3 times	50 mg/m^2^	3 weeks	Millar, Un	O1-3
Chen, X.2017 (105)	NSCLC	Moderate to large	Un	≥60	>3	Yes	44/44	54/34	30–89	30 mg/time,1 time/2w, 2 times	30 mg/m^2^	3 years	Millar, Un	O1,3,4
Yin, Y.2020 (29)	MTs	Small to large	Un	Un	>2	Yes	30/30	34/26	35-81	60 mg/time, 2 times/w, 4 times	40 mg/m^2^	2 weeks	Millar, NCICTC	O1-4
**Rh-Endostatin with bleomycin (BLM)**														
Li, G. Y.2011 (57)	MTs	Moderate to large	Un	≥60	Un	Yes	30/30	Un	41–76	30 mg/time, 1 time/w, 3 times	60 mg/m^2^	3 weeks	Millar, NCICTC	O1-3
Zhang, Y.2011 (61)	MTs	Un	Un	≥60	Un	Yes	15/15	18/12	38–73	30 mg/time, 2 times/w, 6 times	60 mg/m^2^	3 weeks	Millar, NCICTC	O1-3
Luo, J.2012 (65)	NSCLC	Un	Un	Un	Un	Yes	34/26	32/28	38–79	60 mg/time, Un, Un	60 mg/m^2^	Un	Millar, NCICTC	O1-3
Zhang, J.2012 (70)	LC	Large	Un	≥60	Un	Yes	24/21	Un	37–76	60 mg/time, 2 times/w, 4 times	40–60 mg/m^2^	6 months	Millar, WHO	O1-3
**Rh-Endostatin with Other(PTX,DDP,BLM)**														
Li, C.2014 (79)	LC	Un	Un	Un	Un	Yes	16/16	21/11	28–69	45 mg/time,1 time/w, 3 times	135–175 mg/m^2^	4 weeks	Millar, Un	O1-3
Li, H.2012 (63)	MTs	Un	Un	Un	Un	Yes	30/30	32/28	46–78	45 mg/time,1 time/w, 4 times	80–100 mg/m^2^; 30–40 mg/m^2^	4 weeks	Millar, Unclear	O1,4

MTs, malignant tumors (lung cancer, breast cancer, malignant lymphoma, gastric cancer, hepatic carcinoma, ovarian cancer, etc.); LC, lung cancer; NSCLC, non-small-cell lung cancer; BC, breast cancer; AST, anticipated survival time; TH, treatment history; E/C, experimental group/control group; F/M, female/male; Experimental group, Endostar plus chemical irritants; Control group, chemical irritants alone; IPCs, indwelling pleural catheters; PTX, paclitaxel; ET, evaluation time; Millar, complete response, partial response, stable disease, and progressive disease (PD); Ostrowskimj, complete response, partial response, and no response. WHO, WHO criteria for adverse drug reactions; Outcomes: O1: clinical responses including complete response, failure, and progressive disease; O2: quality of life (QOL); O3: adverse drug reactions (ADRs) and treatment-related adverse events (TRAEs); O4: survivals.

### Methodological Quality Assessment

**Figure 2:**
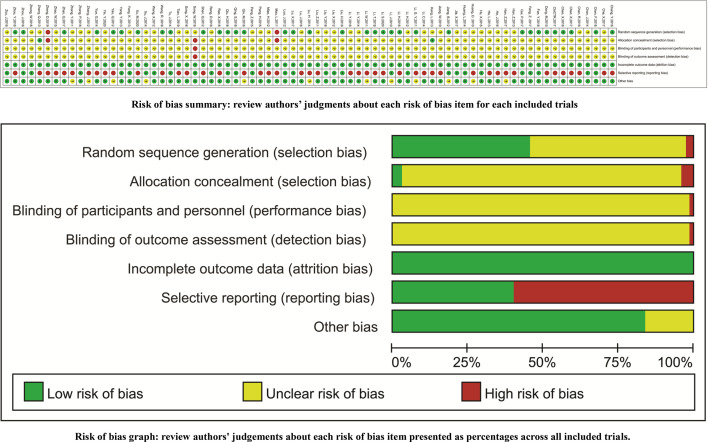
Risk of methodological bias.

Thirty-four trials reported the random sequence generation using a random number table (a low risk of selection bias) (30, 31, 54, 56–58, 62, 65, 67, 69, 75, 81, 82, 84, 85, 89, 90, 93, 97, 101, 102, 105, 106, 109–114, 116, 117, 120, 126, 127), and two trials reported the odd or even random (a high risk of selection bias) (60, 123). Two trials reported the allocation concealment using an envelope (a low risk of selection bias) (73, 76), and two trials reported the allocation exposure (a high risk of selection bias) (60, 123). With the exception of one open RCT (119), the remaining trials failed to clearly report the blindings (an unclear risk of performance bias). All trials reported the complete outcome data (a low risk of attrition bias). Forty-four trials selectively reported the ADRs, and one trial selectively reported the CR (a high risk of reporting bias) (66). The comparability between groups (an unclear risk of other biases) was unclear in 12 trials **(** **Figure 2** **)**.

### Clinical Responses

**Figure 3:**
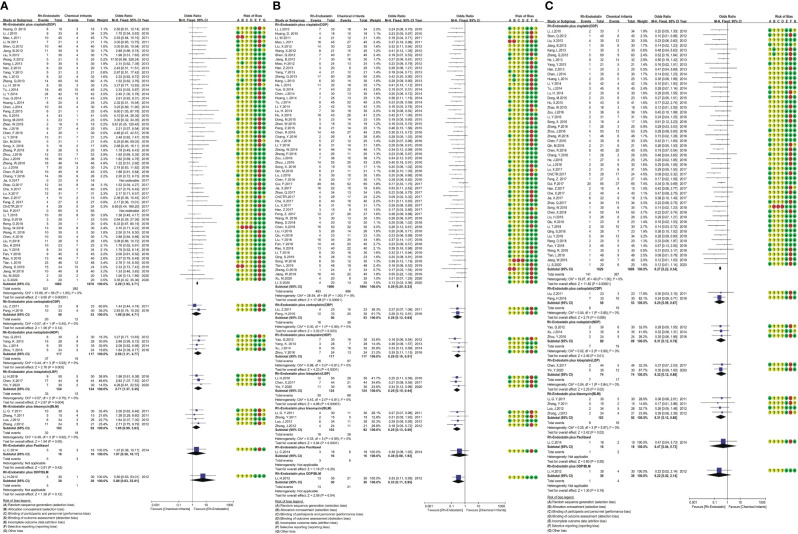
The analysis of clinical responses between the two groups. **(A)** The forest plot of complete response. **(B)** The forest plot of treatment failure. **(C)** The forest plot of progressive disease.

Seventy-five trials reported the clinical responses of six combinations of Rh-endostatin with DDP (30, 31, 54–56, 58, 60, 62, 64, 66–68, 71–73, 75–78, 80–82, 84–92, 94, 95, 97–101, 103, 104, 106–123, 126, 127), NDP (69, 74, 83, 102), BLM (57, 61, 65, 70), LBP (29, 93, 105), CBP (59, 96), paclitaxel (79), or DDP/BLM (63). The statistical heterogeneity was not found using Cochran’s χ^2^ test and I^2^ statistic (I^2^ = 0%). So, the data were pooled using an FEM. The ORs of fixed effects were 2.29 (95% CI 1.93–2.71, p < 0.00001), 2.50 (95% CI 1.31–4.77, p = 0.005), 2.71 (95% CI 1.37–5.35, p = 0.004), which showed that the CR of Rh-endostatin with DDP, NDP, or LBP was significantly higher than that of irritants alone **(** **Figure 3A** **)**. The treatment failure of Rh-endostatin with DDP, CBP, NDP, LBP, or BLM was significantly lower than that of irritants alone. The ORs were 0.29 (95% CI 0.25–0.33, p < 0.00001), 0.28 (95% CI 0.12–0.64, p = 0.003), 0.29 (95% CI 0.16–0.51, p < 0.0001), 0.25 (95% CI 0.15–0.44, p < 0.00001), and 0.25 (95% CI 0.13–0.50, p < 0.0001), respectively **(** **Figure 3B** **)**. The PD of Rh-endostatin with DDP, CBP, NDP, LBP, or BLM was significantly lower than that of irritants alone. The ORs were 0.27 (95% CI 0.22–0.34, p < 0.00001), 0.25 (95% CI 0.09–0.67, p = 0.006), 0.31 (95% CI 0.12–0.79, p = 0.01), 0.32 (95% CI 0.12–0.86, p = 0.02), and 0.31 (95% CI 0.12–0.80, p = 0.02), respectively **(** **Figure 3C** **)**.

### Overall Survival

**Figure 4:**
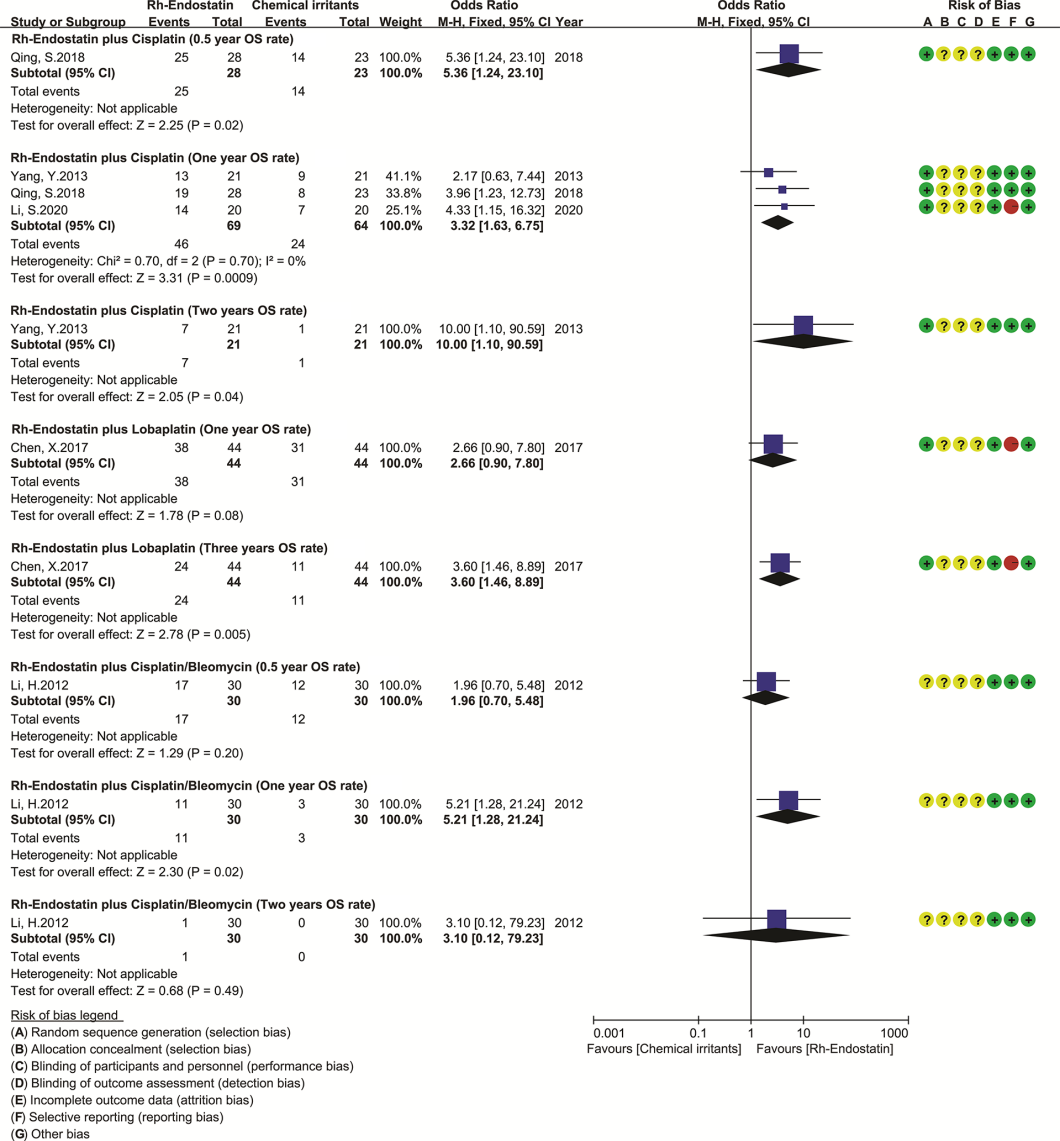
The forest plot of overall survival. DDP, cisplatin; LBP, lobaplatin; BLM, bleomycin; OS, overall survival.

Nine trials reported the survivals (29–31, 63, 71, 75, 98, 105, 116). Only five trials reported the OS time and PFS of Rh-endostatin with DDP (30, 71, 75, 98) or LBP (29), but without the available data. Five trials reported the OS rates, and three reported the 1-year OS rate of Rh-endostatin with DDP (31, 75, 116). The statistical heterogeneity between trials was not found using Cochran’s χ^2^ test and I^2^ statistic (I^2^ = 0%). So, we pooled the data using an FEM. The 1-year OS rate of Rh-endostatin with DDP was significantly higher than that of DDP alone. The OR was 3.32 (95% CI 1.63–6.75, p = 0.0009) **(** **Figure 4** **)**. The remaining OS rates were reported in only one trial, and the data were analyzed descriptively using forest plots. Statistical analysis showed that the 0.5-year OS rate of Rh-endostatin with DDP (116), 1-year OS rate of DDP/BLM (63), 2-year OS rate of DDP (75), and 3-year OS rate of LBP (105) were significantly higher than that of irritants alone. The ORs were 5.36 (95% CI 1.24–23.10, p = 0.02), 5.21 (95% CI 1.28–21.24, p = 0.02), 10.00 (95% CI 2.05–90.59, p = 0.04), and 3.60 (95% CI 1.46–8.89, p = 0.005), respectively.

### Quality of Life

**Figure 5:**
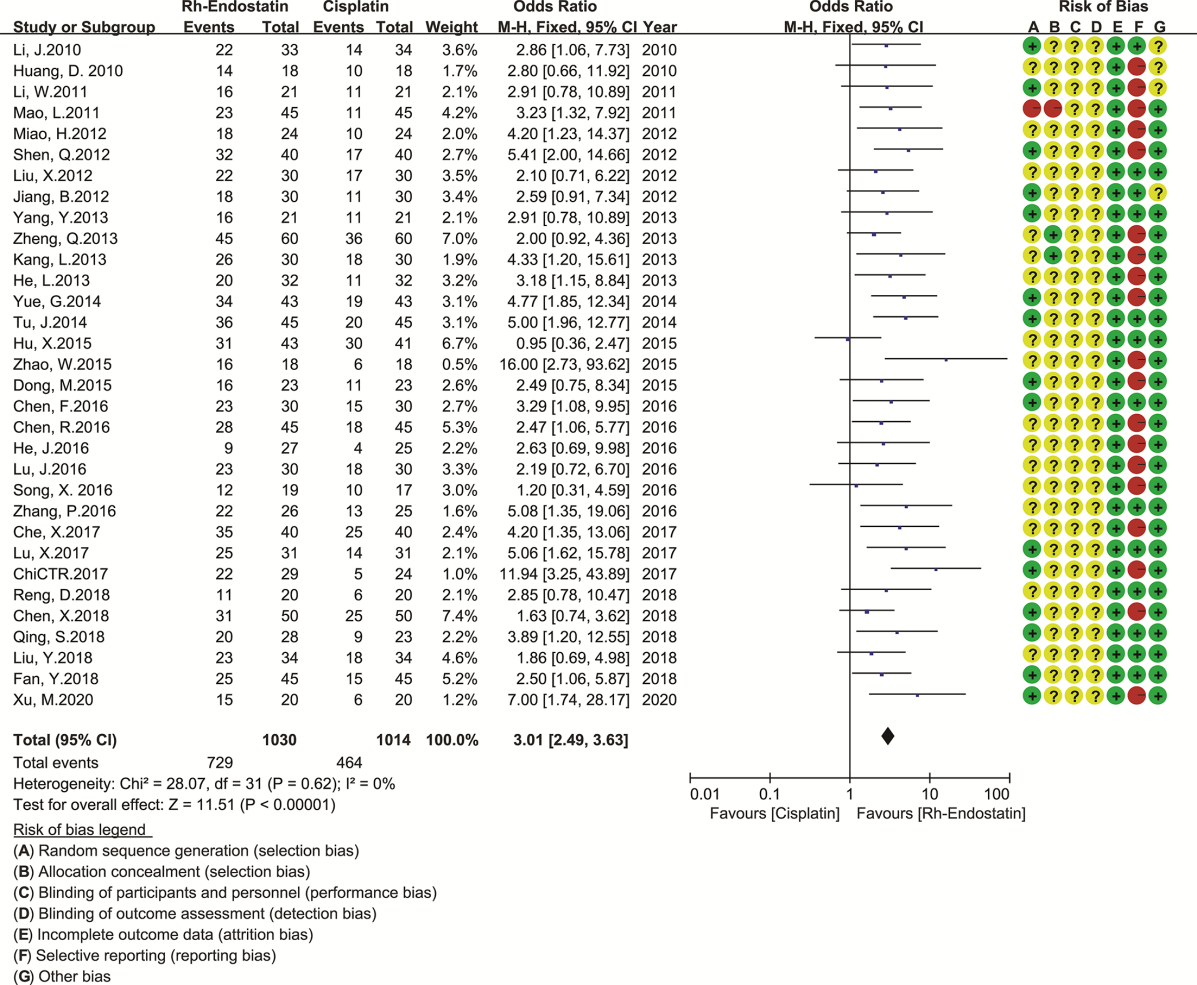
The forest plot of quality of life.

Given the limited trials for Rh-endostatin with CBP, NDP, LBP, or BLM, we only evaluated the QOL of Rh-endostatin with DDP (30, 55, 56, 58, 60, 62, 64, 66, 67, 72, 73, 75, 76, 82, 84–86, 88, 90–92, 95, 98, 99, 104, 109, 111, 112, 115, 116, 118, 126) **(** **Figure 5** **)**. The statistical heterogeneity between trials was not found using Cochran’s χ^2^ test and I^2^ statistic (I^2^ = 0%). So, the data were pooled using an FEM. The OR was 3.01 (95% CI 2.49–3.63, p < 0.00001), which indicated that the QOL was significantly higher than that of DDP alone.

### Adverse Drug Reactions and Treatment-Related Adverse Events

Given the limited trials for Rh-endostatin with CBP, NDP, LBP, or BLM, we only evaluated the ADRs and TRAEs in Rh-endostatin with DDP. Fifty-eight trials observed hematotoxicity (neutropenia, thrombocytopenia, and anemia), cardiotoxicity (arrhythmia), hepatotoxicity, nephrotoxicity, gastrointestinal reaction, alopecia, neurotoxicity, rash, hypertension, hemorrhage, chest pain, and fever (30, 31, 54–56, 58, 60, 62, 64, 66–68, 71–73, 75–78, 80–82, 84–92, 94, 95, 97–100, 103, 104, 107–123, 126, 127) **(** **Table 2** **)**. Cochran’s χ^2^ test and I^2^ statistic showed no heterogeneity in all of the ADRs (I^2^ = 0%). So, the data were pooled using an FEM. Rh-endostatin with DDP had a similar risk of ADRs in DDP alone. The ORs showed no significant difference between the two groups. In addition, nine trials (56, 66, 76, 88, 95, 98, 99, 116, 127) reported no risk of TRAEs, and six trials (66, 88, 95, 98, 99, 116) reported no risk of TRM.

**Table 2:** Meta-analysis results of ADRs and TRAEs (**Figures S1–S11**).

Outcomes	Trials	Events/Total	Events/Total	Statistic method	Odds Ratio, 95%CI	I^2^	p
Neutropenia (**Figure S1**)	32	302/971	299/967	Fixed-effects model	0.98 [0.79, 1.21]	0%	p = 0.83
Thrombocytopenia (**Figure S2**)	28	187/875	178/868	Fixed-effects model	1.04 [0.80, 1.36]	0%	p = 0.76
Anemia (**Figure S3**)	10	49/337	40/339	Fixed-effects model	1.29 [0.80, 2.09]	0%	p = 0.30
Cardiotoxicity (**Figure S4**)	21	37/671	27/672	Fixed-effects model	1.39 [0.84, 2.31]	0%	p = 0.20
Hepatotoxicity (**Figure S5**)	28	91/898	95/900	Fixed-effects model	1.07 [0.77, 1.48]	0%	p = 0.68
Nephrotoxicity (**Figure S6**)	28	91/898	85/900	Fixed-effects model	1.07 [0.77, 1.48]	0%	p = 0.68
Gastrointestinal reactions (**Figure S7**)	49	436/1554	397/1538	Fixed-effects model	1.14 [0.95, 1.36]	0%	p = 0.16
Chest pain (**Figure S8**)	12	51/316	50/321	Fixed-effects model	1.01 [0.63, 1.60]	0%	p = 0.98
Fever (**Figure S9**)	20	67/578	68/573	Fixed-effects model	0.98 [0.68, 1.41]	0%	p = 0.89
Alopecia (**Figure S10a**)	2	3/63	4/61	Fixed-effects model	1.22 [0.33, 4.54]	0%	p = 0.76
Neurotoxicity (**Figure S10b**)	5	8/175	8/173	Fixed-effects model	0.98 [0.36, 2.65]	0%	p = 0.96
Rash (**Figure S10c**)	5	17/193	11/186	Fixed-effects model	1.57 [0.71, 3.50]	0%	p = 0.27
Hypertension (**Figure S10d**)	3	5/92	0/84	Fixed-effects model	4.13 [0.68, 25.10]	0%	p = 0.12
Hamorrhage (**Figure S10e**)	2	4/70	1/66	Fixed-effects model	2.95 [0.45, 19.38]	0%	p = 0.26
Thoracentesis-related adverse events (**Figure S11**)	9	0/303	0/296	No	Not estimable	No	No
Treatment-related mortality (TRM) (**Figure S11**)	6	0/145	0/137	No	Not estimable	No	No

ADRs, adverse drug reactions; TRAEs, thoracentesis-related adverse events; CI, confidence interval.

### Subgroup Analysis of Clinical Responses

The patient feature was defined as primary tumor, pleural fluid volume, treatment history, KPS score, and AST. First, the primary tumor was classified as lung cancer, breast cancer, or malignant tumors. In patients with lung cancer/malignant tumors, Rh-endostatin with DDP obtained a significant increase of CR and a reduction of failure and PD. In breast cancer, it only obtained a reduction of failure and PD **(** **Table 3A** and **Figures S12, S14, S16** **)**. The pleural fluid was classified as small to large, moderate to large, or large **(** **Table 3B** and **Figures S18, S20, S22** **)**; treatment history was initial treatment, retreatment, or both **(** **Table 3C** and **Figures S24, S26, S28** **)**; KPS score was <50, ≥50, or ≥60 **(** **Table 3D** and **Figures S30, S32, S34** **)**; and the AST was ≥2 months or ≥3 months **(** **Table 3E** and **Figures S36, S38, S40** **)**. In patients with moderate to massive effusion, initial treatment, KPS score (≥60), or AST (≥3 months), the Rh-endostatin with DDP groups obtained a significant increase of CR and a reduction of failure and PD.

**Table 3:** Subgroup analysis results (**Supplementary Materials 6A, B**).

Subgroups	Complete response					Treatment failure					Progressive disease				
	Trials	Cases	OR (95%CI)	UM	MM	Trials	Cases	OR (95%CI)	UM	MM	Trials	Cases	OR (95%CI)	UM	MM
**Table 3A. Subgroups analysis *via* primary tumors (** **Figures S12–S17** **)**															
Malignant tumors	19	1172	2.58 [1.89, 3.53]	0.98	0.98	19	1172	0.31 [0.24, 0.40]	0.95	0.76	14	862	0.31 [0.20, 0.46]	0.34	0.46
Lung cancer	37	2393	2.20 [1.79, 2.71]			38	2441	0.28 [0.23, 0.34]			33	2189	0.26 [0.20, 0.33]		
Breast cancer	3	187	1.85 [0.91, 3.75]			3	187	0.23 [0.12, 0.44]			3	187	0.35 [0.12, 1.02]		
**Table 3B. Subgroups analysis *via* pleural fluid volume (** **Figures S18–S23** **)**															
Small to large	2	97	2.08 [0.68, 6.36]	0.44	0.70	2	97	0.20 [0.08, 0.51]	0.82	0.85	2	97	0.28 [0.07, 1.11]	0.89	0.62
Moderate to large	33	2032	2.46 [1.94, 3.12]			34	2080	0.29 [0.24, 0.35]			30	1860	0.27 [0.20, 0.35]		
Large	5	326	2.35 [1.30, 4.25]			5	326	0.27 [0.17, 0.44]			2	110	0.45 [0.12, 1.62]		
Unclear	19	1297	2.08 [1.59, 2.72]			19	1297	0.29 [0.23, 0.38]			16	1171	0.27 [0.19, 0.39]		
**Table 3C. Subgroups analysis *via* treatment history (** **Figures S24–S29** **)**															
Initial and retreatment	5	242	2.47 [1.03, 5.91]	0.44	0.58	5	242	0.33 [0.19, 0.57]	0.95	0.82	2	124	0.54 [0.22, 1.35]	0.19	0.30
Initial treatment	7	484	2.72 [1.68, 4.39]			7	484	0.26 [0.17, 0.38]			4	228	0.28 [0.12, 0.63]		
Unclear	47	3026	2.22 [1.85, 2.67]			48	3074	0.29 [0.25, 0.34]			44	2886	0.26 [0.21, 0.33]		
**Table 3D. Subgroups analysis *via* KPS score (** **Figures S30–S35** **)**															
KPS score(<50)	1	53	8.65 [0.44, 169.20]	0.78	0.95	1	53	0.09 [0.02, 0.32]	0.25	0.28	1	53	0.09 [0.02, 0.32]	0.71	0.98
KPS score(≥50)	4	202	2.28 [1.00, 5.18]			4	202	0.24 [0.13, 0.45]			3	162	0.29 [0.09, 0.89]		
KPS score(≥60)	37	2478	2.30 [1.88, 2.81]			38	2526	0.29 [0.24, 0.35]			31	2098	0.30 [0.23, 0.39]		
Unclear	17	1019	2.21 [1.58, 3.08]			17	1019	0.30 [0.23, 0.40]			15	925	0.25 [0.17, 0.36]		
**Table 3E.Subgroups analysis *via* anticipated survival time (** **Figures S36–S41** **)**															
AST (≥2months)	5	299	2.47 [1.36, 4.48]	0.78	0.87	6	347	0.24 [0.15, 0.40]	0.80	0.76	4	259	0.30 [0.13, 0.66]	0.40	0.51
AST (≥3months)	36	2483	2.26 [1.84, 2.79]			36	2483	0.29 [0.25, 0.35]			32	2181	0.29 [0.22, 0.37]		
AST (unclear)	18	970	2.29 [1.66, 3.16]			18	970	0.28 [0.21, 0.37]			14	798	0.23 [0.15, 0.36]		
**Table 3F.Subgroups analysis *via* indwelling pleural catheters (** **Figures S42–S47** **)**															
Yes	53	3369	2.29 [1.92, 2.74]	0.84	0.84	54	3417	0.29 [0.25, 0.34]	0.72	0.79	45	2941	0.29 [0.23, 0.36]	0.05	0.12
No	6	383	2.26 [1.36, 3.76]			6	383	0.26 [0.17, 0.41]			5	297	0.13 [0.06, 0.30]		
**Table 3G. Subgroups analysis *via* Rh-Endostatin dosage (** **Figures S48–S53** **)**															
30 to 35mg	18	1134	2.37 [1.72, 3.27]	0.67	0.78	18	1134	0.29 [0.22, 0.38]	0.67	0.78	15	920	0.30 [0.21, 0.43]	0.97	0.57
40-45mg	26	1687	2.26 [1.77, 2.87]			26	1687	0.29 [0.23, 0.36]			21	1427	0.23 [0.16, 0.33]		
60-90mg	13	842	2.17 [1.53, 3.10]			13	842	0.31 [0.23, 0.42]			12	802	0.35 [0.22, 0.55]		
Others	2	89	4.05 [0.91, 18.10]			3	137	0.17 [0.08, 0.35]			2	89	0.18 [0.07, 0.49]		
**Table 3H. Subgroups analysis *via* treatment frequency (** **Figures S54–S59** **)**															
Once a week	17	1055	2.31 [1.67, 3.20]	0.97	0.79	18	1103	0.29 [0.23, 0.38]	0.57	0.56	13	851	0.28 [0.18, 0.43]	0.43	0.24
Twice a week	32	2013	2.28 [1.80, 2.87]			32	2013	0.29 [0.24, 0.36]			28	1749	0.30 [0.23, 0.39]		
Unclear	10	684	2.28 [1.58, 3.31]			10	684	0.25 [0.17, 0.36]			9	638	0.17 [0.09, 0.32]		
**Table 3I. Subgroups analysis *via* treatment times (** **Figures S60-65** **)**															
One to two times	4	194	3.07 [1.52, 6.22]	0.48	0.52	4	194	0.30 [0.16, 0.57]	0.85	0.68	3	148	0.34 [0.11, 1.06]	0.62	0.54
Three to four times	23	1353	2.35 [1.77, 3.13]			24	1401	0.29 [0.23, 0.37]			16	971	0.27 [0.18, 0.40]		
Five to six times	10	610	2.42 [1.61, 3.64]			10	610	0.24 [0.17, 0.34]			10	610	0.19 [0.11, 0.33]		
Unclear	22	1595	2.10 [1.62, 2.71]			22	1595	0.30 [0.24, 0.38]			21	1509	0.31 [0.23, 0.41]		
**Table 3J. Subgroups analysis *via* cisplatin dosage (** **Figures S66–S71** **)**															
30 to 40mg/m^2^	29	1892	2.23 [1.77, 2.81]	0.87	0.84	30	1940	0.28 [0.23, 0.34]	0.88	0.88	25	1672	0.25 [0.18, 0.34]	0.57	0.34
50 to 60mg/m^2^	24	1553	2.30 [1.77, 2.98]			24	1553	0.31 [0.24, 0.38]			20	1299	0.29 [0.21, 0.41]		
Others	6	307	2.83 [1.35, 5.95]			6	307	0.23 [0.14, 0.38]			5	267	0.30 [0.15, 0.59]		

AST, anticipated survival time; KPS score, Karnofsky Performance Status score; OR, odds ratio; Rh-endostatin, recombinant human endostatin; CI, confidence interval; UM, univariable meta-regression; MM, multiple meta-regression.

The majority of patients mainly received the IPCs **(** **Table 3F** and **Figures S42, S44, S46** **)**. Subgroup analyses found that whether IPC is used or not had no effect on the clinical responses. Rh-endostatin was used with 30–90 mg each time, once or twice a week 1–12 times **(** **Tables 3G–I** and **Figures S48–S64** **)**. DDP was used with 30–40 mg/m^2^ or 50–60 mg/m^2^ each time **(** **Table 3J** and **Figures S66, S68, S70** **)**. Rh-endostatin (30–35 mg or 40–45 mg each time, once or twice a week 3–4 times) with DDP (30–40 mg/m^2^ or 50–60 mg/m^2^) obtained a significant increase of response and a reduction of failure and PD in MPE. However, univariate regression analysis did not discover a positive or negative correlation between CR, treatment failure, and PD and each variable **(** **Table 3** and **Figures S13–S71** **).** Multiple meta-regression analysis also did not discover a positive or negative correlation **(** **Table 3** **)**.

### Publication Bias Analysis

**Figure 6:**
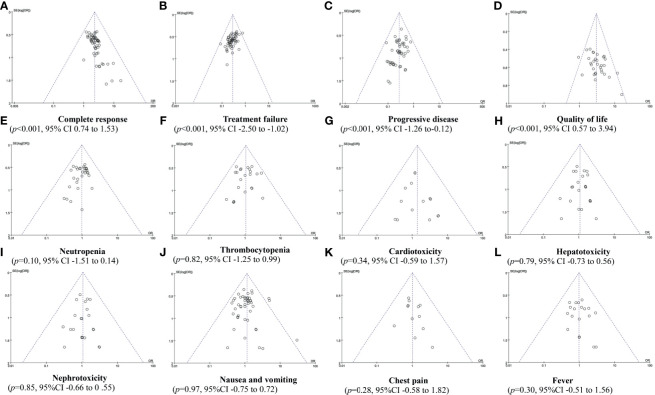
The publication bias analysis.

In Rh-endostatin with DDP, more than 10 trials were included for the CR, treatment failure, PD, QOL, and ADRs. So, funnel plot and Egger/Begg’s tests were used to analyze their potential bias of publication. The analysis found a publication bias in CR (p < 0.001, 95% CI 0.74–1.53), treatment failure (p < 0.001, 95% CI -2.50 to -1.02), PD (p < 0.001, 95% CI -1.26 to -0.12), and QOL (p < 0.001, 95% CI 0.57–3.94) **(** **Figures 6A–D** **)**. The trials overestimated the CR and QOL and underestimated the treatment failure and PD. The analysis did not find a bias in neutropenia (p = 0.10, 95% CI -1.51 to 0.14), thrombocytopenia (p = 0.82, 95% CI -1.25 to 0.99), cardiotoxicity (p = 0.34, 95% CI -59 to 1.57), hepatotoxicity (p = 0.79, 95% CI -0.73 to 0.56), nephrotoxicity (p = 0.85, 95% CI -0.66 to 0.55), gastrointestinal reactions (p = 0.97, 95% CI -0.75 to 0.72), chest pain (p = 0.28, 95% CI -0.58 to 1.82), and fever (p = 0.30, 95% CI -0.51 to 0.14) (**Figures 6E–L**). The trials objectively reported the ADRs.

### Sensitivity Analysis

In Rh-endostatin with DDP, the poor trials involved clinical response, 1-year OS rate, QOL, and ADRs. Some trials overestimated the CR, 1-year OS rate, and QOL and underestimated the treatment failure and PD. According to the underestimating efficacy and overestimating risk, we evaluated the robustness through removing the poor trials, overestimation/underestimation, and both. Before and after removing the poor trials, the results demonstrated a good robustness of all outcomes. Before and after removing the overestimation and both, the OR of 1-year OS rate was poor robust, and other indicators were robust. In addition, the OR of CR was robust in Rh-endostatin with NDP, and the OR of CR and PD was robust in Rh-endostatin with BLM (**Table 4**).

**Table 4:** Sensitivity analysis.

Indicators	Before excluding trials				Excluded poor trials				Excluded over/under-estimation*				Excluded poor,over/under-estimation*			
	Trials	SM	OR(95%CI)	I^2^	Trials	SM	OR (95%CI)	I^2^	Trials	SM	OR (95%CI)	I^2^	Trials	SM	OR (95%CI)	I^2^
**Rh-Endostatin with Cisplatin**																
Complete response	59	FEM	2.29 [1.93, 2.71]	0%	25	FEM	2.28 [1.74, 2.98]	0%	56	FEM	2.24 [1.88, 2.67]	0%	24	FEM	2.21 [1.69, 2.90]	0%
Treatment failure	60	FEM	0.29 [0.25, 0.33]	0%	25	FEM	0.32 [0.26, 0.39]	0%	17	FEM	0.41 [0.31, 0.54]	0%	8	FEM	0.45 [0.31, 0.66]	0%
Progressive disease	50	FEM	0.27 [0.22, 0.34]	0%	23	FEM	0.31 [0.23, 0.42]	0%	39	FEM	0.31 [0.24, 0.41]	0%	19	FEM	0.32 [0.22, 0.46]	0%
Quality of life	32	FEM	3.01 [2.49, 3.63]	0%	13	FEM	2.76 [2.06, 3.69]	0%	14	FEM	1.99 [1.50, 2.66]	0%	6	FEM	1.93 [1.24, 2.99]	0%
1 OS rate	3	FEM	3.32 [1.63, 6.75]	0%	2	FEM	2.98 [1.28, 6.92]	0%	1	no	2.17 [0.63, 7.44]	no	1	no	2.17 [0.63, 7.44]	no
Neutropenia	32	FEM	0.98 [0.79, 1.21]	0%	18	FEM	1.08 [0.81, 1.43]	0%	32	FEM	0.98 [0.79, 1.21]	0%	18	FEM	1.08 [0.81, 1.43]	0%
Thrombocytopenia	28	FEM	1.04 [0.80, 1.36]	0%	13	FEM	1.07 [0.71, 1.59]	0%	28	FEM	1.04 [0.80, 1.36]	0%	13	FEM	1.07 [0.71, 1.59]	0%
Anemia	10	FEM	1.29 [0.80, 2.09]	0%	9	FEM	1.45 [0.84, 2.50]	0%	10	FEM	1.29 [0.80, 2.09]	0%	9	FEM	1.45 [0.84, 2.50]	0%
Gastrointestinal reactions	49	FEM	1.14 [0.95, 1.36]	0%	22	FEM	1.03 [0.80, 1.34]	0%	49	FEM	1.14 [0.95, 1.36]	0%	22	FEM	1.03 [0.80, 1.34]	0%
Hepatotoxicity	28	FEM	1.07 [0.77, 1.48]	0%	21	FEM	0.98 [0.67, 1.44]	0%	28	FEM	1.07 [0.77, 1.48]	0%	21	FEM	0.98 [0.67, 1.44]	0%
Nephrotoxicity	28	FEM	1.06 [0.74, 1.53]	0%	21	FEM	0.94 [0.62, 1.44]	0%	28	FEM	1.06 [0.74, 1.53]	0%	21	FEM	0.94 [0.62, 1.44]	0%
Cardiotoxicity	21	FEM	1.06 [0.74, 1.53]	0%	19	FEM	1.39 [0.84, 2.31]	0%	21	FEM	1.06 [0.74, 1.53]	0%	19	FEM	1.39 [0.84, 2.31]	0%
Fever	20	FEM	0.98 [0.68, 1.41]	0%	10	FEM	1.04 [0.61, 1.76]	0%	20	FEM	0.98 [0.68, 1.41]	0%	10	FEM	1.04 [0.61, 1.76]	0%
Thoracodynia	12	FEM	1.01 [0.63, 1.60]	0%	5	FEM	0.92 [0.44, 1.93]	0%	12	FEM	1.01 [0.63, 1.60]	0%	5	FEM	0.92 [0.44, 1.93]	0%
Neurotoxicity	5	FEM	0.98 [0.36, 2.65]	0%	4	FEM	0.77 [0.21, 2.83]	0%	5	FEM	0.98 [0.36, 2.65]	0%	4	FEM	0.77 [0.21, 2.83]	0%
Hemorrhage	2	FEM	2.95 [0.45, 19.38]	0%	1	no	5.00 [0.23,107.35]	no	2	FEM	2.95[0.45,19.38]	0%	1	no	5.00[0.23,107.35]	no
Hypertension	3	FEM	4.13 [0.68, 25.10]	0%	2	FEM	3.72 [0.40, 34.48]	0%	3	FEM	4.13 [0.68,25.10]	0%	2	FEM	3.72 [0.40, 34.48]	0%
**Rh-Endostatin with Nedaplatin**																
Complete response	4	FEM	2.50 [1.31, 4.77]	0%	2	FEM	2.48 [1.10, 5.62]	0%	4	FEM	2.50 [1.31, 4.77]	0%	2	FEM	2.48 [1.10, 5.62]	0%
Treatment failure	4	FEM	0.29 [0.16, 0.51]	0%	2	FEM	0.34 [0.15, 0.78]	0%	2	FEM	0.35 [0.14, 0.89]	0%	1	No	0.36 [0.08, 1.57]	No
Progressive disease	3	FEM	0.31 [0.12, 0.79]	0%	1	No	0.32 [0.08, 1.31]	No	3	FEM	0.31 [0.12, 0.79]	0%	1	No	0.32 [0.08, 1.31]	No
**Rh-Endostatin with Bleomycin**																
Complete response	4	FEM	1.73 [0.89, 3.37]	0%	1	No	1.64 [0.37, 7.30]	No	4	FEM	1.73 [0.89, 3.37]	0%	1	No	1.64 [0.37, 7.30]	No
Treatment failure	4	FEM	0.38 [0.20, 0.72]	0	1	No	0.29 [0.09, 0.95]	No	1	No	3.25 [0.52,20.37]	No	No	No	No	No
Progressive disease	4	FEM	0.49 [0.20, 1.19]	0	1	No	0.26 [0.05, 1.48]	No	4	FEM	0.49 [0.20, 1.19]	0	1	No	0.26 [0.05, 1.48]	No

SM, statistical method; FEM, fixed-effects model; REM, random-effects model; OR, odds ratio; OS, overall survival; CI, confidence interval; Poor*, poor trials that had at least one domain being considered as high risk of bias; Over* or Under*, overestimated or underestimated trials in which results had significant differences and beneficial to Rh-endostatin group.

### Quality of Evidence

In methodology, 46 poor trials were included for this analysis. Sensitivity analysis demonstrated that the OR of 1-year OS rate was poor robustness in Rh-endostatin with DDP, the CR and PD were poor in Rh-endostatin with NDP, and the CR was poor in Rh-endostatin with BLM. Therefore, we downgraded their quality by two grades. Other results had good robustness, and we downgraded their quality by one grade. No heterogeneity was found in all of the indicators; all indicators were not downgraded. In Rh-endostatin with DDP, the sample size of 1-year OS rate, alopecia, hypertension, and hemorrhage was lower than 300 subjects. In Rh-endostatin with NDP, CBP, LBP, or BLM, the CR, treatment failure, and PD were lower than 300. So, we downgraded their quality by one grade. In addition, the funnel plot and Egger’s test showed a publication bias of CR, treatment failure, PD, and QOL in Rh-endostatin with DDP. The sensitivity analysis results were good robust, and we did not downgrade their quality. So, we summarized a low quality for 1-year OS rate, alopecia, hypertension, and hemorrhage and a moderate quality for other results of Rh-endostatin with DDP; a low quality for CR and treatment failure in Rh-endostatin with NDP or BLM; and a very low quality for the remaining indicators (**Table 5**).

**Table 5:** GRADE evidence profile.

Indicators (Trials)	Quality assessment					Malignant pleural effusion		Clinical efficacy and safety		Quality
	Risk of bias	Inconsistency	Indirectness	Imprecision	Publication bias	Rh-endostatin	Chemical irritants	Odds ratios (95% CI)	Absolute effects	
**Rh-Endostatin with Cisplatin**										
Complete response (59)	Serious^1^	None	None	None	None^2^	521/1882 (27.7%)	282/1870 (15.1%)	2.29(1.93 to 2.71)	138 more per 1000 (from 104 more to 174 more)	ÅÅÅO
Treatment Failure (60)	Serious^1^	None	None	None	None^3^	493/1906 (25.9%)	996/1894 (52.6%)	0.29(0.25 to 0.33)	283 fewer per 1000 (from 258 fewer to 309 fewer)	ÅÅÅO
progressive disease (50)	Serious^1^	None	None	None	None^3^	141/1629 (8.7%)	387/1609 (24.1%)	0.27(0.22 to 0.34)	162 fewer per 1000 (from 143 fewer to 175 fewer)	ÅÅÅO
One year OS rate (3)	Serious^1^	None	None	Serious^4^	None	46/69 (66.7%)	24/64 (37.5%)	3.32(1.63 to 6.75)	291 more per 1000 (from 119 more to 427 more)	ÅÅOO
Quality of life (32)	Serious^1^	None	None	None	None^2^	729/1030 (70.8%)	464/1014 (45.8%)	3.01(2.49 to 3.63)	260 more per 1000 (from 220 more to 296 more)	ÅÅÅO
Neutropenia (32)	Serious^1^	None	None	None	None	302/971 (31.1%)	299/967 (30.9%)	0.98(0.79 to 1.21)	4 fewer per 1000 (from 48 fewer to 42 more)	ÅÅÅO
Thrombocytopenia (28)	Serious^1^	None	None	None	None	187/875 (21.4%)	178/868 (20.5%)	1.04(0.8 to 1.36)	6 more per 1000 (from 34 fewer to 55 more)	ÅÅÅO
Thrombocytopenia (10)	Serious^1^	None	None	None	None	49/337 (14.5%)	40/339 (11.8%)	1.29(0.8 to 2.09)	29 more per 1000 (from 21 fewer to 101 more)	ÅÅÅO
Cardiotoxicity (21)	Serious^1^	None	None	None	None	37/671 (5.5%)	27/672 (4%)	1.39(0.84 to 2.31)	15 more per 1000 (from 6 fewer to 48 more)	ÅÅÅO
Hepatotoxicity (28)	Serious^1^	None	None	None	None	91/898 (10.1%)	85/900 (9.4%)	1.07(0.77 to 1.48)	6 more per 1000 (from 20 fewer to 39 more)	ÅÅÅO
Nephrotoxicity (28)	Serious^1^	None	None	None	None	68/886 (7.7%)	65/890 (7.3%)	1.06(0.74 to 1.53)	4 more per 1000 (from 18 fewer to 35 more)	ÅÅÅO
Nausea and vomiting (49)	Serious^1^	None	None	None	None	436/1554 (28.1%)	397/1538 (25.8%)	1.14(0.95 to 1.36)	26 more per 1000 (from 10 fewer to 63 more)	ÅÅÅO
Chest pain (12)	Serious^1^	None	None	None	None	51/316 (16.1%)	50/321 (15.6%)	1.01(0.63 to 1.6)	1 more per 1000 (from 52 fewer to 72 more)	ÅÅÅO
Fever (20)	Serious^1^	None	None	None	None	67/578 (11.6%)	68/573 (11.9%)	0.98(0.68 to 1.41)	2 fewer per 1000 (from 35 fewer to 41 more)	ÅÅÅO
Alopecia (2)	Serious^5^	None	None	Serious^4^	None	5/63 (7.9%)	4/61 (6.6%)	1.22(0.33 to 4.54)	13 more per 1000 (from 43 fewer to 176 more)	ÅÅOO
Neurotoxicity (5)	Serious^1^	None	None	None	None	8/175 (4.6%)	8/173 (4.6%)	0.98(0.36 to 2.65)	1 fewer per 1000 (from 29 fewer to 68 more)	ÅÅÅO
Rash (5)	Serious^5^	None	None	None	None	17/193 (8.8%)	11/186 (5.9%)	1.57(0.71 to 3.5)	31 more per 1000 (from 16 fewer to 121 more)	ÅÅÅO
Hypertension (3)	Serious^1^	None	None	Serious^4^	None	5/92 (5.4%)	0/84 (0%)	4.13(0.68 to 25.1)	none	ÅÅOO
Hemorrhage (2)	Serious^1^	None	None	Serious^4^	None	4/70 (5.7%)	1/66 (1.5%)	2.95(0.45 to 19.5)	28 more per 1000 (from 8 fewer to 215 more)	ÅÅOO
**Rh-Endostatin with Nedaplatin**										
Complete response (4)	Serious^1^	None	None	Serious^4^	None	37/117 (31.6%)	19/117 (16.2%)	2.5(1.31 to 4.77)	164 more per 1000 (from 40 more to 318 more)	ÅÅOO
Treatment failure (4)	Serious^1^	None	None	Serious^4^	None	26/117 (22.2%)	57/117 (48.7%)	0.29(0.16 to 0.51)	271 fewer per 1000 (from 161 fewer to 355 fewer)	ÅÅOO
Progressive disease (3)	Very serious^6^	None	None	Serious^4^	None	7/89 (7.9%)	19/89 (21.3%)	0.31(0.12 to 0.79)	136 fewer per 1000 (from 37 fewer to 182 fewer)	ÅOOO
**Rh-Endostatin with Carboplatin**										
Complete response (2)	Very serious^7^	None	None	Serious^4^	None	20/56 (35.7%)	12/53 (22.6%)	1.99(0.84 to 4.71)	142 more per 1000 (from 29 fewer to 353 more)	ÅOOO
Treatment failure (2)	Very serious^7^	None	None	Serious^4^	None	16/56 (28.6%)	30/53 (56.6%)	0.28(0.12 to 0.64)	299 fewer per 1000 (from 111 fewer to 431 fewer)	ÅOOO
Progressive disease (2)	Very serious^7^	None	None	Serious^4^	None	8/56 (14.3%)	19/53 (35.8%)	0.25(0.09 to 0.67)	236 fewer per 1000 (from 86 fewer to 311 fewer)	ÅOOO
**Rh-Endostatin with Lobaplatin**										
Complete response (3)	Very serious^7^	None	None	Serious^4^	None	33/124 (26.6%)	15/124 (12.1%)	2.71(1.37 to 5.35)	151 more per 1000 (from 38 more to 303 more)	ÅOOO
Treatment failure (3)	Very serious^7^	None	None	Serious^4^	None	30/124 (24.2%)	68/124 (54.8%)	0.25(0.15 to 0.44)	316 fewer per 1000 (from 200 fewer to 394 fewer)	ÅOOO
Progressive disease (2)	Very serious^7^	None	None	Serious^4^	None	7/74 (9.5%)	17/74 (23%)	0.32(0.12 to 0.86)	143 fewer per 1000 (from 26 fewer to 195 fewer)	ÅOOO
**Rh-Endostatin with Bleomycin**										
Complete response (4)	Serious^1^	None	None	Serious^4^	None	32/103 (31.1%)	18/92 (19.6%)	1.95(0.99 to 3.83)	126 more per 1000 (from 2 fewer to 287 more)	ÅÅOO
Treatment failure (4)	Serious^1^	None	None	Serious^4^	None	16/103 (15.5%)	38/92 (41.3%)	0.25(0.13 to 0.5)	263 fewer per 1000 (from 153 fewer to 329 fewer)	ÅÅOO
Progressive disease (4)	Very serious^6^	None	None	Serious^4^	None	6/103 (5.8%)	16/92 (17.4%)	0.31(0.12 to 0.8)	113 fewer per 1000 (from 30 fewer to 149 fewer)	ÅOOO

CI, confidence interval; OS, overall survival;,Rh-endostatin, recombinant human endostatin.^1^Most trials had an unclear risk, and some trials had a high risk. If good robustness, we downgraded it by one grade. ^2^Publication bias was found in them, and the result was overestimated. The result showed good robustness and was not downgraded. ^3^Publication bias was found in them, and the result was underestimated. The result showed good robustness and not downgraded. ^4^The number of patients in each result was less than 300, and we downgraded it by one grade. ^5^Most trials had an unclear risk and no high risk, and we downgraded it by one grade. ^6^Most trials had an unclear risk, and some trials had a high risk. If sensitivity analysis results had poor robustness, we downgraded it by two grades. ^7^All trials had a high risk, and we downgraded it by two grades.

## Discussion

Intrapleural administration of Rh-endostatin alone or plus chemical irritants is recommended for the control of MPE by expert consensus from China (28). To demonstrate the optimal combinations of Rh-endostatin with chemical irritants and their clinical efficacy and safety, we further included 75 trials for analysis (29–31, 54–123, 126, 127). In this study, we found six combinations such as Rh-endostatin with DDP, CBP, NDP, LBP, BLM, or paclitaxel. The results of meta-analysis demonstrated that the Rh-endostatin with DDP might improve the response and reduce the failure and PD, with “moderate” quality. We further found that this combination might also improve the QOL, without increasing the risk of hematotoxicity, cardiotoxicity, hepatotoxicity, nephrotoxicity, gastrointestinal reaction, chest pain, and fever, with “moderate” quality. In addition, there were limited reports on the combinations of Rh-endostatin with CBP, NDP, LBP, BLM, or paclitaxel. Only the combinations Rh-endostatin with NDP and LBP might increase the response and reduce the failure and PD, but with “low to very low” quality. A few trials reported the survival; only Rh-endostatin with DDP or LBP might improve 1- to 2-year OS rate, with “low to very low” quality. And most trials failed to report the TRAEs and TRM. Evidently, these outcomes are not fully evaluated and need to be further confirmed.

**Figure 7:**
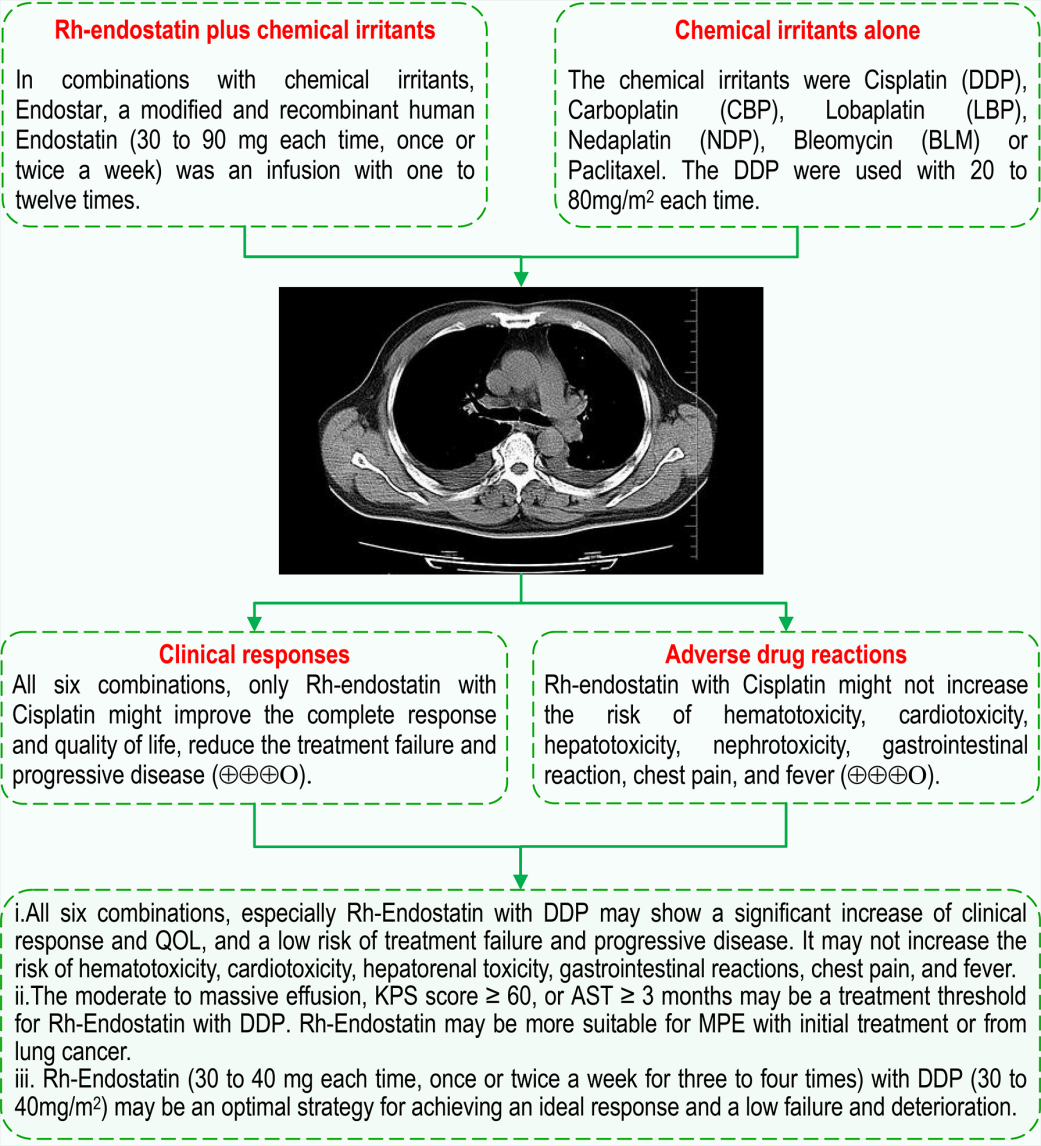
Intrapleural infusion with Rh-endostatin for MPE. AST, anticipated survival time, QOL, quality of life; KPS, Karnofsky Performance Status; MPE, malignant pleural effusion.

Eight previous evaluations had reported that the intrathoracic infusion with Rh-endostatin combined with platinum (17, 18), DDP (19–23), or chemotherapeutic agents (24) might improve the objective response rate, disease control rate, and QOL without an increase in the incidence of ADRs in MPEs from malignant tumors. Rh-endostatin with DDP might also obtain the same effects in MPEs from lung cancer (25–27). In this evaluation, we redefined the clinical efficacy as CR, treatment failure, PD, and survival and added the TRAEs and TRM as security indexes, further integrated previous studies (17–27), and added 36 trials with 2,209 patients for analysis. This evaluation found that all six combinations, especially Rh-endostatin with DDP, might show an improvement of clinical response and a reduction of failure and PD, without an increase of the ADRs. The result indicates that a significant synergistic effect exists between Rh-endostatin and DDP. In clinical practice, the BRMs (38, 128) and TCMIs (129–131) were also used in the control of MPEs through intrathoracic infusion. Previous studies (129–131) had reported that chemical irritants plus TCMIs might increase the clinical benefit rate and decrease the ADRs. Chemical irritants plus BRMs (38, 128) also obtain the same benefit. But compared with TCMIs and BRMs, Rh-endostatin did not reduce the risk of ADRs, which may limit its clinical application. All in all, the results indicate that intrapleural administration of TCMIs, BRMs, or Rh-endostatin might be an important pathway to perform pleurodesis and control the hydrothorax **(** **Figure 7** **)**.

In a previous analysis (38), we found that moderate to large pleural fluid, KPS scores ≥50, or AST ≥3 months might be the treatment thresholds for lentinan with DDP. So, we performed a series of subgroup analyses to reveal the therapeutic thresholds and optimal usage of Rh-endostatin with DDP for achieving a desired response and security. Our analyses found that MPE patients with lung cancer, moderate to massive effusion, initial treatment, KPS score ≥60, or AST ≥3 months might be more suitable for Rh-endostatin with DDP infusion than patients with other conditions. The infusion conditions, the volume of pleural effusion, treatment history, and AST are the same as that of lentinan with DDP infusion. But Rh-endostatin infusion requires a higher KPS (≥60) than lentinan infusion, which suggests that Rh-endostatin infusion seems to have a higher threshold than lentinan. Yoon et al. (132) had reported that poor performance status [Eastern Cooperative Oncology Group (ECOG) 3 or 4] was an independent risk factor of poor survival after video-assisted thoracic surgery (VATS) talc pleurodesis. Compared with VATS talc pleurodesis, endostatin infusion seems to have a lower threshold. In all, the results indicate that endostatin seems to have a special threshold for infusion. The moderate to massive effusion, KPS score ≥60, or AST ≥3 months may be a treatment threshold for Rh-endostatin with DDP, which may be more suitable for MPE with initial treatment or for lung cancer. So, the objective assessment of patients’ baseline should be considered when choosing Rh-endostatin with DDP. In expert consensus (28), Rh-endostatin (45 mg each time) with DDP (40 mg/m^2^) is recommended to control MPEs. Further subgroup analysis revealed that Rh-endostatin (30–35 mg or 40–45 mg each time, once or twice a week 3–4 times) with DDP (30–40 mg/m^2^ or 50–60 mg/m^2^) obtained a significant increase of clinical response and a reduction of failure and PD. Based on the low dose and cost matching, we believe that Rh-endostatin (30–40 mg each time, once or twice a week 3–4 times) with DDP (30–40 mg/m^2^) may be a possible strategy for achieving an ideal response and a low failure and deterioration **(** **Figure 7** **)**. The dose of Rh-endostatin and DDP may be lower than the recommended dose (28). All these findings demonstrate a possible treatment threshold and optimum strategy of intrapleural administration of Rh-endostatin with DDP for MPEs, which is of important clinical significance for further improving scientific decision-making of drug rational application. But the meta-regressions did not further confirm the positive or negative correlation. In addition, whether endostatin with DDP infusion is suitable for drug-resistant, refractory, retreatment, or recurrent MPEs and MPEs from other tumors remains unclear. For Rh-endostatin with CBP, NDP, or LBP/BLM, the treatment threshold and optimal strategy remain unclear. So, these questions need to be further answered.

All kinds of potential limitations should be taken into consideration. First, in this study, only Chinese and English databases were searched, which might result in potential retrieval biases. Second, a considerable number of trials did not clearly describe the baseline features such as the volume of hydrothorax, KPS score, AST, initial treatment, retreatment, drug-resistant, refractory, or recurrent. Third, only 34 studies described the generation of random sequence, and 44 studies selectively reported the CR, ADRs, or TRAEs. Fourth, there was lack of a unified standard for clinical efficacy of chemical pleurodesis in MPEs, and the majority of trials did not clearly report the survivals, TRAEs, and TRM. Fifth, due to limited trials for Rh-endostatin with CBP, NDP, LBP/BLM, the treatment thresholds and optimal strategy remain unclear. Sixth, the univariate or multivariate regression analysis did not find any positive or negative correlation between clinical responses and all variables.

## Conclusion

The evidence indicates that among all six combinations, only Rh-endostatin with DDP may be an optimal combination, which may improve the clinical response and QOL and reduce the failure and PD without increasing the ADRs in MPEs. For Rh-endostatin with DDP infusion, the treatment threshold may be moderate to massive effusion, KPS score ≥ 60, or AST ≥3 months. The combination may be more suitable for MPE with initial treatment or for lung cancer. Rh-endostatin (30–40 mg each time, once or twice a week 3–4 times) with DDP (30–40 mg/m^2^) may be a possible strategy for achieving an ideal response. The pooled results from limited trials reveal that Rh-endostatin with DDP/LBP might increase the 0.5–2-year OS rate. But the evidence fails to support that Rh-endostatin plus chemical irritants also does for MPE what it does for non-lung cancer, refractory/recurrent, or drug-resistant patients. Their ADRs and potential TRAEs remain unclear. In addition, whether Rh-endostatin with CBP, NDP, or LBP/BLM improves the clinical response and their treatment thresholds and optimal strategy also remains unclear. All of these questions need further new trials to demonstrate. Finally, these findings provide valuable references for an optimal control strategy based on Rh-endostatin in MPE.

## Data Availability Statement

The original contributions presented in the study are included in the article/**Supplementary Material** further inquiries can be directed to the corresponding authors.

## Author Contributions 

Conception and design by ZX, XX, and X-FC. Development of methodology by ZX, C-QW, and X-FC. Literature search by C-QW and HJ. Article selection by C-QW and MH. Assessment of methodological bias risk by X-RH and QC. Data extraction by X-TZ and T-yF. Statistical analysis by C-QW and X-RH. GRADE assessment by X-FC and C-QW. Preparing the manuscript draft by ZX, XX, and X-FC. Review and revision of the manuscript by XX, X-FC, LZ, JL, and J-HF. Study supervision by ZX. All authors contributed to the article and approved the submitted version.

## Funding

This work was funded by special funds for academic seedlings training and innovation at Zunyi Medical College [Qian Kehe Pingtai Rencai No. (2017) 5733-034], special funds for science and technology research into traditional Chinese and national medicine in Guizhou (No. QZYY 2017-084), and a high-level innovative talent program in Guizhou (No. fzc 120171001).

## Conflict of Interest

The authors declare that the research was conducted in the absence of any commercial or financial relationships that could be construed as a potential conflict of interest.

## Publisher’s Note

All claims expressed in this article are solely those of the authors and do not necessarily represent those of their affiliated organizations, or those of the publisher, the editors and the reviewers. Any product that may be evaluated in this article, or claim that may be made by its manufacturer, is not guaranteed or endorsed by the publisher.

## Abbreviations

ADRs, adverse drug reactions; AST, anticipated survival time; BLM, bleomycin; CBM, China Biological Medicine Database; CBP, carboplatin; CENTRAL, Cochrane Central Register of Controlled Trials; Chi-CTR, Chinese clinical trial registry; CNKI, China National Knowledge Infrastructure Database; CR, complete response; CTCAE, Common Terminology Criteria for Adverse Events standards; DDP, cisplatin; FEM, fixed-effects model; GRADE approach, Grading of Recommendations Assessment, Development and Evaluation approach; HR, hazard ratio; IPC, indwelling pleural catheter; ISI, Web of Science; KPS, Karnofsky Performance Status; LBP, lobaplatin; MPE, malignant pleural effusion; NDP, nedaplatin; NSCLC, non-small-cell lung cancer; NR, no response; OS, overall survival; OR, odds ratio; PD, progressive disease; PFS, progression-free survival; PR, partial response; PRISMA guidelines, Preferred Reporting Items for Systematic reviews and Meta-Analyses guidelines; QOL, quality of life; RCTs, randomized controlled trials; REM, random-effects model; Rh-endostatin, recombinant human endostatin; SD, stable disease; SRs, systematic reviews; TCMIs, traditional Chinese medicine injections; TRAEs, treatment-related adverse events; TRM, treatment-related mortality; VATS, video-assisted thoracic surgery; VIP, Chinese Scientific Journals Full-text Database; WHO-ICTRP, WHO International Clinical Trials Registry Platform; WHO, World Health Organization.
