# Supplementary material for: Intrapleural Administration With Rh-Endostatin and Chemical Irritants in the Control of Malignant Pleural Effusion: A Systematic Review and Meta-Analysis
Source: Front Oncol. 2021 Aug 3;11:649999. doi: 10.3389/fonc.2021.649999 (PMC8369576; doi:10.3389/fonc.2021.649999)
Supplement: Supplementary file 1 [file DataSheet_1.zip › Supplementary Material 2-3.docx]

**Evaluation criteria**

**Supplementary Material S2. Evaluation criteria of clinical responses**

- Complete response (CR) is a response with no replenishment of pleural effusion within four weeks after treatment; or pleural effusion disappeared for more than four weeks.
- Partial response (PR) is a response that does not need additional drainage since, in chest X-ray findings, there is the replenishment of pleural effusion under 50% of pleural effusion at the pre-treatment, but there are no symptoms; or pleural effusion was reduced more than 50% for more than four weeks.
- No response (NR) or stable disease (SD) is a response that needs additional treatment due to the recurrence of pleural effusion within four weeks after treatment; or pleural effusion was reduced less than 50% or increased less than 25%.
- Progressive disease (PD) is a response that pleural effusion increased more than 25% along with other signs of progressive disease.

**Supplementary Material S3. Evidence quality summary model**

**We downgraded the quality according to five domains as following:**

(i) Methodological bias risk (If all trials had high risk, we downgraded the evidence by two grades. Most trials had unclear risk and some trials had high risk, if sensitivity analysis results had poor robustness, we downgraded it by two grades; if good robustness, we downgraded it by one grade. The trials had unclear risk, and without high risk, and we downgraded it by one grade. );

(ii) Statistical heterogeneity (The indicator had statistical heterogeneity, and the results were poor robust);

(iii) Indirectness (the patient, intervention, control, or outcomes did not fit the purposes of this study);

(iv) Imprecision (The number of subjects in each outcome was less than 300);

(v) Publication bias (the indicator had publication bias, and the results were poor robust.

For (ii) to (v), we downgraded it by one grade.
