# Supplementary material for: Intrapleural Administration With Rh-Endostatin and Chemical Irritants in the Control of Malignant Pleural Effusion: A Systematic Review and Meta-Analysis
Source: Front Oncol. 2021 Aug 3;11:649999. doi: 10.3389/fonc.2021.649999 (PMC8369576; doi:10.3389/fonc.2021.649999)
Supplement: Supplementary file 1 [file DataSheet_1.zip › Supplementary Material 4.docx]

**Supplementary Material S4. Retrieval results**

| **Table S1. Identified 24 records from Pubmed** | | |
| --- | --- | --- |
| **Search** | **Query** | **Items found** |
| #18 | ("Pleural Effusion"[Mesh]) OR (Pleural Effusion) OR (Pleural Effusions) OR (Hydrothorax) OR (MPEs) OR (MPE)AND (YH-16) OR (Sulijia) OR (Endostar) OR (Rh-endostatin) OR (rhES) OR (recombinant human endostatin injection) OR (Endostatin) OR (Endostatins) OR ("Endostatins"[Mesh]) | [24](https://pubmed.ncbi.nlm.nih.gov/?term=%28%28Endostar%29+OR+%28%28%28%28%28%28Rh-endostatin%29+OR+%28rhES%29%29+OR+%28recombinant+human+endostatin+injection%29%29+OR+%28Endostatin%29%29+OR+%28Endostatins%29%29+OR+%28%22Endostatins%22%5BMesh%5D%29%29%29+AND+%28%28%28%28%28%28%22Pleural+Effusion%22%5BMesh%5D%29+OR+%28Pleural+Effusion%29%29+OR+%28Pleural+Effusions%29%29+OR+%28Hydrothorax%29%29+OR+%28MPEs%29%29+OR+%28MPE%29%29&sort=) |
| #17 | Search: ("Pleural Effusion"[Mesh]) OR (Pleural Effusion) OR (Pleural Effusions) OR (Hydrothorax) OR (MPEs) OR (MPE) | [36,717](https://pubmed.ncbi.nlm.nih.gov/?term=%28%28%28%28%28%22Pleural+Effusion%22%5BMesh%5D%29+OR+%28Pleural+Effusion%29%29+OR+%28Pleural+Effusions%29%29+OR+%28Hydrothorax%29%29+OR+%28MPEs%29%29+OR+%28MPE%29&sort=) |
| #16 | Search: MPE | [2,202](https://pubmed.ncbi.nlm.nih.gov/?term=MPE&sort=relevance) |
| #15 | Search: MPEs | [275](https://pubmed.ncbi.nlm.nih.gov/?term=MPEs&sort=relevance) |
| #14 | Search: Hydrothorax | [2,181](https://pubmed.ncbi.nlm.nih.gov/?term=Hydrothorax&sort=relevance) |
| #13 | Search: Pleural Effusions | [33,579](https://pubmed.ncbi.nlm.nih.gov/?term=Pleural+Effusions&sort=relevance) |
| #12 | Search: Pleural Effusion | [31,053](https://pubmed.ncbi.nlm.nih.gov/?term=+Pleural+Effusion&sort=relevance) |
| #11 | Search: "Pleural Effusion"[Mesh] | [19,701](https://pubmed.ncbi.nlm.nih.gov/?term=%22Pleural+Effusion%22%5BMesh%5D+&sort=relevance) |
| #10 | Search: (YH-16) OR (Sulijia) OR (Endostar) OR (Rh-endostatin) OR (rhES) OR (recombinant human endostatin injection) OR (Endostatin) OR (Endostatins) OR ("Endostatins"[Mesh]) | [2,477](https://pubmed.ncbi.nlm.nih.gov/?term=%28Endostar%29+OR+%28%28%28%28%28%28Rh-endostatin%29+OR+%28rhES%29%29+OR+%28recombinant+human+endostatin+injection%29%29+OR+%28Endostatin%29%29+OR+%28Endostatins%29%29+OR+%28%22Endostatins%22%5BMesh%5D%29%29&sort=) |
| #9 | YH-16 | 14 |
| #8 | Search: Sulijia | 0 |
| #7 | Search: Endostar | [241](https://pubmed.ncbi.nlm.nih.gov/?term=Endostar&sort=relevance) |
| #6 | Search: Rh-endostatin | [67](https://pubmed.ncbi.nlm.nih.gov/?term=Rh-endostatin&sort=relevance) |
| #5 | Search: rhES | [126](https://pubmed.ncbi.nlm.nih.gov/?term=rhES&sort=relevance) |
| #4 | Search: recombinant human endostatin injection | [163](https://pubmed.ncbi.nlm.nih.gov/?term=recombinant+human+endostatin+injection&sort=relevance) |
| #3 | Search: Endostatin | [2,336](https://pubmed.ncbi.nlm.nih.gov/?term=Endostatin&sort=relevance) |
| #2 | Search: Endostatins | [2,336](https://pubmed.ncbi.nlm.nih.gov/?term=Endostatins&sort=relevance) |
| #1 | Search: "Endostatins"[Mesh] | [1,682](https://pubmed.ncbi.nlm.nih.gov/?term=%22Endostatins%22%5BMesh%5D+&sort=relevance) |

| **Table S2. Identified 58 records from Embase** | | |
| --- | --- | --- |
| **Search** | **Query** | **Items found** |
| #16 | #9 AND #15 | [58](https://www-embase-com.ezproxy.cul.columbia.edu/) |
| #15 | #10 OR #11 OR #12 OR #13 OR #14 | [76,853](https://www-embase-com.ezproxy.cul.columbia.edu/) |
| #14 | mpe | [3,413](https://www-embase-com.ezproxy.cul.columbia.edu/) |
| #13 | mpes | [406](https://www-embase-com.ezproxy.cul.columbia.edu/) |
| #12 | 'hydrothorax'/exp OR hydrothorax | [4,831](https://www-embase-com.ezproxy.cul.columbia.edu/) |
| #11 | 'pleural effusions'/exp OR 'pleural effusions' OR (pleural AND effusions) | [14,683](https://www-embase-com.ezproxy.cul.columbia.edu/) |
| #10 | pleural effusion'/exp OR 'pleural effusion' OR (pleural AND ('effusion'/exp OR effusion)) | [69,611](https://www-embase-com.ezproxy.cul.columbia.edu/) |
| #9 | #1 OR #2 OR #3 OR #4 OR #5 OR #6 OR #7 OR #8 | [5,064](https://www-embase-com.ezproxy.cul.columbia.edu/) |
| #8 | 'yh 16'/exp OR 'yh 16' | 791 |
| #7 | Sulijia | 0 |
| #6 | 'endostar'/exp OR endostar | [843](https://www-embase-com.ezproxy.cul.columbia.edu/) |
| #5 | 'rh endostatin' | [132](https://www-embase-com.ezproxy.cul.columbia.edu/) |
| #4 | rhes | [177](https://www-embase-com.ezproxy.cul.columbia.edu/) |
| #3 | recombinant human endostatin injection' OR (('recombinant'/exp OR recombinant) AND ('human'/exp OR human) AND ('endostatin'/exp OR endostatin) AND ('injection'/exp OR injection)) | [148](https://www-embase-com.ezproxy.cul.columbia.edu/) |
| #2 | endostatin'/exp OR endostatin | [4,916](https://www-embase-com.ezproxy.cul.columbia.edu/) |
| #1 | 'endostatins'/exp OR endostatins | [4,088](https://www-embase-com.ezproxy.cul.columbia.edu/) |

| **Table S3. Identified 222 records from China Biological Medicine Database** | | |
| --- | --- | --- |
| **Search** | **Query** | **Items found** |
| #16 | (("胸膜腔积水"[全部字段:智能]) OR ("胸膜腔积液"[全部字段:智能]) OR ("MPE"[全部字段:智能]) OR ("胸腔积水"[全部字段:智能]) OR ("胸水"[全部字段:智能]) OR ("胸腔积液"[全部字段:智能])) AND (("重组人血管内皮抑制素注射液"[全部字段:智能]) OR ("重组人血管内皮抑制素"[全部字段:智能]) OR ("血管内皮抑制素"[全部字段:智能]) OR ("恩度"[全部字段:智能]) OR ("Endostar"[全部字段:智能]) OR ("苏粒佳"[全部字段:智能]) OR ("YH-16"[全部字段:智能]))  ("Pleural effusion" [all fields: smart] OR "Pleural effusion" [all fields: smart] OR "MPE" [all fields: smart] OR "Pleural effusion" [all fields: smart] OR "Pleural effusion" [all fields: smart] OR "Pleural effusion" [all fields: smart]) AND ("recombinant human endostatin injection "[all fields: smart] OR " recombinant human endostatin "[all fields: smart] OR "endostatin "[all fields: smart] OR "Endu"[all fields: smart] OR "Endostar"[all fields: smart] OR "Sulijia"[all fields: smart] OR "YH-16"[all fields: smart]) | 222 |
| #15 | ("胸膜腔积水"[全部字段:智能]) OR ("胸膜腔积液"[全部字段:智能]) OR ("MPE"[全部字段:智能]) OR ("胸腔积水"[全部字段:智能]) OR ("胸水"[全部字段:智能]) OR ("胸腔积液"[全部字段:智能])  "Pleural effusion" [all fields: smart] OR "Pleural effusion" [all fields: smart] OR "MPE" [all fields: smart] OR "Pleural effusion" [all fields: smart] OR "Pleural effusion" [all fields: smart] OR "Pleural effusion" [all fields: smart] | [65,578](javascript:historyLink('(%22%E8%83%B8%E8%86%9C%E8%85%94%E7%A7%AF%E6%B0%B4%22[%E5%85%A8%E9%83%A8%E5%AD%97%E6%AE%B5:%E6%99%BA%E8%83%BD])%20OR%20(%22%E8%83%B8%E8%86%9C%E8%85%94%E7%A7%AF%E6%B6%B2%22[%E5%85%A8%E9%83%A8%E5%AD%97%E6%AE%B5:%E6%99%BA%E8%83%BD])%20OR%20(%22MPE%22[%E5%85%A8%E9%83%A8%E5%AD%97%E6%AE%B5:%E6%99%BA%E8%83%BD])%20OR%20(%22%E8%83%B8%E8%85%94%E7%A7%AF%E6%B0%B4%22[%E5%85%A8%E9%83%A8%E5%AD%97%E6%AE%B5:%E6%99%BA%E8%83%BD])%20OR%20(%22%E8%83%B8%E6%B0%B4%22[%E5%85%A8%E9%83%A8%E5%AD%97%E6%AE%B5:%E6%99%BA%E8%83%BD])%20OR%20(%22%E8%83%B8%E8%85%94%E7%A7%AF%E6%B6%B2%22[%E5%85%A8%E9%83%A8%E5%AD%97%E6%AE%B5:%E6%99%BA%E8%83%BD])')) |
| #14 | "胸膜腔积水"[全部字段:智能]  "Pleural effusion" [all fields: smart] | [0](javascript:historyLink('%22%E8%83%B8%E8%86%9C%E8%85%94%E7%A7%AF%E6%B0%B4%22[%E5%85%A8%E9%83%A8%E5%AD%97%E6%AE%B5:%E6%99%BA%E8%83%BD]')) |
| #13 | "胸膜腔积液"[全部字段:智能]  "Pleural effusion" [all fields: smart] | [289](javascript:historyLink('%22%E8%83%B8%E8%86%9C%E8%85%94%E7%A7%AF%E6%B6%B2%22[%E5%85%A8%E9%83%A8%E5%AD%97%E6%AE%B5:%E6%99%BA%E8%83%BD]')) |
| #12 | "MPE"[全部字段:智能]  "MPE" [all fields: smart] | [2,510](javascript:historyLink('%22MPE%22[%E5%85%A8%E9%83%A8%E5%AD%97%E6%AE%B5:%E6%99%BA%E8%83%BD]')) |
| #11 | "胸腔积水"[全部字段:智能]  "Pleural effusion" [all fields: smart] | [54](javascript:historyLink('%22%E8%83%B8%E8%85%94%E7%A7%AF%E6%B0%B4%22[%E5%85%A8%E9%83%A8%E5%AD%97%E6%AE%B5:%E6%99%BA%E8%83%BD]')) |
| #10 | "胸水"[全部字段:智能]  "Pleural effusion" [all fields: smart] | [11,232](javascript:historyLink('%22%E8%83%B8%E6%B0%B4%22[%E5%85%A8%E9%83%A8%E5%AD%97%E6%AE%B5:%E6%99%BA%E8%83%BD]')) |
| #9 | "胸腔积液"[全部字段:智能]  "Pleural effusion" [all fields: smart] | [59,093](javascript:historyLink('%22%E8%83%B8%E8%85%94%E7%A7%AF%E6%B6%B2%22[%E5%85%A8%E9%83%A8%E5%AD%97%E6%AE%B5:%E6%99%BA%E8%83%BD]')) |
| #8 | ("重组人血管内皮抑制素注射液"[全部字段:智能]) OR ("重组人血管内皮抑制素"[全部字段:智能]) OR ("血管内皮抑制素"[全部字段:智能]) OR ("恩度"[全部字段:智能]) OR ("Endostar"[全部字段:智能]) OR ("苏粒佳"[全部字段:智能]) OR ("YH-16"[全部字段:智能])  "recombinant human endostatin injection "[all fields: smart] OR " recombinant human endostatin "[all fields: smart] OR "endostatin "[all fields: smart] OR "Endu"[all fields: smart] OR "Endostar"[all fields: smart] OR "Sulijia"[all fields: smart] OR "YH-16"[all fields: smart] | [2,014](javascript:historyLink('(%22%E9%87%8D%E7%BB%84%E4%BA%BA%E8%A1%80%E7%AE%A1%E5%86%85%E7%9A%AE%E6%8A%91%E5%88%B6%E7%B4%A0%E6%B3%A8%E5%B0%84%E6%B6%B2%22[%E5%85%A8%E9%83%A8%E5%AD%97%E6%AE%B5:%E6%99%BA%E8%83%BD])%20OR%20(%22%E9%87%8D%E7%BB%84%E4%BA%BA%E8%A1%80%E7%AE%A1%E5%86%85%E7%9A%AE%E6%8A%91%E5%88%B6%E7%B4%A0%22[%E5%85%A8%E9%83%A8%E5%AD%97%E6%AE%B5:%E6%99%BA%E8%83%BD])%20OR%20(%22%E8%A1%80%E7%AE%A1%E5%86%85%E7%9A%AE%E6%8A%91%E5%88%B6%E7%B4%A0%22[%E5%85%A8%E9%83%A8%E5%AD%97%E6%AE%B5:%E6%99%BA%E8%83%BD])%20OR%20(%22%E6%81%A9%E5%BA%A6%22[%E5%85%A8%E9%83%A8%E5%AD%97%E6%AE%B5:%E6%99%BA%E8%83%BD])%20OR%20(%22Endostar%22[%E5%85%A8%E9%83%A8%E5%AD%97%E6%AE%B5:%E6%99%BA%E8%83%BD])%20OR%20(%22%E8%8B%8F%E7%B2%92%E4%BD%B3%22[%E5%85%A8%E9%83%A8%E5%AD%97%E6%AE%B5:%E6%99%BA%E8%83%BD])%20OR%20(%22YH-16%22[%E5%85%A8%E9%83%A8%E5%AD%97%E6%AE%B5:%E6%99%BA%E8%83%BD])')) |
| #7 | "重组人血管内皮抑制素注射液"[全部字段:智能]  " recombinant human endostatin injection "[all fields: smart] | [165](javascript:historyLink('%22%E9%87%8D%E7%BB%84%E4%BA%BA%E8%A1%80%E7%AE%A1%E5%86%85%E7%9A%AE%E6%8A%91%E5%88%B6%E7%B4%A0%E6%B3%A8%E5%B0%84%E6%B6%B2%22[%E5%85%A8%E9%83%A8%E5%AD%97%E6%AE%B5:%E6%99%BA%E8%83%BD]')) |
| #6 | "重组人血管内皮抑制素"[全部字段:智能]  " recombinant human endostatin "[all fields: smart] | [822](javascript:historyLink('%22%E9%87%8D%E7%BB%84%E4%BA%BA%E8%A1%80%E7%AE%A1%E5%86%85%E7%9A%AE%E6%8A%91%E5%88%B6%E7%B4%A0%22[%E5%85%A8%E9%83%A8%E5%AD%97%E6%AE%B5:%E6%99%BA%E8%83%BD]')) |
| #5 | "血管内皮抑制素"[全部字段:智能]  "endostatin "[all fields: smart] | [880](javascript:historyLink('%22%E8%A1%80%E7%AE%A1%E5%86%85%E7%9A%AE%E6%8A%91%E5%88%B6%E7%B4%A0%22[%E5%85%A8%E9%83%A8%E5%AD%97%E6%AE%B5:%E6%99%BA%E8%83%BD]')) |
| #4 | "恩度"[全部字段:智能]  "Endu"[all fields: smart] | [1,275](javascript:historyLink('%22%E6%81%A9%E5%BA%A6%22[%E5%85%A8%E9%83%A8%E5%AD%97%E6%AE%B5:%E6%99%BA%E8%83%BD]')) |
| #3 | "Endostar"[全部字段:智能]  "Endostar"[all fields: smart] | [764](javascript:historyLink('%22Endostar%22[%E5%85%A8%E9%83%A8%E5%AD%97%E6%AE%B5:%E6%99%BA%E8%83%BD]')) |
| #2 | "苏粒佳"[全部字段:智能]  "Sulijia"[all fields: smart] | [0](javascript:historyLink('%22%E8%8B%8F%E7%B2%92%E4%BD%B3%22[%E5%85%A8%E9%83%A8%E5%AD%97%E6%AE%B5:%E6%99%BA%E8%83%BD]')) |
| #1 | "YH-16"[全部字段:智能]  "YH-16"[all fields: smart] | [143](javascript:historyLink('%22YH-16%22[%E5%85%A8%E9%83%A8%E5%AD%97%E6%AE%B5:%E6%99%BA%E8%83%BD]')) |

| **Table S4.**The included trials from the systematic review and meta-analysis | | | | |
| --- | --- | --- | --- | --- |
| **Authors** | **Title** | **Included trials** | **Trials included in this study** | **Journals** |
| Liang, R.2015 | Intraperitoneal Perfusion Therapy of Endostar Combined with Platinum Chemotherapy for Malignant Serous Effusions: A Meta-analysis. | 25 trials | 18 trials(1-18) | Asian Pac J Cancer Prev **16**(18): 8637-8644. |
| Yang, M. 2015 | Recombinant human endostatin combined with cisplatin perfusion chemotherapy for malignant pleural effusions:a Meta-analysis. | 15 trials | 14 trials (1, 3, 5, 11, 12, 14-16, 18-23) | Chin Clin Oncol **20**(12): 1117-1123. |
| Jiang, J. 2016 | Efficacy and safety of recombinant human Endostatin combined with cisplatin for malignant pleural effusion:a meta-analysis | 17 trials | 14 trials (1-3, 5-8, 11, 12, 14-16, 18-20) | Pract J Cancer **31**(3): 411-416. |
| Liu, Z. 2016 | Efficacy and safety of endostar combined with cisplatin in treatment of non-small cell lung cancer with malignant pleural effusion: A meta-analysis | 10 trials | Nine trials(1, 3, 7, 9, 14, 16, 18-20) | Chin J Evid-based Med **16**(5): 557-563. |
| Sun, Y. 2016 | Endostar combined with cisplatin in the treatment of malignant pleural effusion: A meta-analysis | 19 trials | 18 trials(1-3, 5, 7, 9, 12, 14, 16-20, 22, 24-27) | Mod Bus Trade Ind **37**(33): 166-169. |
| Yang, H. 2016 | Efficacy and safety of combination of recombinant human Endostatin and cisplatin in treatment of malignant pleural effusion: A meta-analysis. | 20 trials | 17 trials(2, 3, 5-7, 9, 11, 12, 14-16, 18-20, 22, 23, 28) | J Chin Oncol **22**(5): 410-416. |
| Biaoxue, R. 2016 | Thoracic perfusion of recombinant human endostatin (Endostar) combined with chemotherapeutic agents versus chemotherapeutic agents alone for treating malignant pleural effusions: a systematic evaluation and meta-analysis. | 13 trials | Eight trials (3, 5, 10, 15, 18, 20, 29, 30) | BMC Cancer **16**(1): 888. |
| Liu, M. 2017 | Efficacy and safety of injecting endostar combined with platinum complexes into pleural cavity for treatment of malignant pleural effusion: a meta-analysis review. | Nine trials | Eight trials(1, 5, 7, 10, 12-14, 16) | J Chin Physician **19**(2): 257-262. |
| Lv, C. 2017 | Short-term therapeutic effect of recombinant human Endostatin combined with cis-platinum on malignant pleural effusions: A meta-analysis. | 20 trials | 15 trials(2, 3, 5-9, 11, 12, 14-16, 18, 20, 24) | Herald Med **36**(05): 558-563 |
| Zhao, Z. 2018 | A meta-analysis of the efficacy and safety of recombinant human vascular Endostatin combined with cisplatin in the treatment of non-small cell lung cancer with malignant pleural effusion. | 15 trials | 12 trials (1, 3, 7, 9, 14, 16, 18-20, 31, 32) | Asian Case Rep Oncol **7**(03): 17-25. |
| Zhou, Y. 2018 | Intrapleural injection of Endostar combined with cisplatin for lung cancer with malignant pleural effusion:a meta-analysis. | 25 trials | 20 trials(1, 3, 7, 9, 14, 16, 18-20, 26, 27, 31-39) | J Pract Oncol **33**(6): 553-559. |
| In all, we included 39 trials (1-39) after evaluating previous 11 SRs/meta analyses | | | | |

**Included trials**

1. D. Huang: Clinical observation of Endostar combined with cisplatin in the treatment of malignant pleural effusion. *J Clin Med Pract*, 14(13), 63-64 (2010) doi:10.3969/j.issn.1672-2353.2010.13.023

2. J. Li, K. Gong, Z. Zhu, Q. Yang and M. Ha: Recombinant human endostatin combined with cisplatin for malignant pleural effusion:A clinical study. *Chin Pharm*, 21(14), 1308-1310 (2010)

3. W. Li: Clinical study of endothoracic perfusion in the treatment of malignant pleural effusion. *J Med Forum*, 32(7), 170-171 (2011)

4. Z. Liu, E. Cui, B. Wang and S. Zhang: Clinical study of recombinant human endostatin combined with carboplatin in the treatment of malignant pleural effusion. *Zhejiang JITCWM*, 21(11), 784-785 (2011) doi:10.3969/j.issn.1005-4561.2011.11.017

5. L. Mao, G. Liao, H. Wang, P. Liu and G. Xie: Observation of clinical efficacy of endostar combined with DDP on malignant bloody pleural effusion. *Med J NDFSC*, 21(7), 723-725 (2011) doi:10.3969/j.issn.1004-0188.2011.07.008

6. B. Jiang: Recombinant human endostatin combined with cisplatin in the treatment of malignant pleural effusion: A Clinical Study. In: *Internal medicine*. Qinghai University, Xiling (2012)

7. X. Liu and L. Wang: Clinical observation of intracavitary chemotherapy of Endostar combined with chemotherapy in the treatment of non-small cell lung cancer with malignant pleural effusion. *J Basic Clinl Oncol*, 25(3), 233-235 (2012) doi:10.3969/j.issn.1673-5412.2012.03.016

8. H. Miao and F. Kong: Radiofrequency hyperthermia combined with Endostar and cisplatin in the treatment of malignant pleural effusion. *Chin Commun doctors*, 14(22), 86-87 (2012) doi:10.3969/j.issn.1007-614x.2012.22.082

9. Q. Shen, A. Gu, J. Wu, F. Jin, J. Zhu, X. Yao and X. Huang: Therapeutic observation of endostar combined with cisdiammi dichloride platinum on non-small cell lung cancer with malignant pleural effusion. *J Clin Med Pract*, 16(5), 29-31 (2012) doi:10.3969/j.issn.1672-2353.2012.05.010

10. Q. Yao, Q. Lin, S. Liu, D. Shen, F. Lin and W. Mao: Efficacy of intrapleural infusion of Endostar combined with nedaplatin in the treatment of malignant pleural effusion. *J Basic Clinl Oncol*, 25(6), 492-494 (2012)

11. Z. Han, F. Wang and J. Liu: The effect of rhendostatin combined with dichlorodiamineplatinum on patients with malignant pleural effusion. *Chin J Clin Pharmacol*, 29(8), 587-589 (2013) doi:10.13699/j.cnki.1001-6821.2013.08.013

12. L. Kang, L. Gao, J. Cao, Z. Fu, H. Xu and L. Zheng: Clinical observation on pleural perfusion of recombinant human endostain combined with cisplatin on malignant pleural effusion. *Clin Focus*, 28(12), 1371-1373 (2013) doi:10.3969/j.issn.1004-583X.2013.12.016

13. K. Yang: Clinical study of recombinant human endostatin combined with nadaplatin for treatment of malignant pleural effusion. *Acta Acad Med Xuzhou* 33(12), 891-893 (2013)

14. Y. Yang, R. Lin and G. Cao: Short-term and long-term efficacy of Endostar combined with Cis-diaminedichloroplatinum in treating malignant pleural effusion of non-small cell lung cancer. *China Pharmaceuticals*, 22(19), 21-22 (2013) doi:10.3969/j.issn.1006-4931.2013.19.011

15. Q. Zheng, W. Hu, X. Liao, X. Zheng, Z. Zhou, S. Wu, H. He and H. Hu: A comparision between intrapleural injection of cisplatin combined with Endostar and cisplatin alone in the treatment for malignant pleural effusion. *J Chin Oncol* 19(5), 386-389 (2013)

16. L. Huang: Clinical study of Endostar combined with cisplatin in the treatment of malignant pleural effusion of non-small cell lung cancer. *Jilin Med J*, 35(19), 4308-4309 (2014)

17. H. Lu: Clinical observation of recombinant human endostatin combined with chemotherapy in the treatment of pleural effusion. *Yiayao Qianyan*, 4(14), 396-396 (2014) doi:10.3969/j.issn.2095-1752.2014.14.433

18. G. Yue, Y. Bo, H. Ma, N. Li, W. Bing, L. Shi, S. Xing, G. Shen and R. Li: Analysis of efficacy and security cisplatin Endostar in the treatment of non-small cell lung cancer bloody pleural effusion. *Mod Diag & Treat*, 25(11), 2478-2480 (2014) doi:10.3969/j.issn.1001-8174.2014.11.049

19. J. Chen, S. Gou and W. Luan: Study on the efficacy of Endostar combined with cisplatin in treatment of non-small cell lung cancer with malignant pleural effusion and influence on tumor markers VEGF and HIF-1α. *J Clin Exp Med* 13(21), 1778-1780 (2014) doi:10.3969/j.issn.1671-4695.2014.21.012

20. J. Tu, S. Huang and M. Wang: Clinical efficacy of pleural perfusion with recombinant human Endostatin combined with cisdiammi dichloride platinum for advanced non-small cell lung cancer patients with malignant pleural effusion. *Pract J Cancer*, 29(12), 1592-1594 (2014) doi:10.3969/j.issn.1001-5930.2014.12.026

21. X. Wang and C. Chen: Study of new recombinant Endostatin combined with cisplatin injection for treatment of malignant pleural effusion. *Mod Prev Med*, 39(1), 200-201 (2012)

22. L. He, Y. Shi and G. Hua: Recombinant human endostatin combined with cisplatin in the treatment of malignant pleural effusion: A Clinical study of 32 cases. *China Pharmaceuticals*, 22(12), 49-50 (2013) doi:10.3969/j.issn.1006-4931.2013.12.033

23. Z. Pang and X. Sun: The clinical observation and safety of intrapleural injection of endostar combined with cisplatin in treatment of malignant pleural effusion. *China Med Pharm*, 5(4), 176-178 (2015)

24. X. Hu, H. Wang, C. Zhang, P. Liu, Y. Wang, J. Li and Y. Shi: Clinical study on intra-thoracic chemotherapy with recombinant human endostain combined with cisplatin in treatment of patients with malignant pleural effusion. *Clin Med J*, 13(3), 23-27 (2015) doi:10.3969/j.issn.1672-3384.2015.03.006

25. W. Zhao, C. Ma, W. Zhao, L. Zhang, S. Wang and H. Wei: Clinical study of endostar on malignant cavity effusion. *J Taishan Med Coll*, 36(10), 1089-1092 (2015) doi:10.3969/j.issn.1004-7115.2015.10.003

26. Y. Chang: Clinical analysis of cisplatin combined with Endostar in the treatment of malignant pleural effusion of lung cancer. *Chin J Mod Drug Appl*, 10(10), 155-156 (2016) doi:10.14164/j.cnki.cn11-5581/r.2016.10.115

27. R. Chen, C. Zhang, H. Wu and S. Yang: Clinical effect of pleural perfusion of human recombinant Endostatin injection combined with cisplatin injection on advanced non-small cell lung cancer complicated with malignant pleural effusion. *PJCCPVD*, 24(5), 118-120 (2016) doi:10.3969/j.issn.1008-5971.2016.05.033

28. M. Dong: Comparison of combination of Endostar and cisplatin on the curative effect and immune function in malignant pleural effusion patients. In: *Medical Oncology*. Nanchang University, Nanchang (2015) doi:10.7666/d.D692128

29. G. Y. Li, J. D. Zhao, Y. Y. Li, J. Di, J. Jiang and J. H. Zhao: Application of endostar combined with bleomycin in the treatment of malignant pleural effusion. *Chin J New Drugs* 20(16), 1544-1547 (2011)

30. J. Xu, D. Qi, X. Li and R. Wang: Efficacy of recombinant human endostatin (Endostar) combined with chemotherapy for malignant pleural effusion in non-small cell lung cancer patients. *Chin J Clin Oncol*, 41(24), 1573-1576 (2014) doi:10.3969/j.issn.1000-8179.20141512

31. M. Qin: Clinical study of cisplatin combined with Endostar in the treatment of malignant pleural effusion of advanced non-small cell lung cancer. *China Prac Med*, 11(26), 228-229 (2016) doi:10.14163/j.cnki.11-5547/r.2016.26.147

32. W. Zheng, J. Kang, J. Wen and J. Wang: Influence of recombinant human endostatin combined with cisplatinum on VEGF, EGFR and tumor markers in patients with malignant pleural effusion. *J Clin Med Pract*, 20(23), 32-35 (2016) doi:10.7619/jcmp.201623010

33. F. Chen, Q. Li, G. Jin and H. Zhang: Efficacy of recombinant human endostatin combined with cisplatin in the treatment of malignant pleural effusion of non-small cell lung cancer. *Chin J Oncol Prev Treat*, 8(4), 246-249 (2016) doi:10.3969/j.issn.1674-5671.2016.04.10

34. J. He, J. Hou, M. Zhai and X. Zheng: Evaluation of curative effect of endostar combined with cisplatin intrapleural administration in treatment of malignant pleural effusion induced by non-small cell lung cancer. *Int J Resp*, 36(15), 1127-1130 (2016) doi:10.3760/cma.j.issn.1673-436X.2016.15.002

35. J. Lu, Q. Xie, Q. Chen, W. Sun, A. Zhong, Q. Shi, S. Liao, H. Zhang and J. Zhu: Clinical study of intrapleural injection of recombinant human endostatin combined with cisplatin in the treat-ment of lung adenocarcinoma with malignant pleural effusion. *J Clin Pulm Med*, 21(9), 1664-1666,1667 (2016) doi:10.3969/j.issn.1009-6663.2016.09.032

36. J. Zhou and Y. Du: Effect of recombinant human endostatin combined with cisplatin on inflammatory factors and immune function in elderly patients with malignant pleural effusion. *Chin J Med*, 51(4), 95-97 (2016) doi:10.3969/j.issn.1008-1070.2016.04.029

37. X. Che: Study of endostain combined with cisplation in malignant pleural effusion of lung cancer. *Chin J Biochem Pharm*, 37(2), 157-161 (2017) doi:10.3969/j.isn.1005-1678.2017.02.047

38. X. Lu and T. Zhang: Clinical efficacy of pleural perfusion with recombinant human endostatin and cisplatin in advanced non-small cell lung cancer patients with malignant pleural effusion. *Jiangsu Med J*, 43(14), 1023-1025 (2017) doi:10.19460/j.cnki.0253-3685.2017.14.013

39. Q. Zhao, X. Ouyang and K. Li: Observe the curative effect in the treatment of malignant pleural effusion intrapleural injection of Endostar. *J Clin Med Lit*, 4(1), 14-15 (2017) doi:10.3877/cma.j.issn.2095-8242.2017.01.009
