# Supplementary material for: Intrapleural Administration With Rh-Endostatin and Chemical Irritants in the Control of Malignant Pleural Effusion: A Systematic Review and Meta-Analysis
Source: Front Oncol. 2021 Aug 3;11:649999. doi: 10.3389/fonc.2021.649999 (PMC8369576; doi:10.3389/fonc.2021.649999)
Supplement: Supplementary file 1 [file DataSheet_1.zip › Supplementary Material 5.docx]

**Supplementary Material S5. Meta-analysis results of ADRs and TRAEs (Figures S1-11)**

**
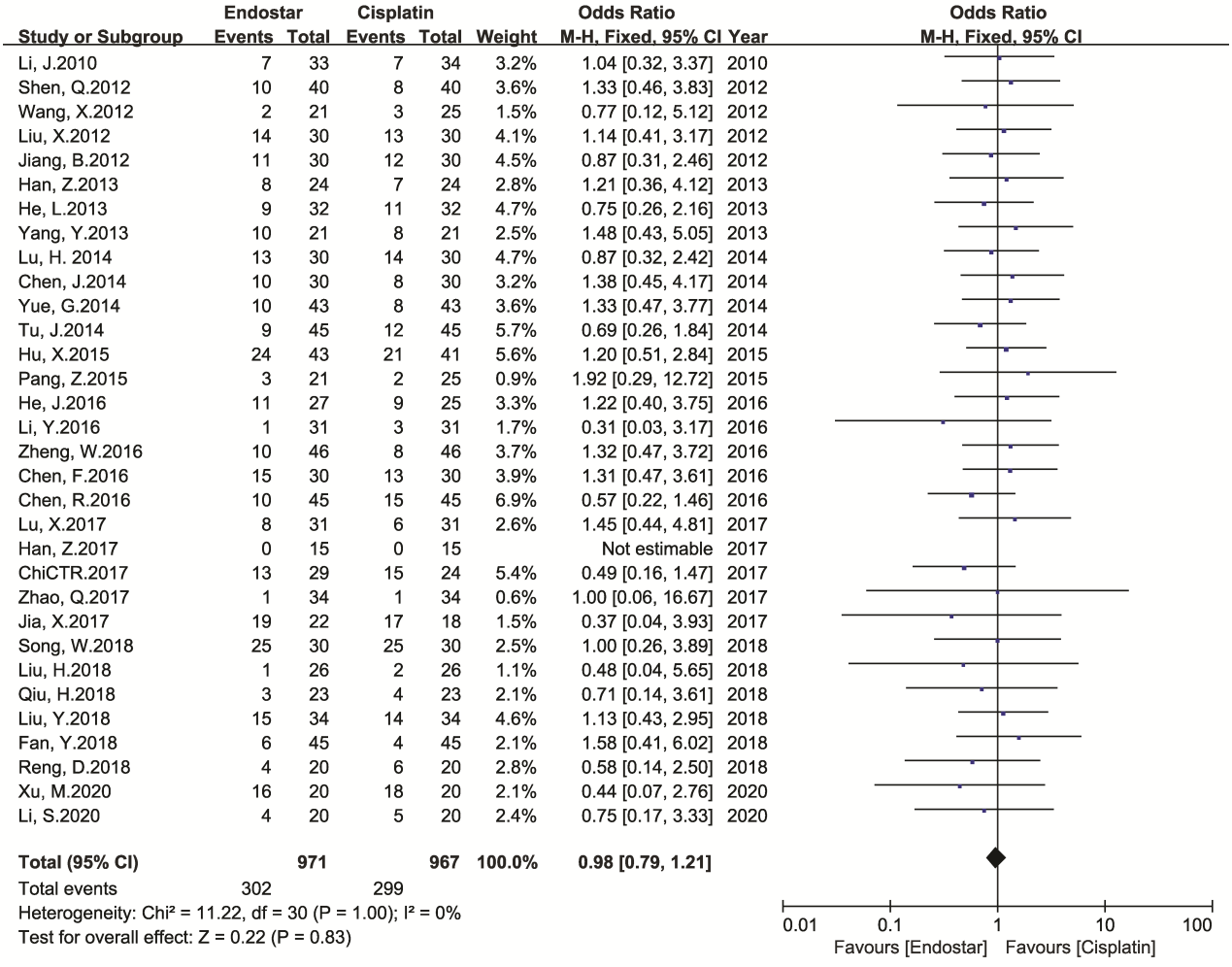
Figure S1. The analysis of neutropenia between the two groups**

**
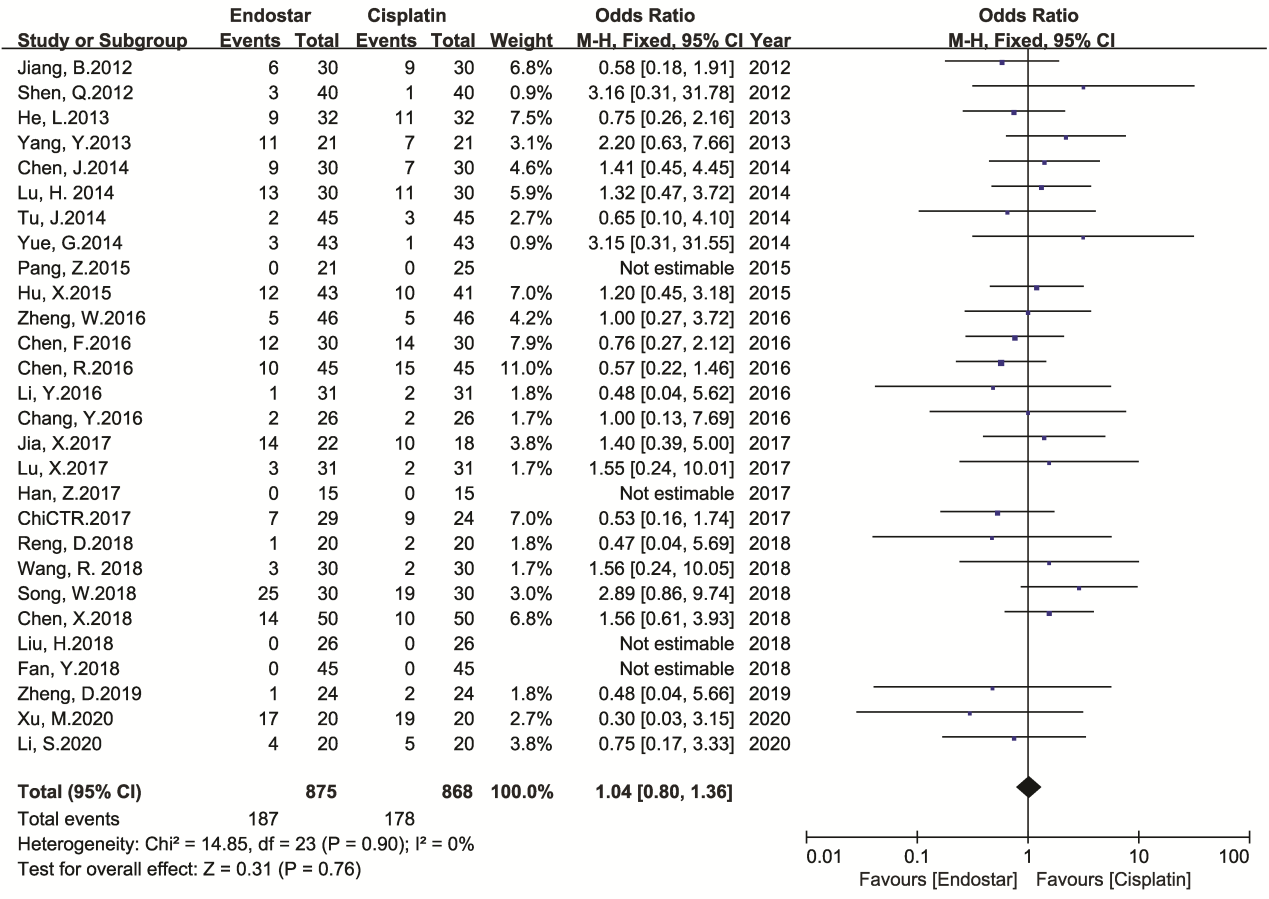
Figure S2. The analysis of thrombocytopenia between the two groups**

**
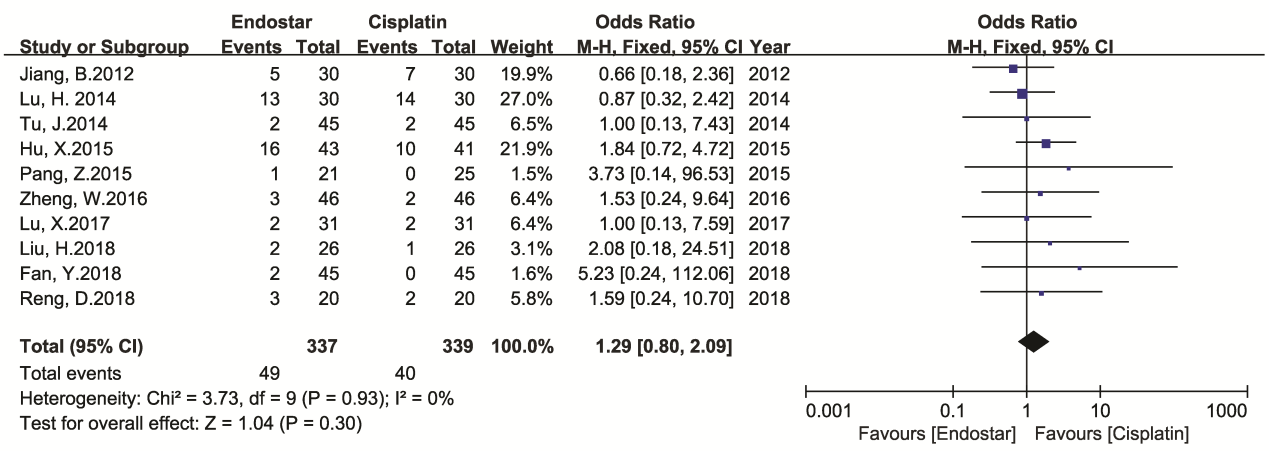
Figure S3. The analysis of anemia between the two groups**

**
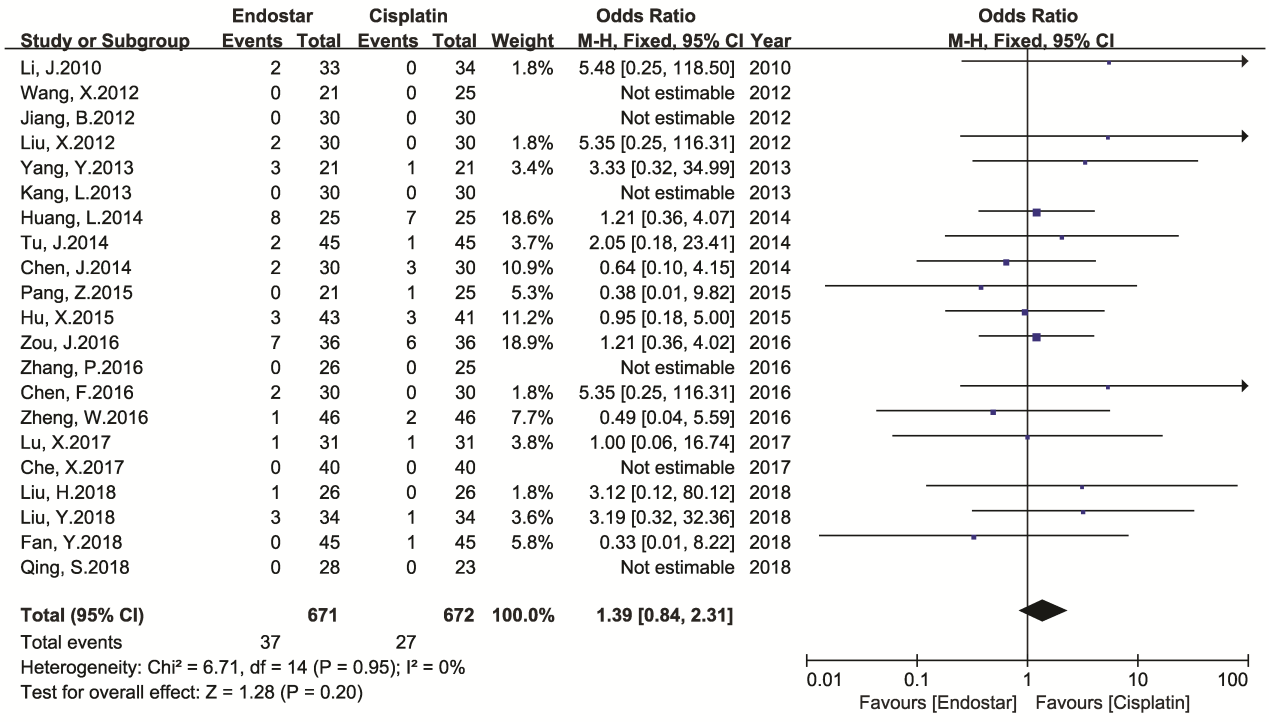
Figure S4. The analysis of cardiotoxicity between the two groups**

**
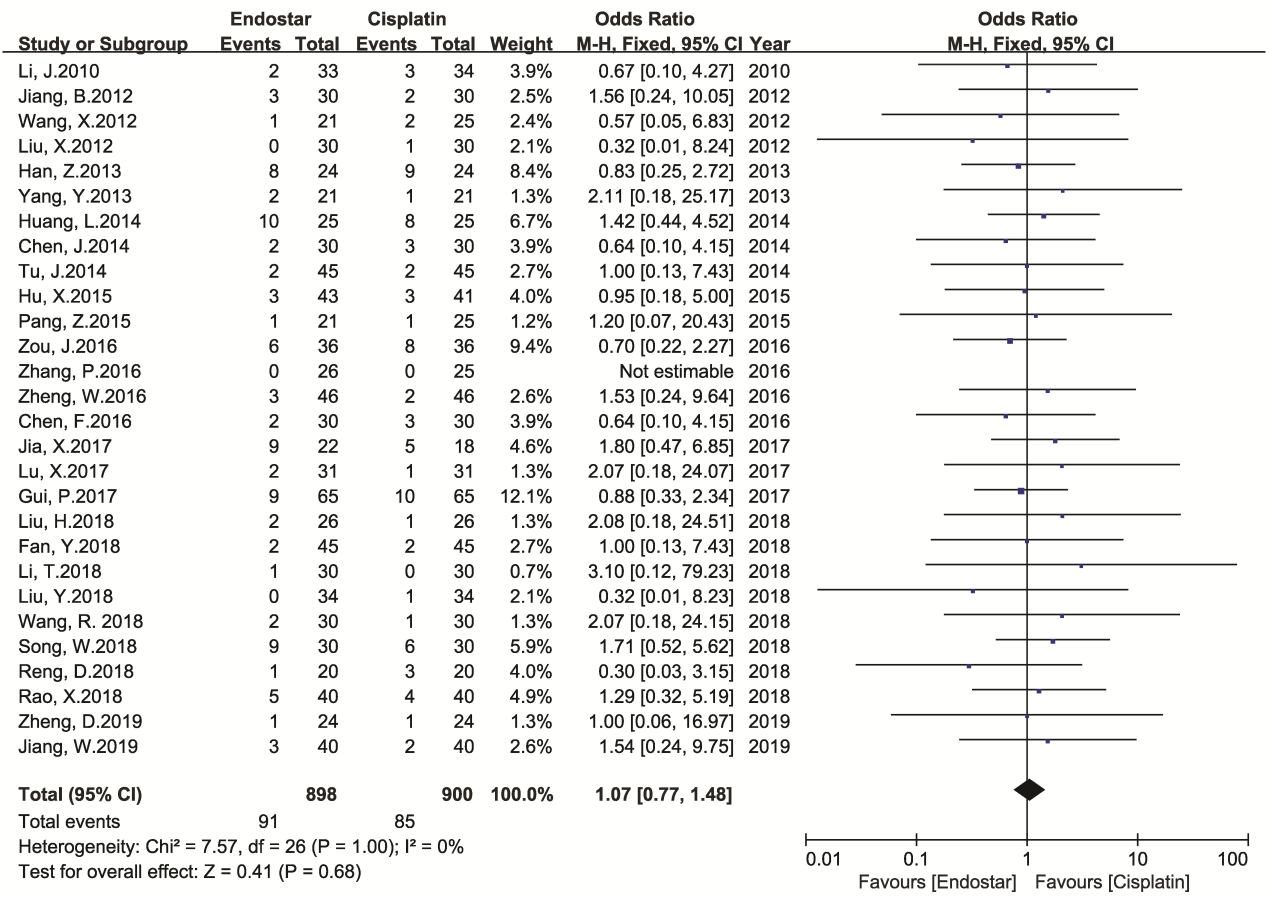
Figure S5. The analysis of hepatotoxicity between two groups**

**
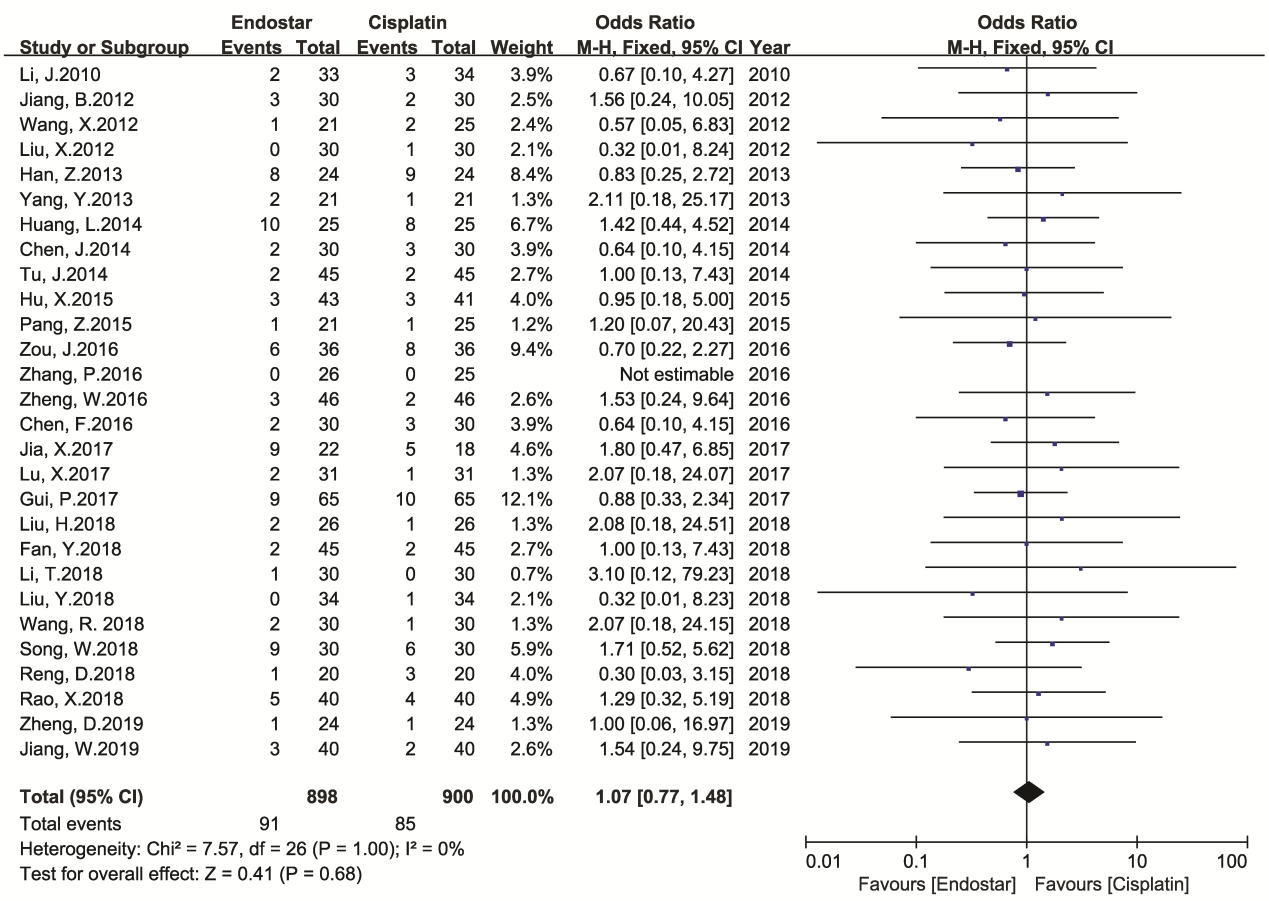
Figure S6. The analysis of nephrotoxicity between the two groups**

**
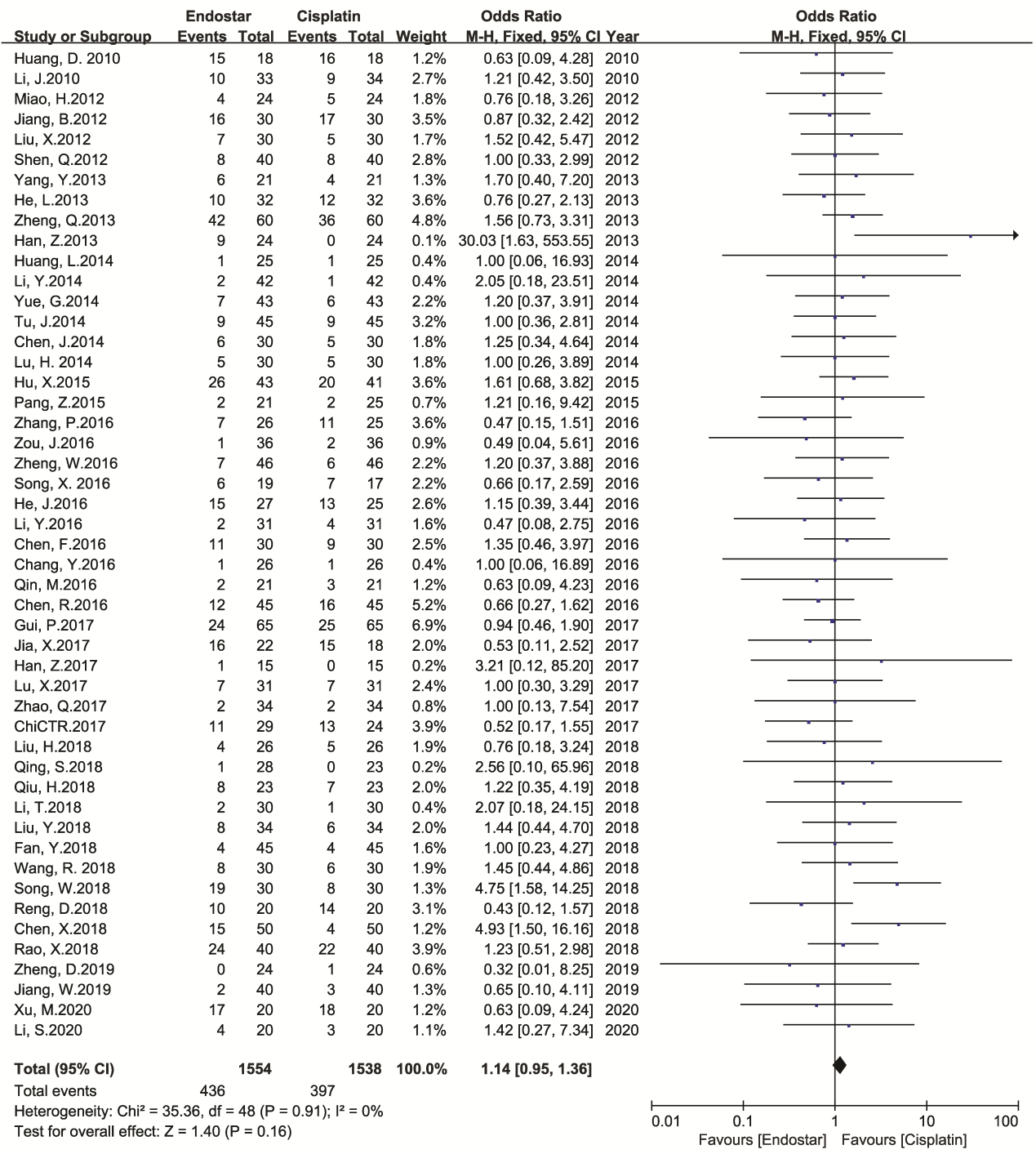
Figure S7. The analysis of nausea and vomiting between the two groups**

**
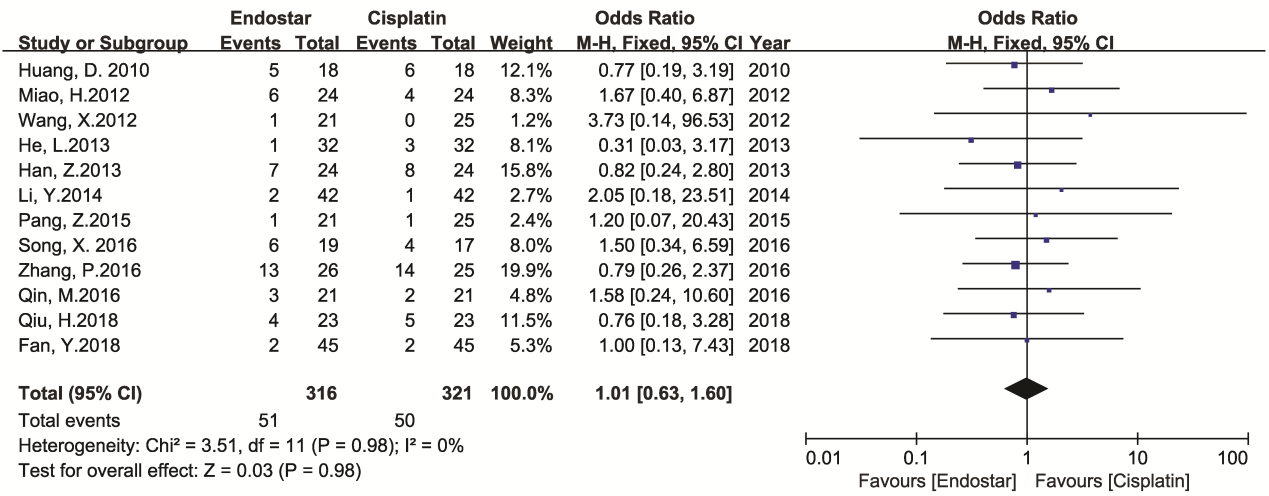
Figure S8. The analysis of chest pain between two groups**

**
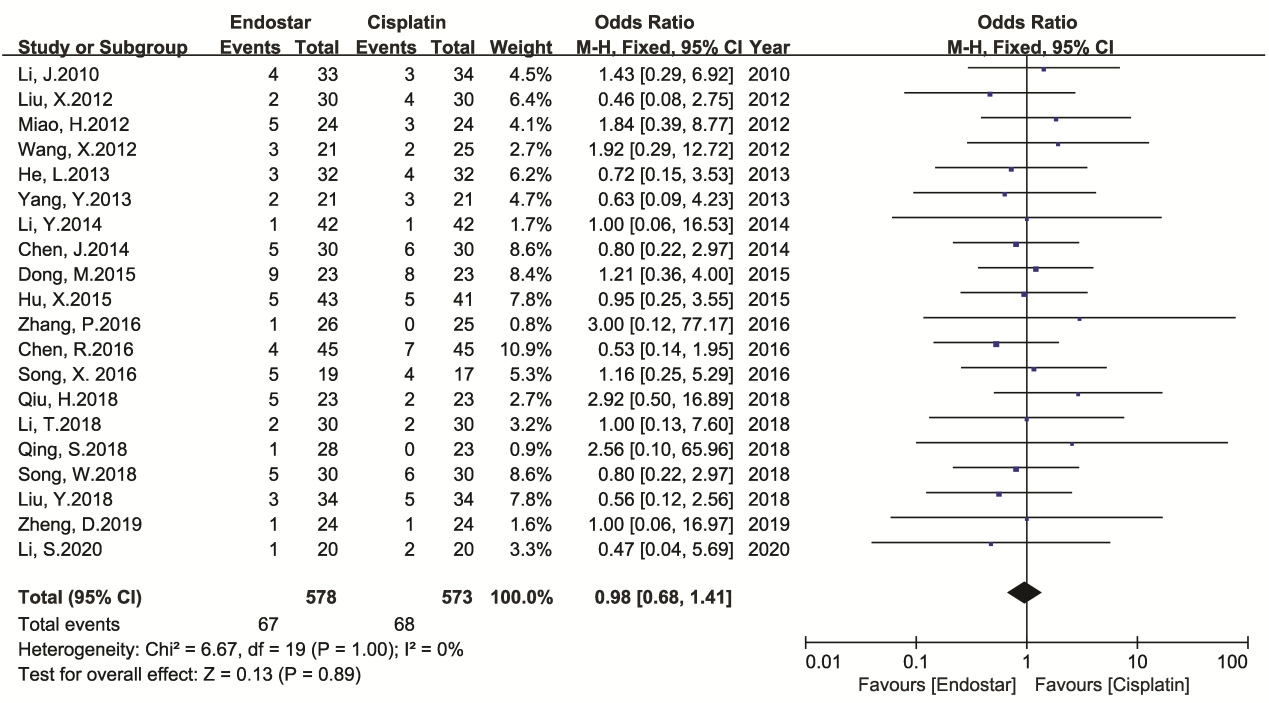
Figure S9. The analysis of fever between two groups**

**
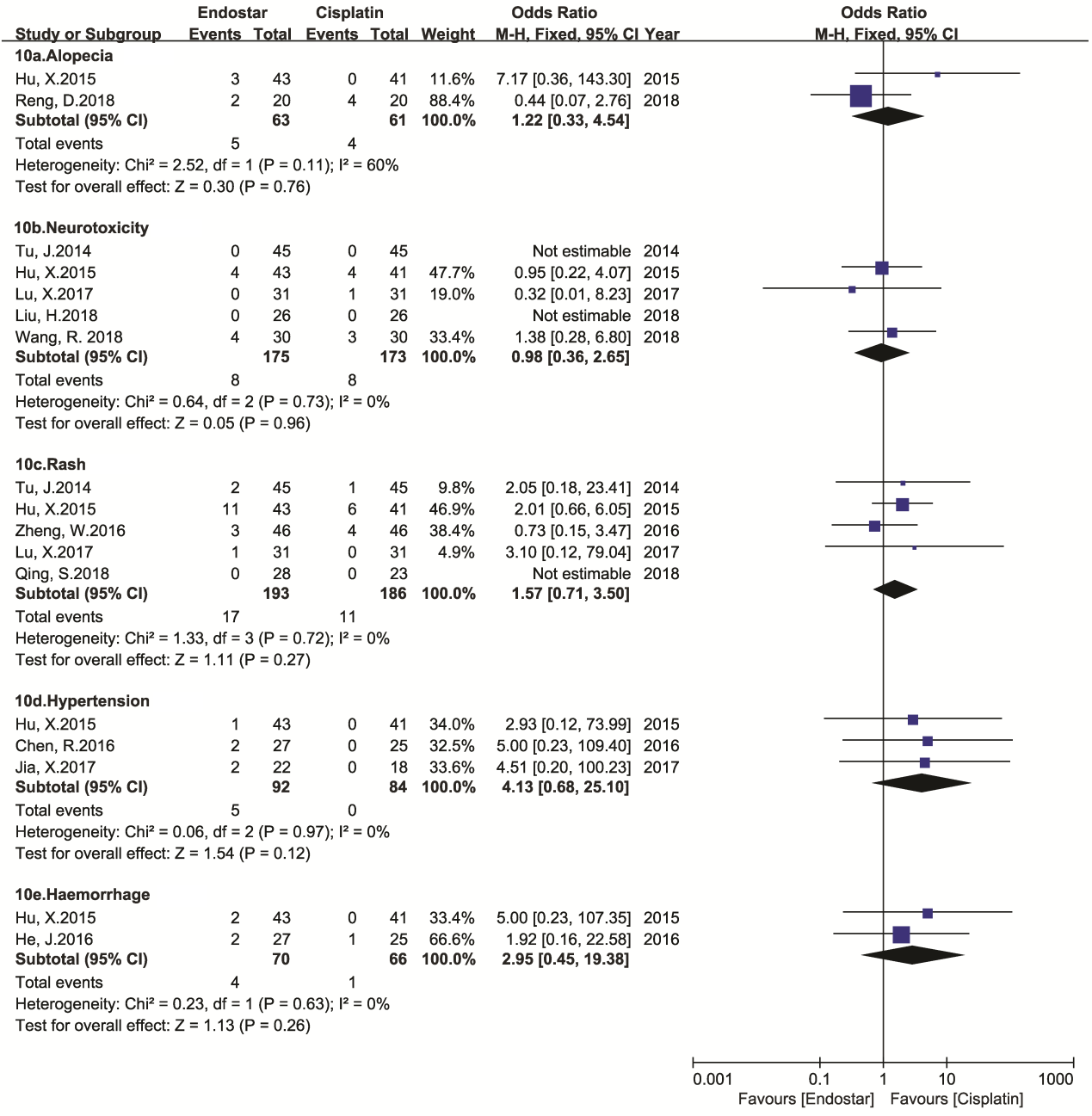
Figure.S10. The analysis of other toxicity between two groups**

**
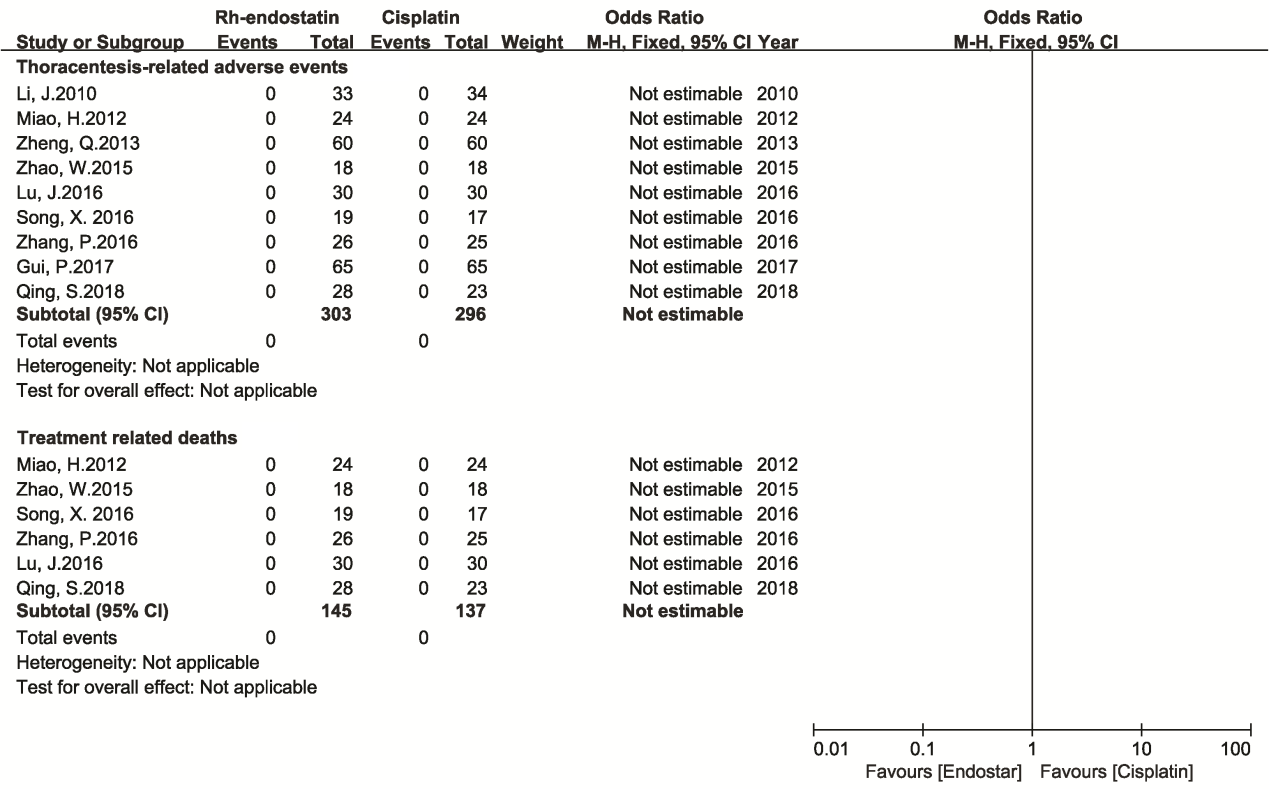
**

**Figure.S11. The analysis of TRAEs and TRDs between two groups**
