# Supplementary material for: Intrapleural Administration With Rh-Endostatin and Chemical Irritants in the Control of Malignant Pleural Effusion: A Systematic Review and Meta-Analysis
Source: Front Oncol. 2021 Aug 3;11:649999. doi: 10.3389/fonc.2021.649999 (PMC8369576; doi:10.3389/fonc.2021.649999)
Supplement: Supplementary file 1 [file DataSheet_1.zip › Supplementary Material 6A.docx]

**Supplementary Material.6A Subgroups analysis and meta-regression (Figures.S12-41)**

**
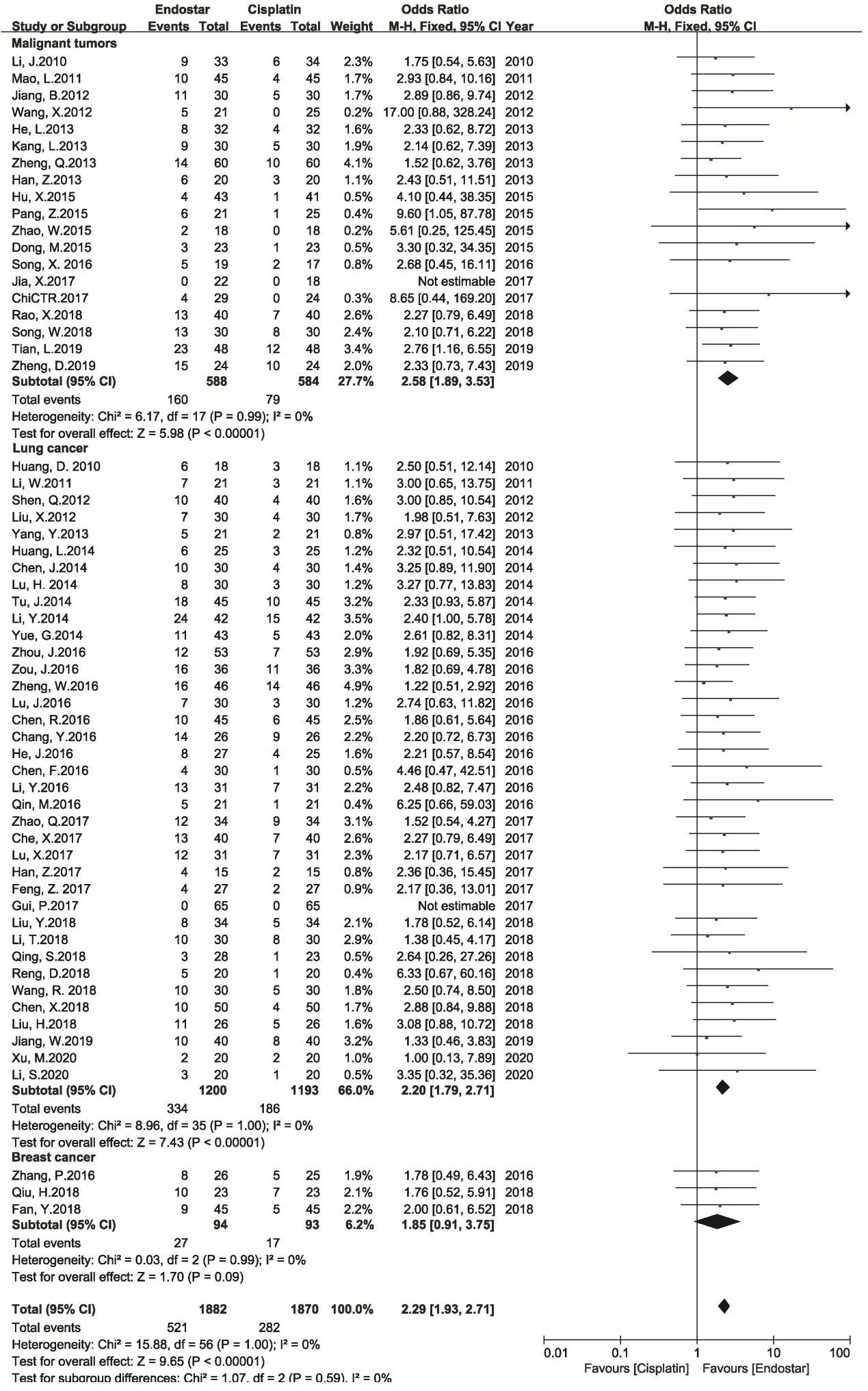
**

Figure S12. Subgroups analysis of complete response via primary tumors


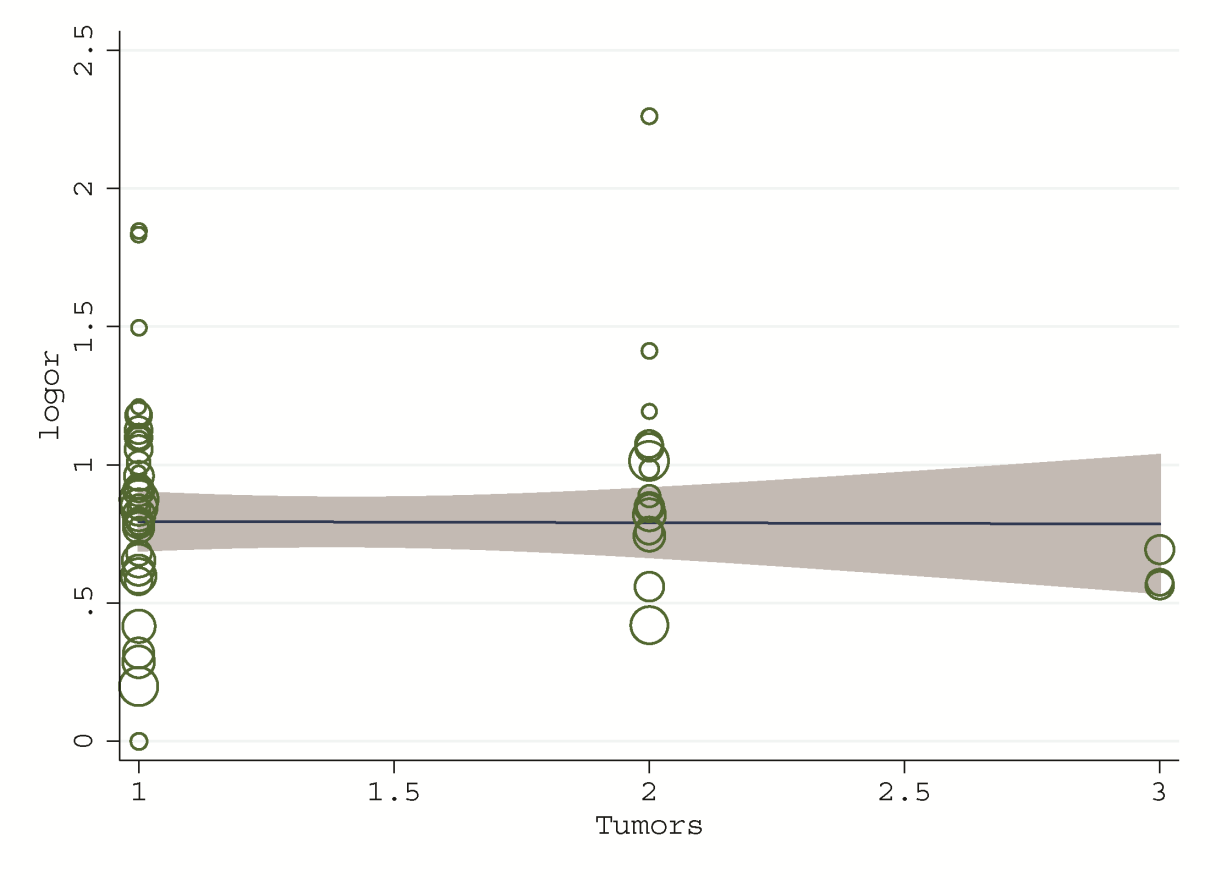
Figure S13. Meta regression of complete response via primary tumors


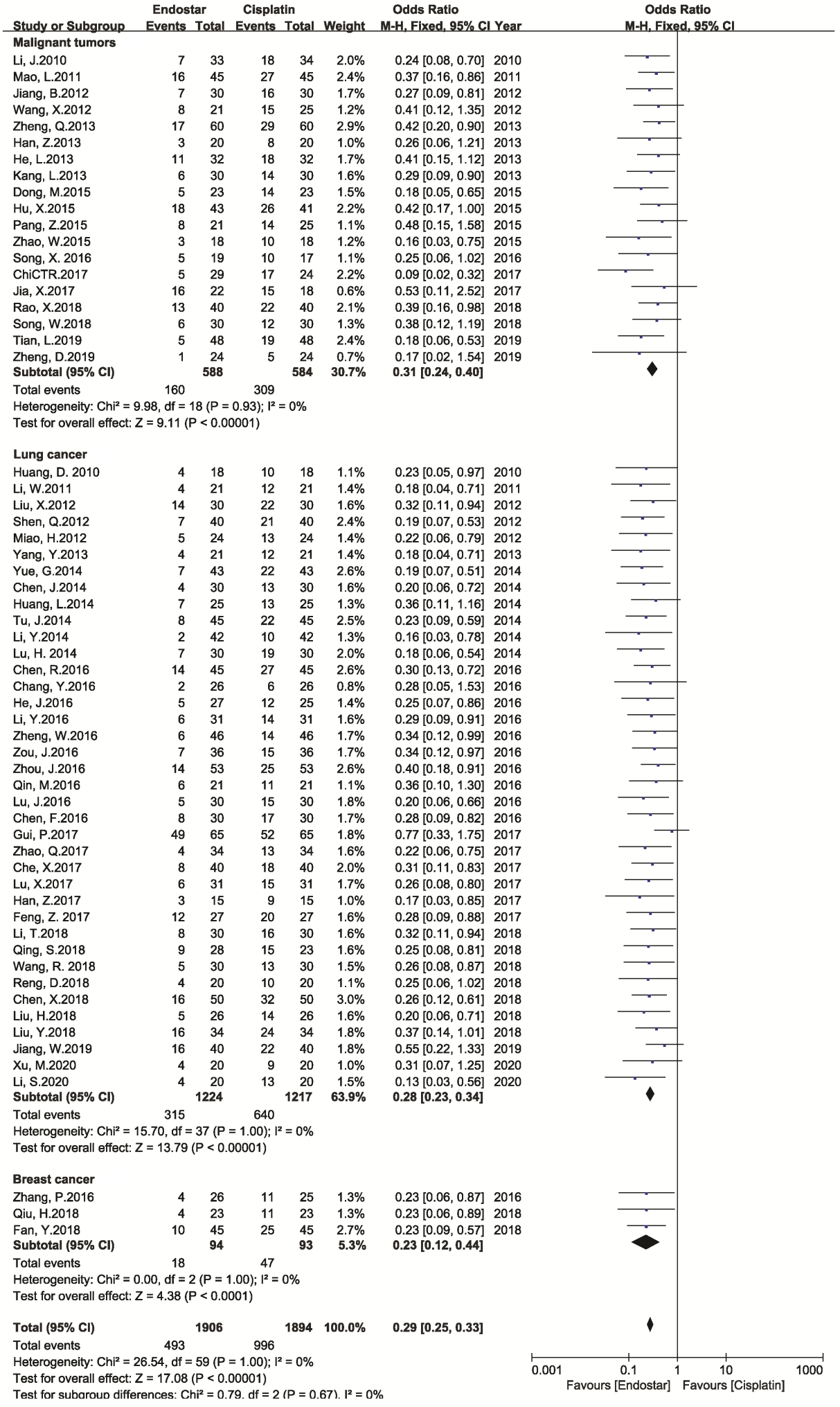
Figure S14. Subgroups analysis of treatment failure via primary tumors


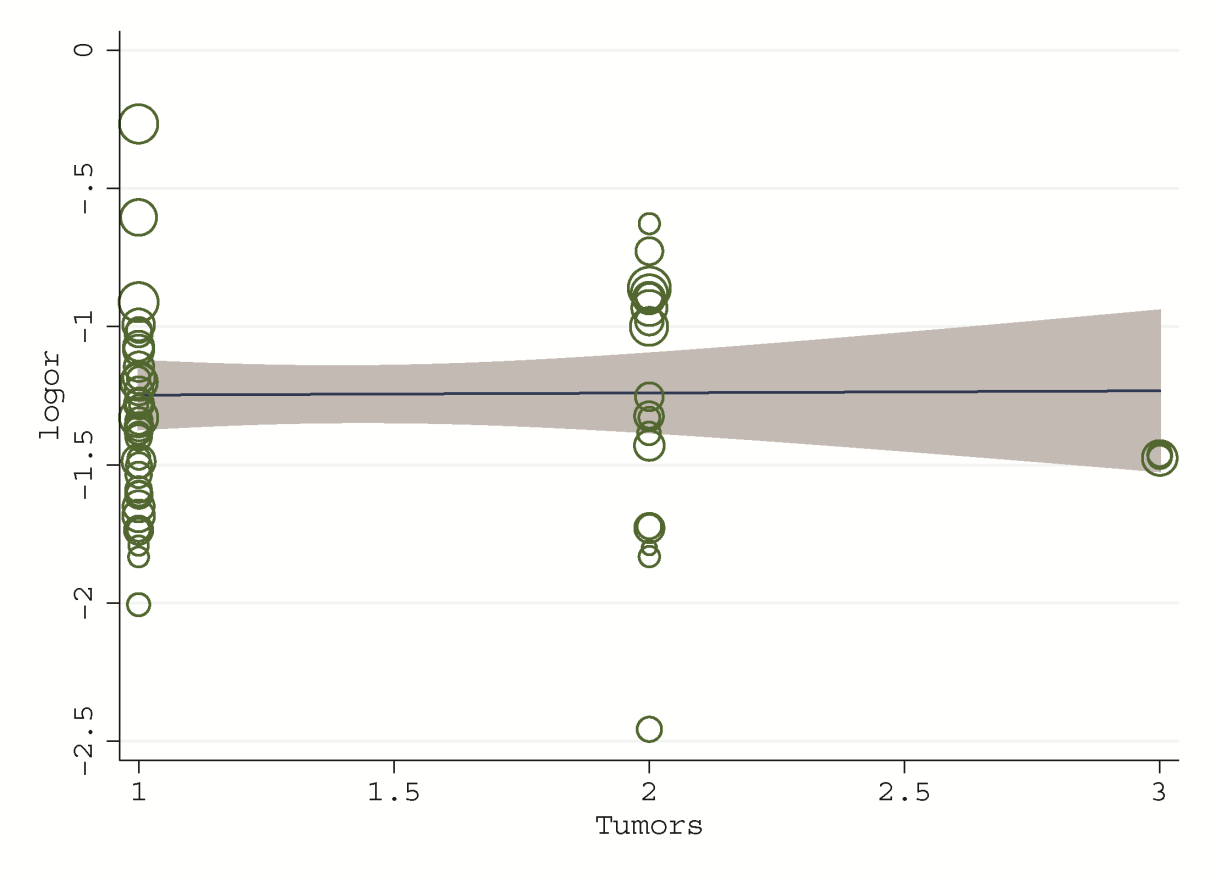
Figure S15. Meta regression of treatment failure via primary tumors


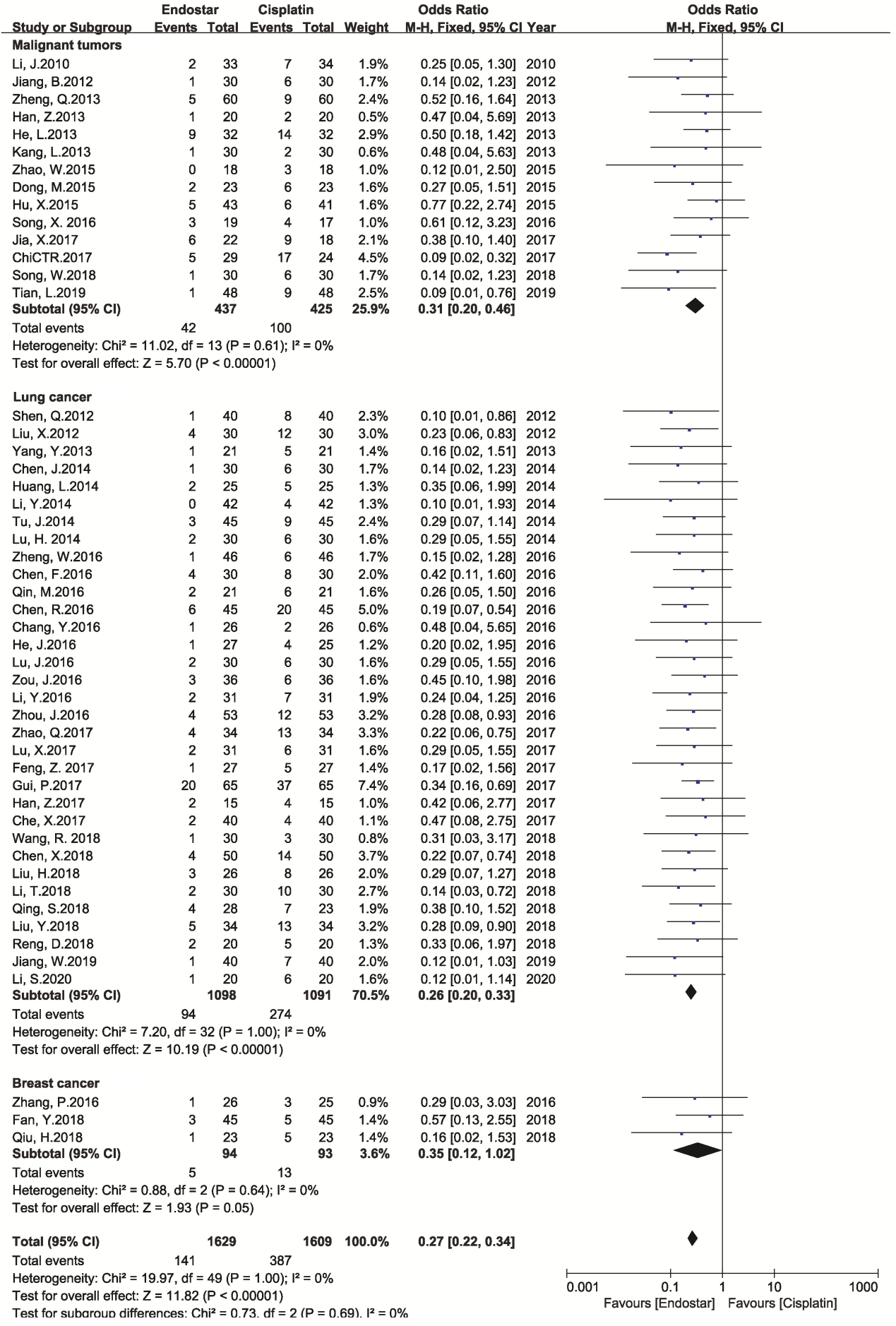
Figure S16. Subgroups analysis of treatment failure via primary tumors


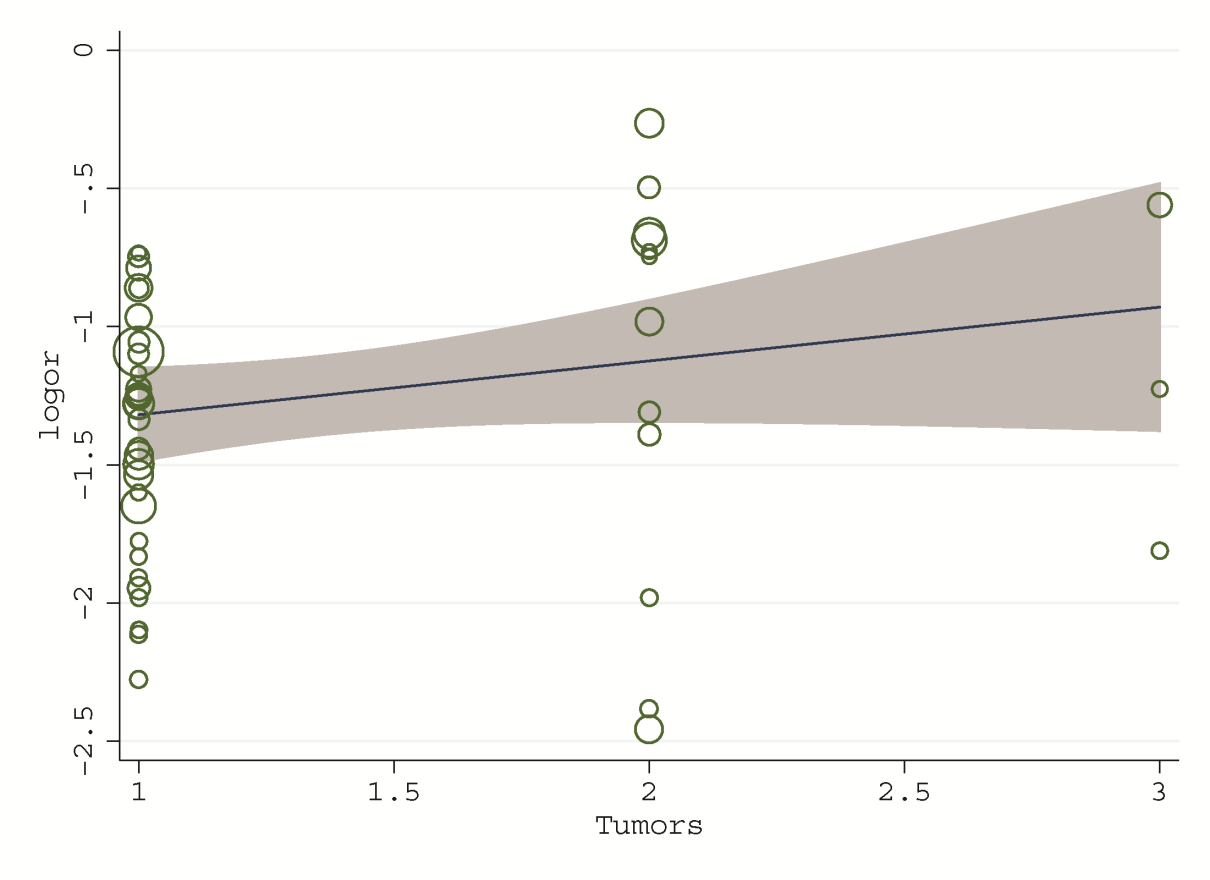
Figure S17. Meta regression of treatment failure via primary tumors


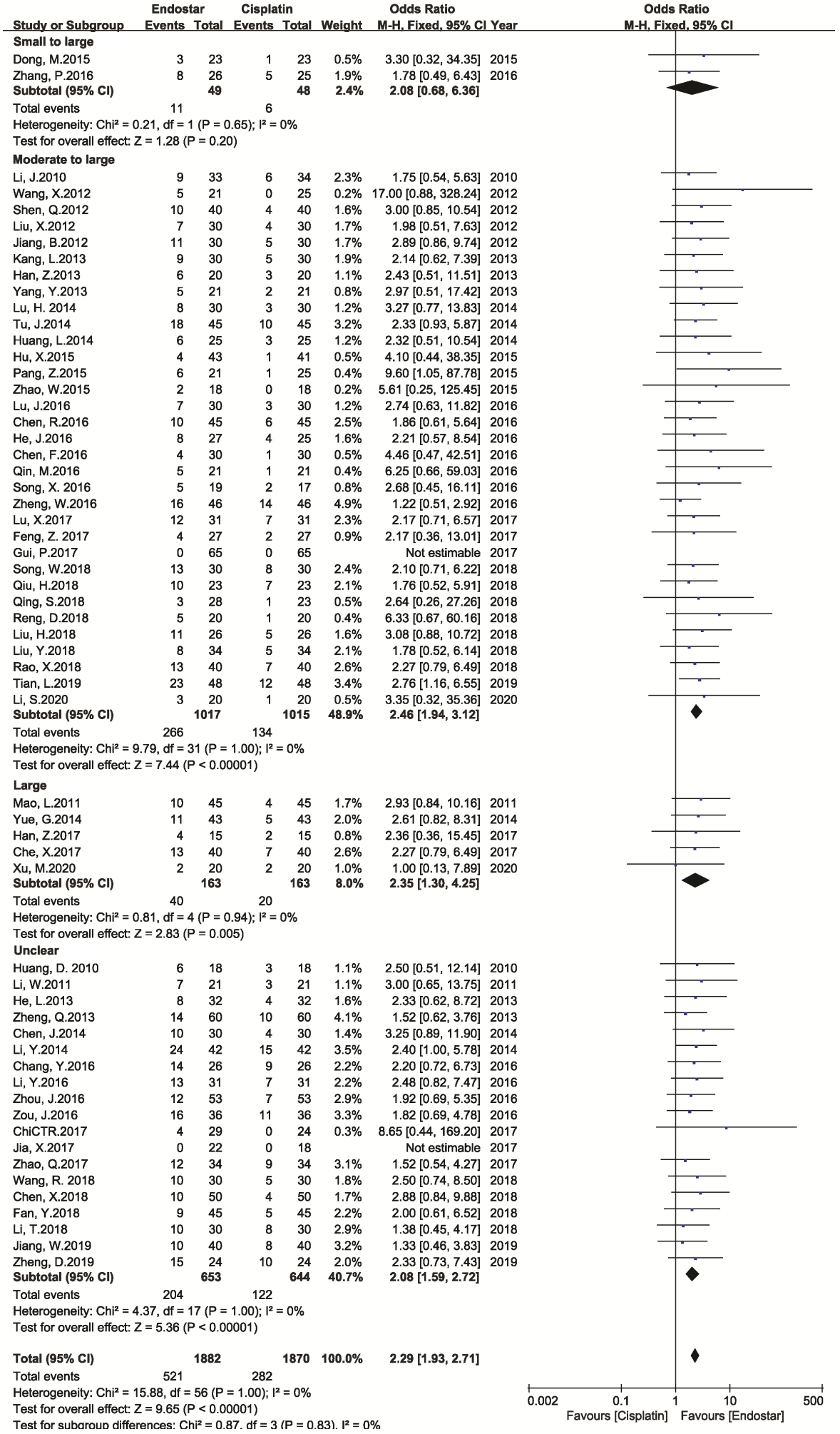


Figure S18. Subgroups analysis of complete response via pleural fluid volume


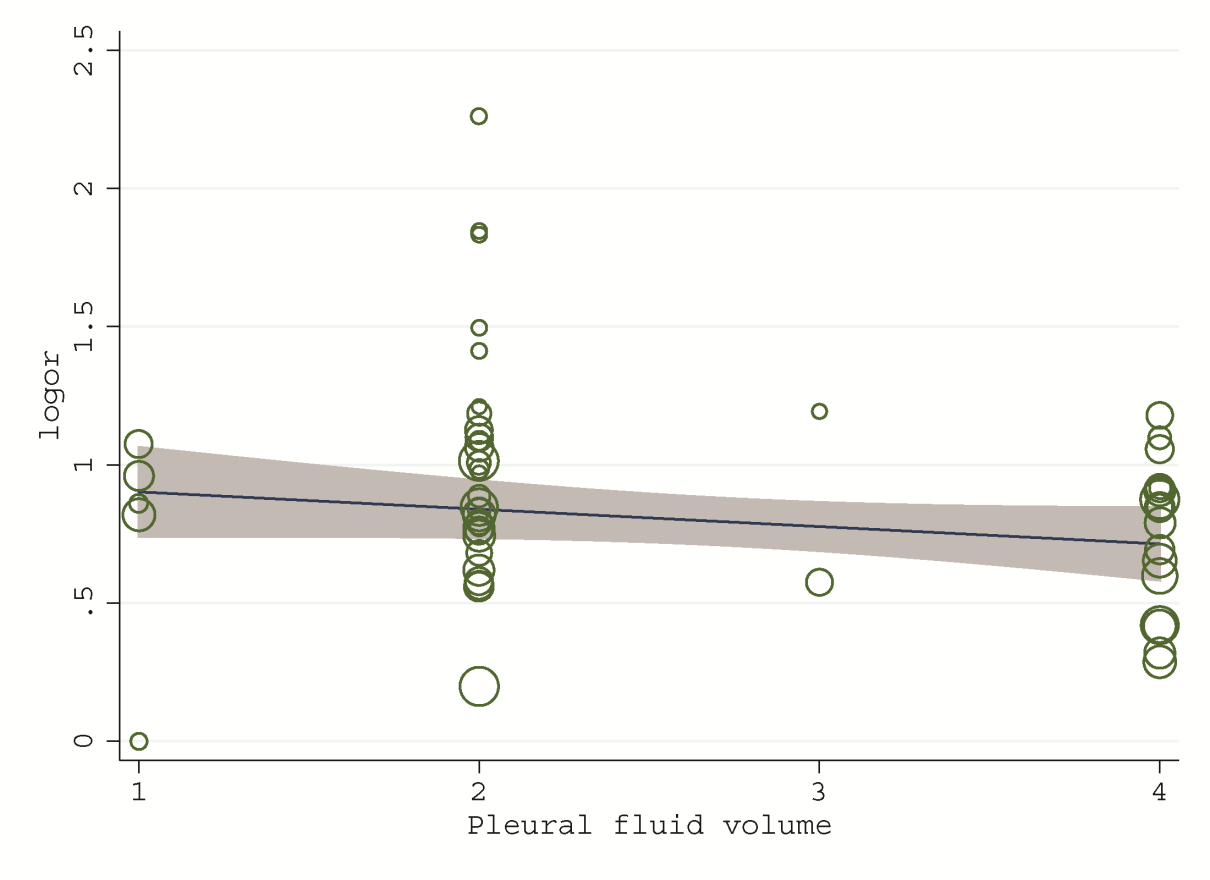
Figure S19. Meta regression of complete response via pleural fluid volume


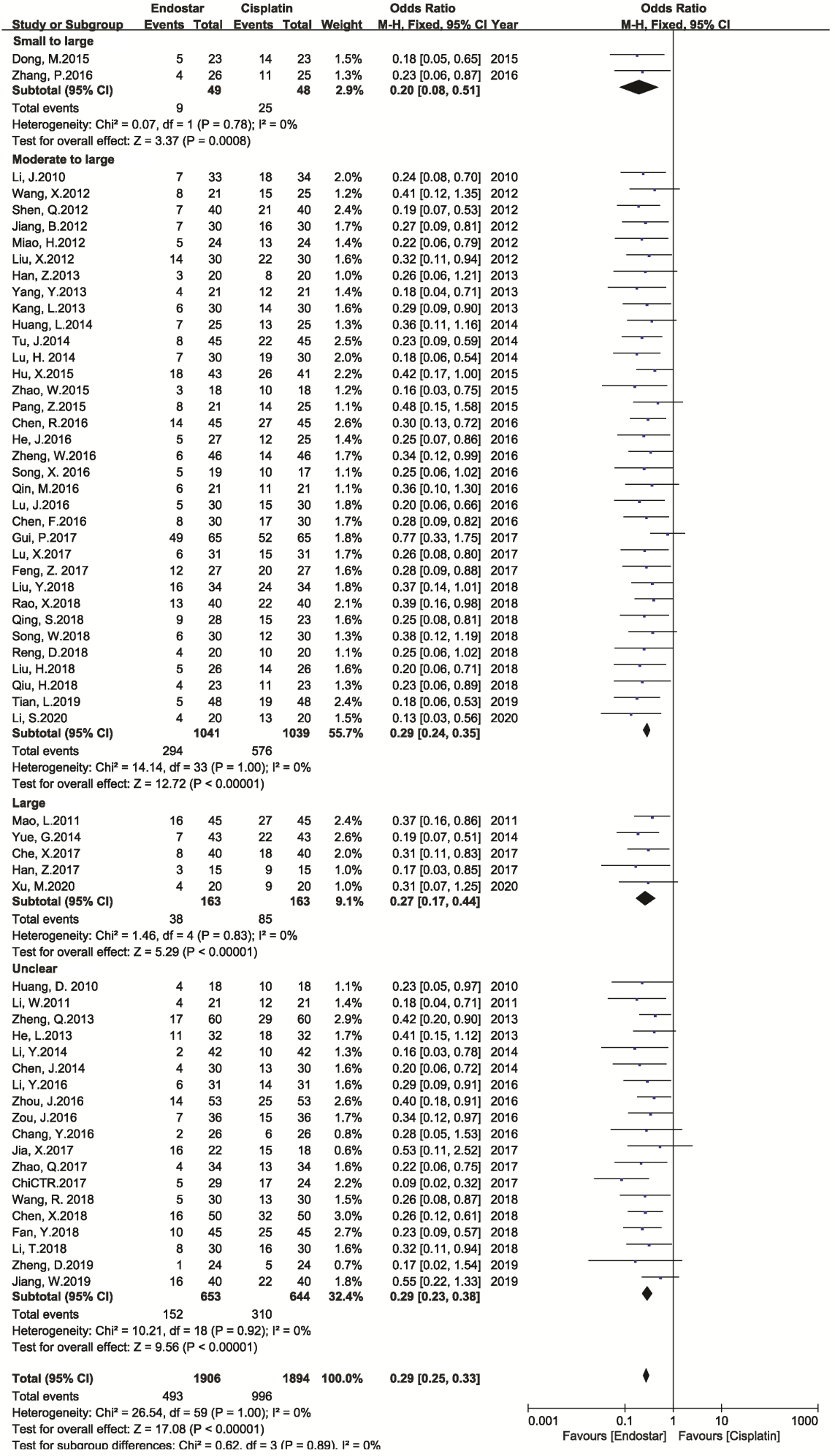


Figure S20 Subgroups analysis of treatment failure via pleural fluid volume


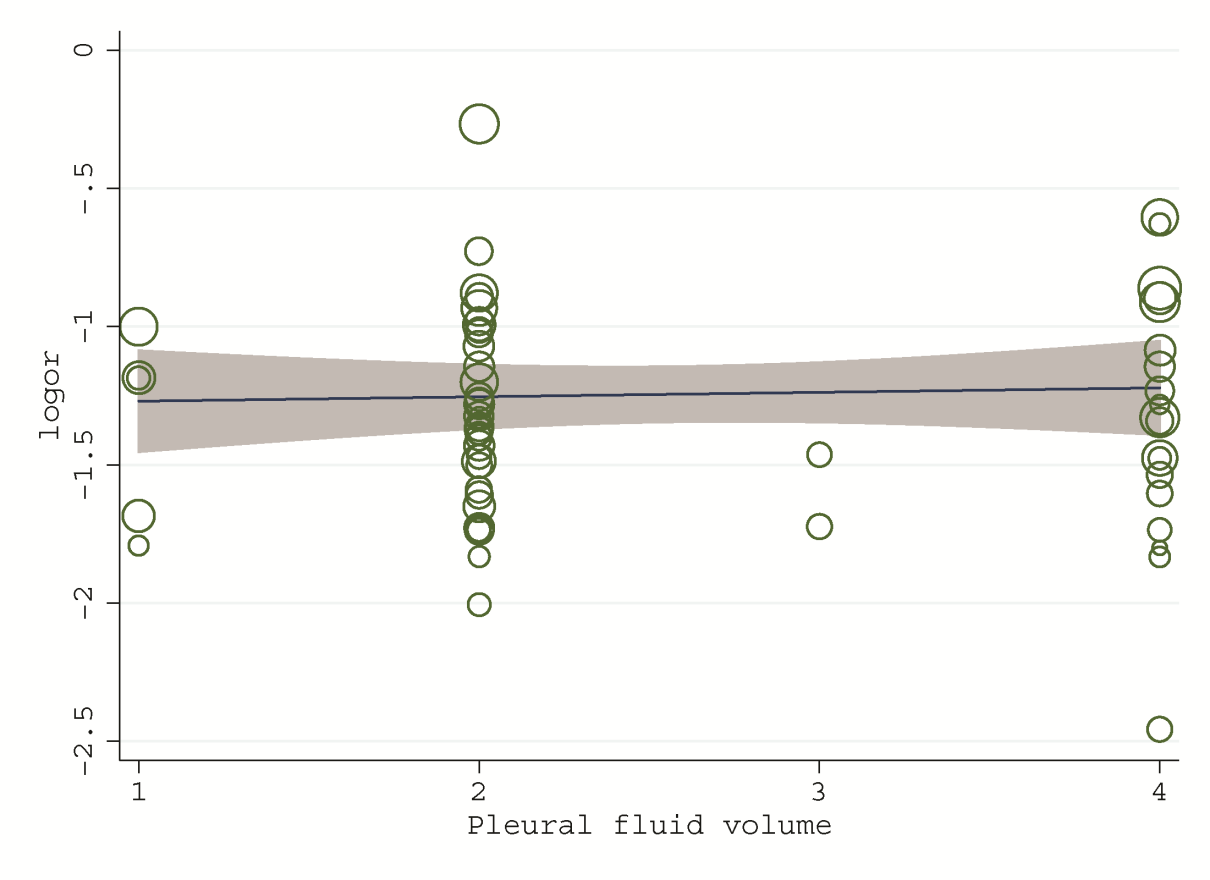
Figure S21 Meta regression of treatment failure via pleural fluid volume


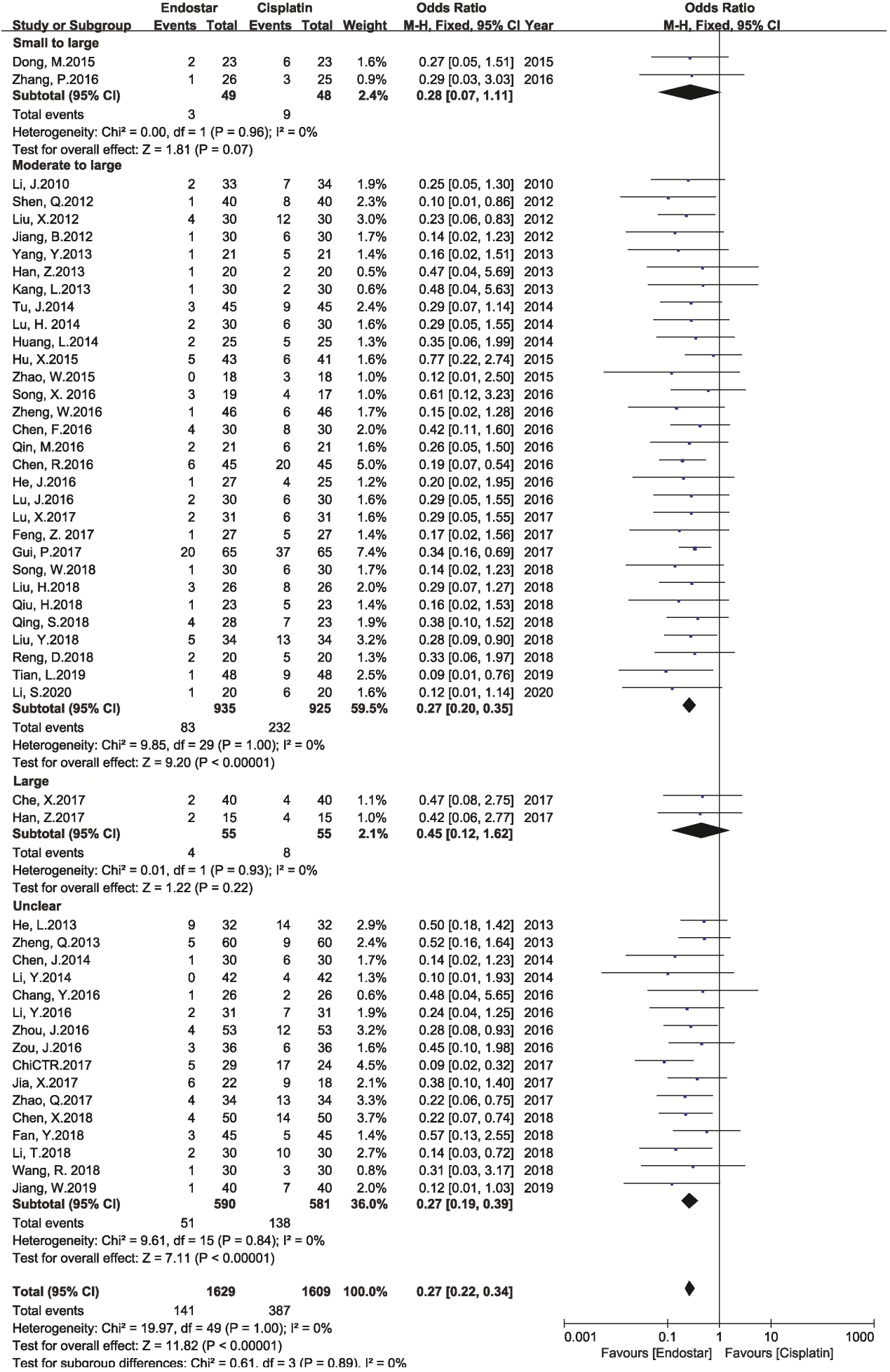
Figure S22 Subgroups analysis of treatment failure via pleural fluid volume


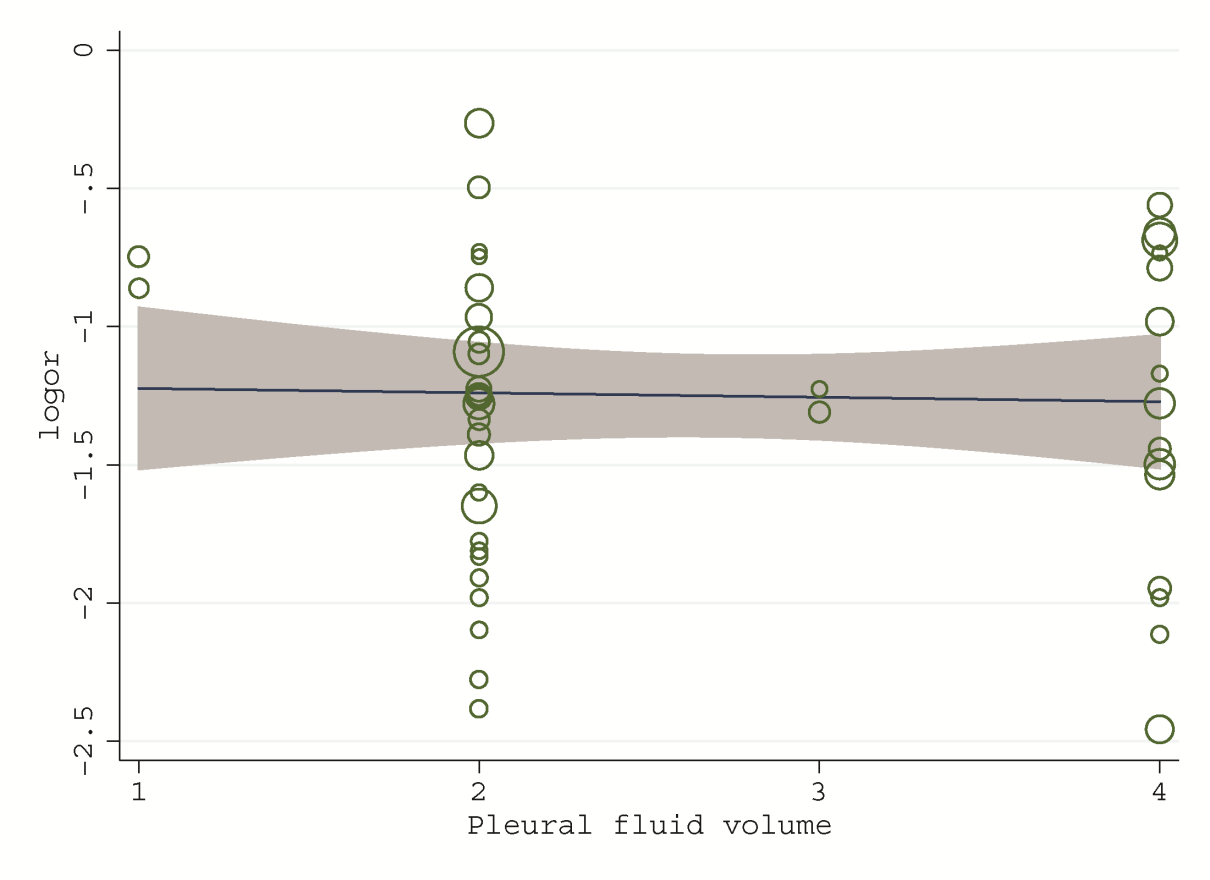
Figure S23 Meta regression of treatment failure via pleural fluid volume


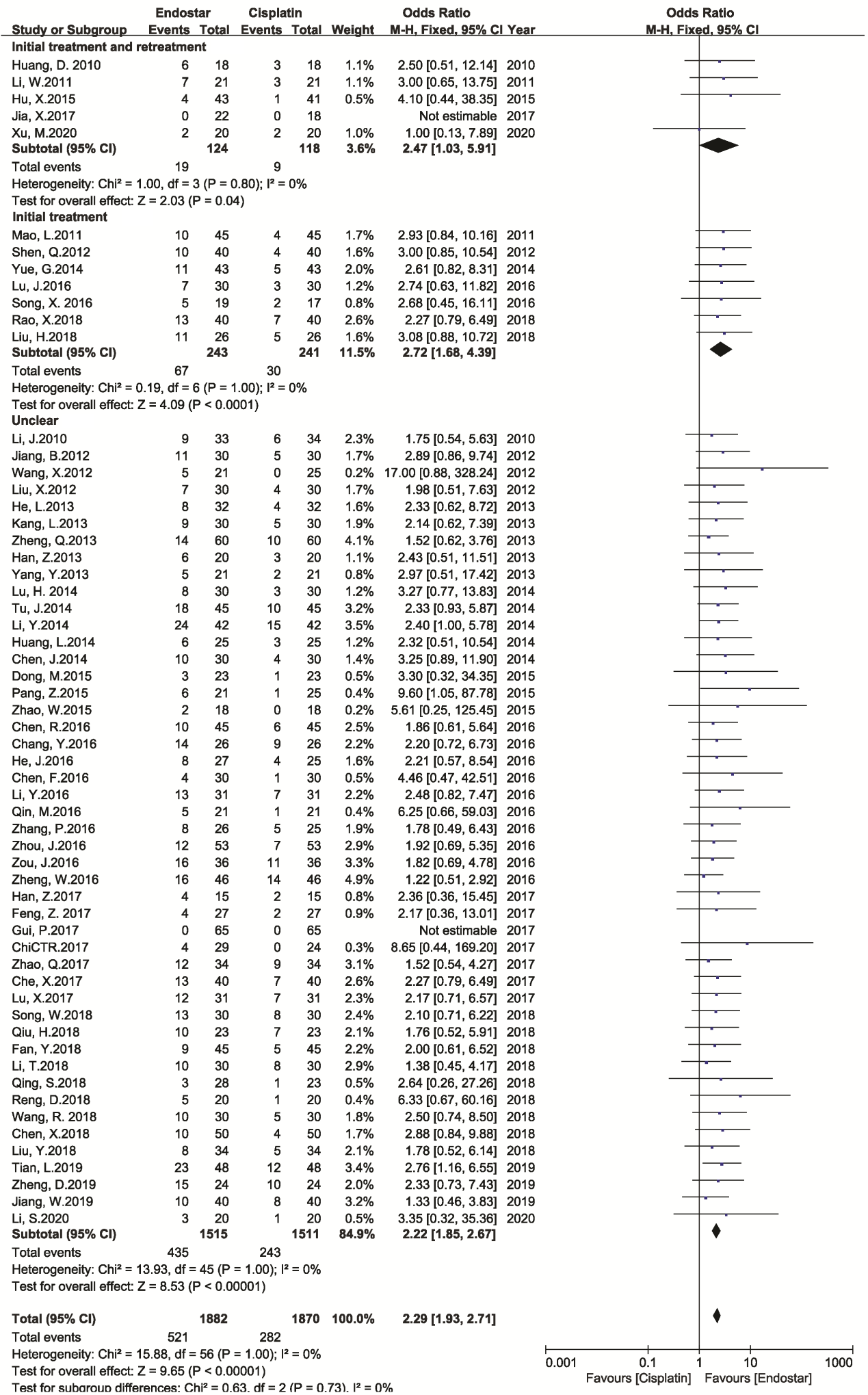


Figure S24. Subgroups analysis of complete response via treatment history


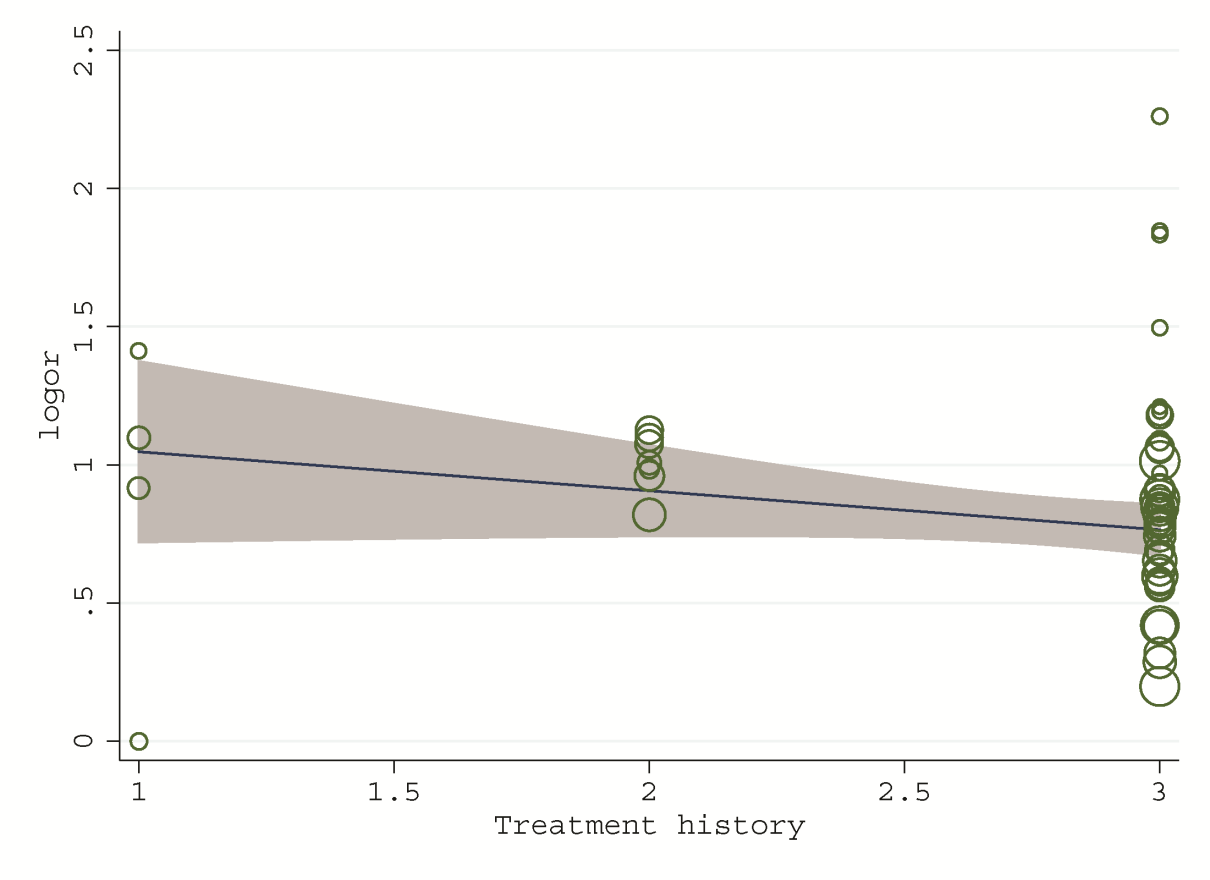


Figure S25. Meta regression of complete response via treatment history


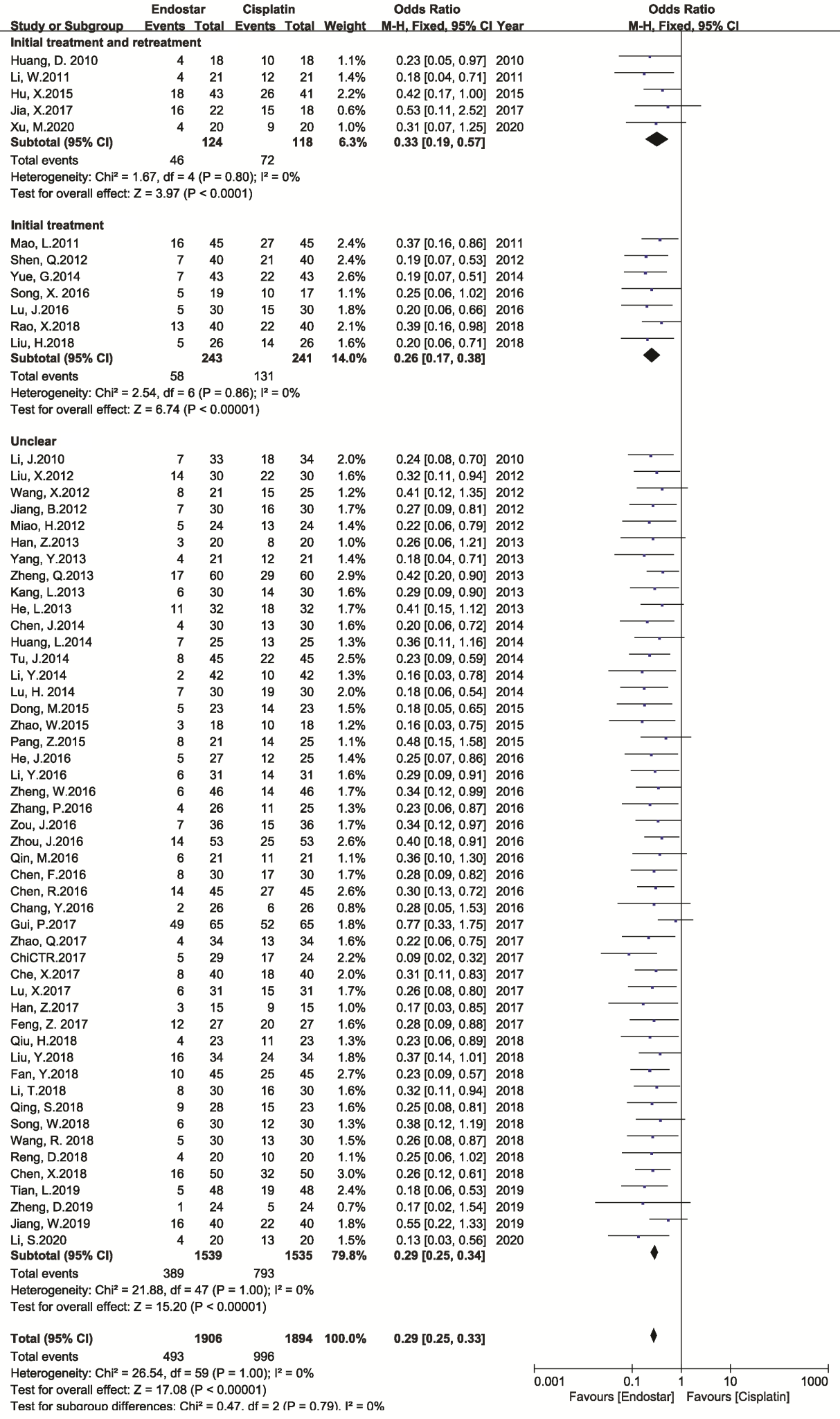


Figure S26 .Subgroups analysis of treatment failure via treatment history


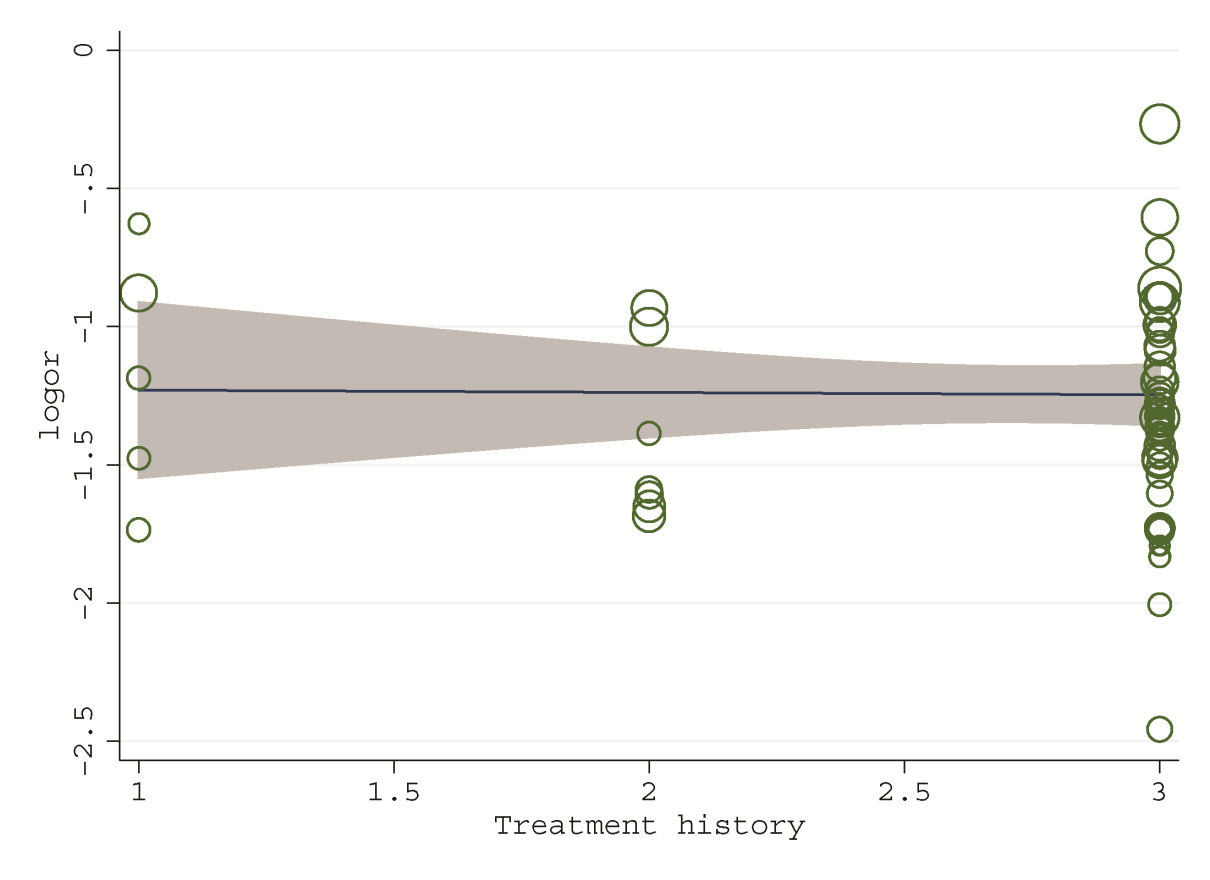


Figure S27. Meta regression of treatment failure via treatment history


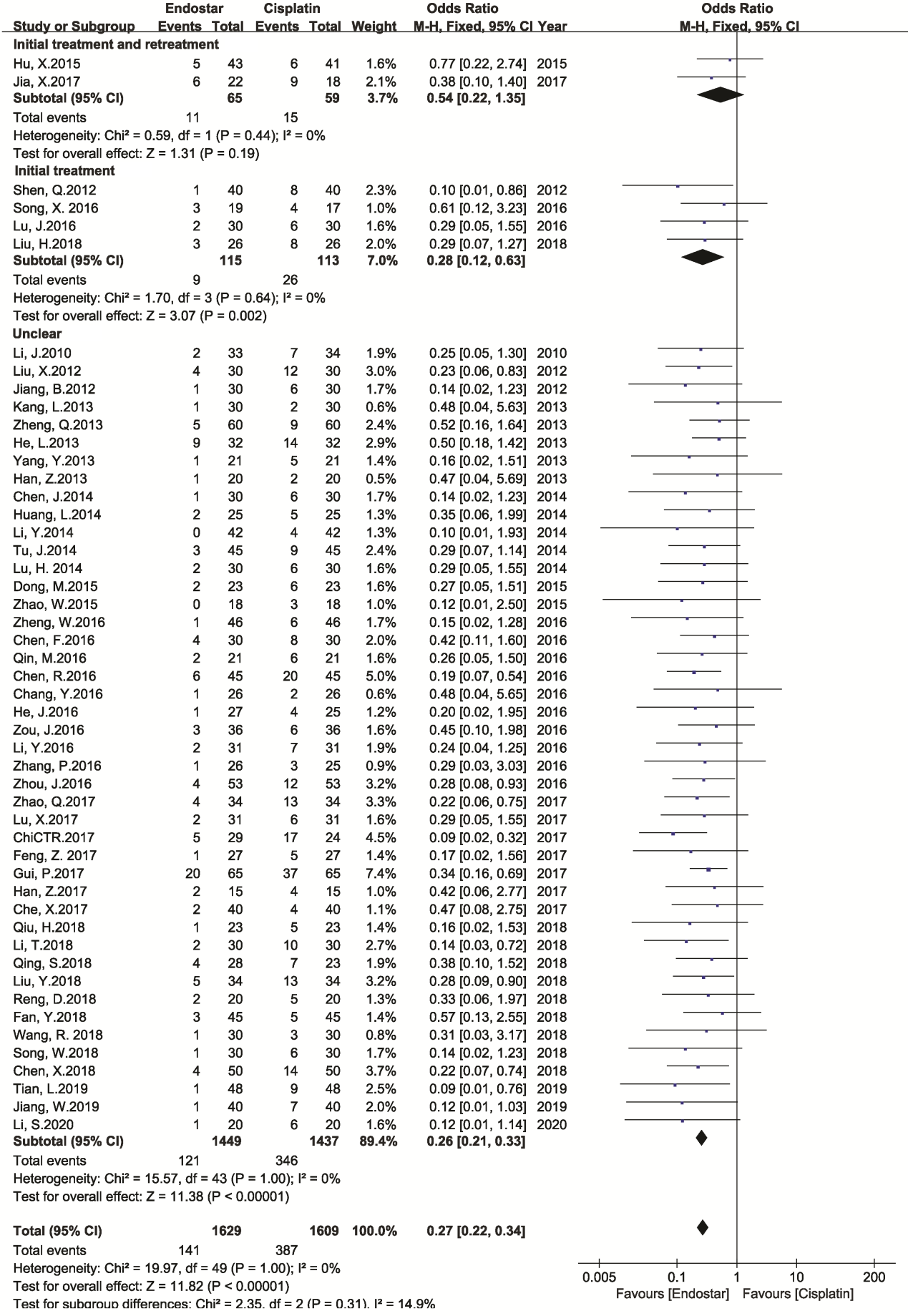


Figure S28. Subgroups analysis of treatment failure via treatment history


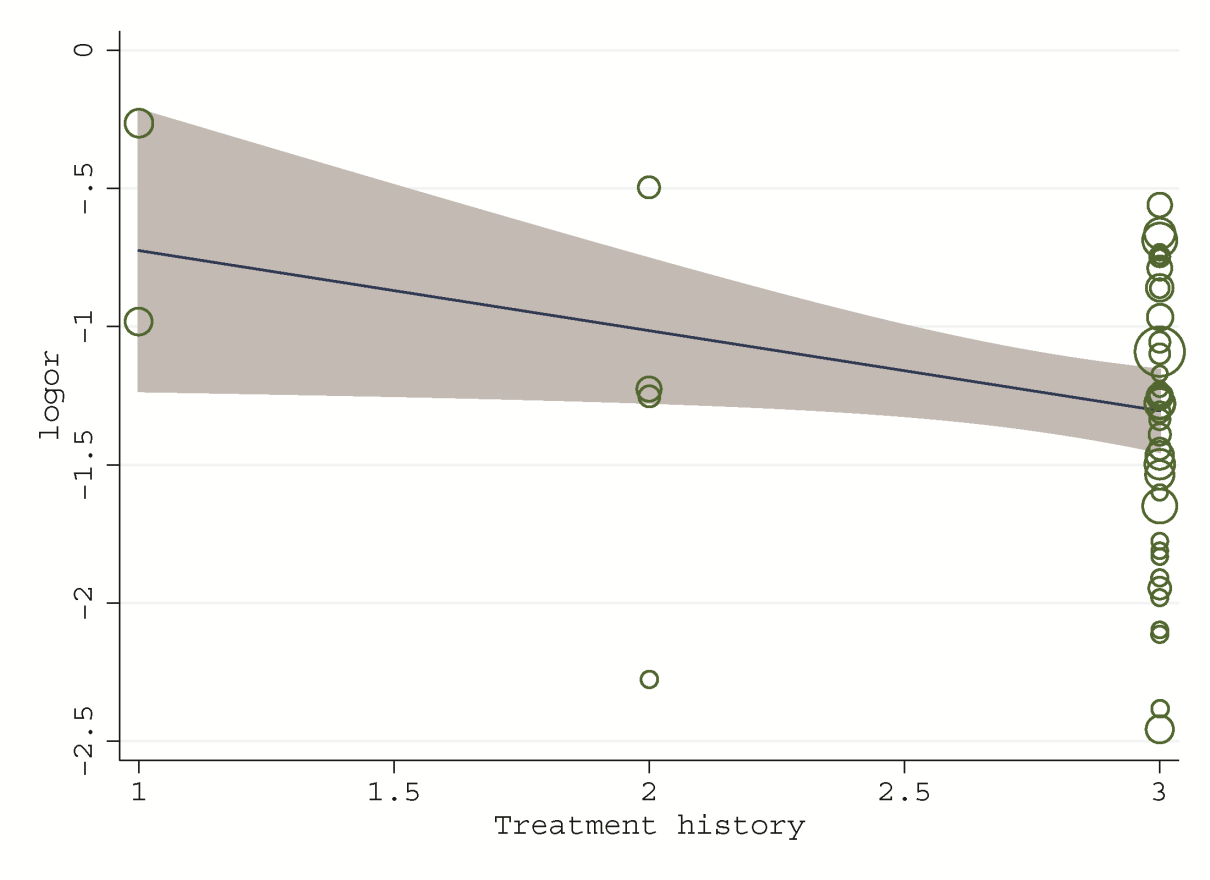


Figure S29. Meta regression of treatment failure via treatment history


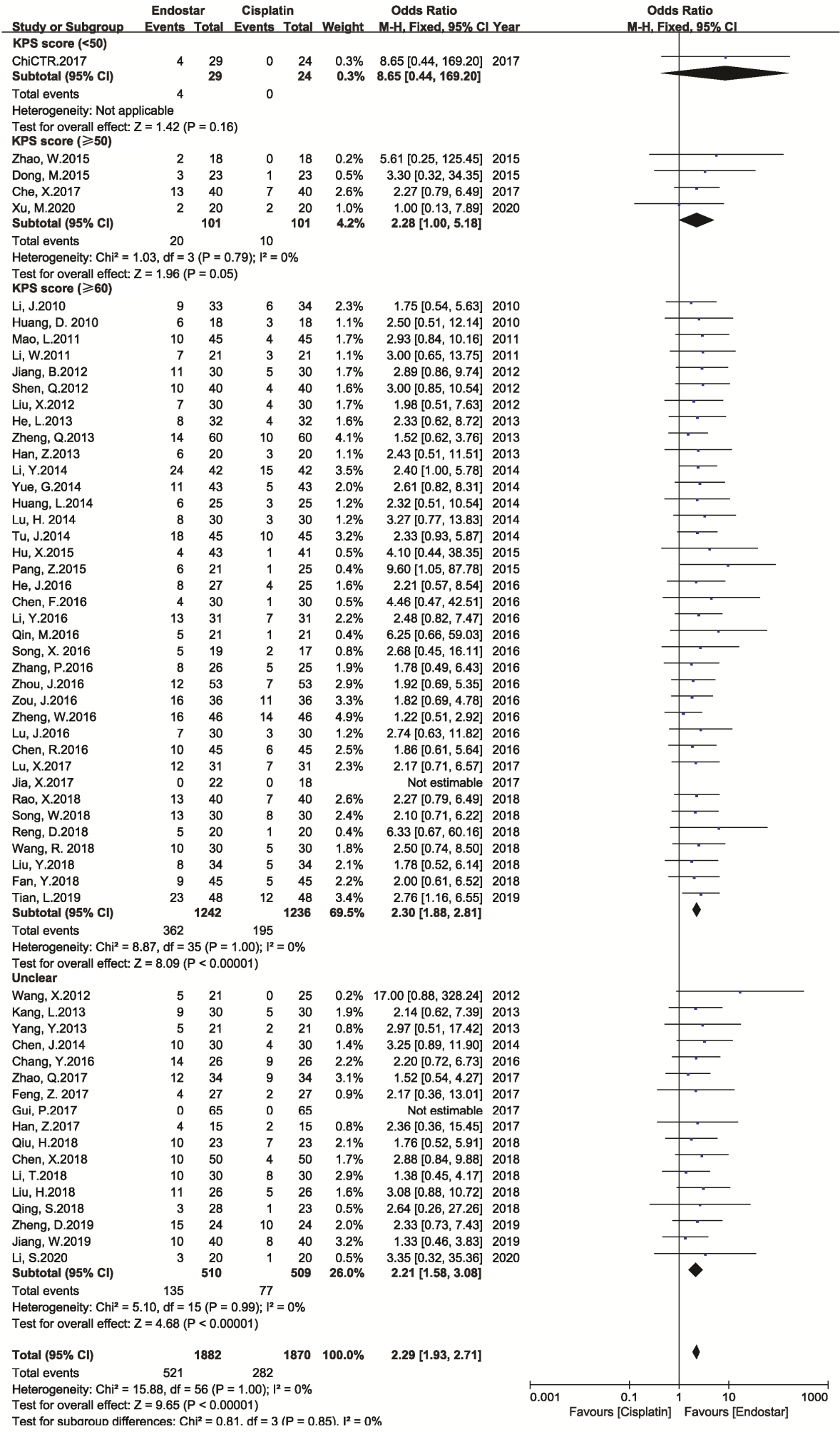


Figure S30. Subgroups analysis of complete response via KPS score


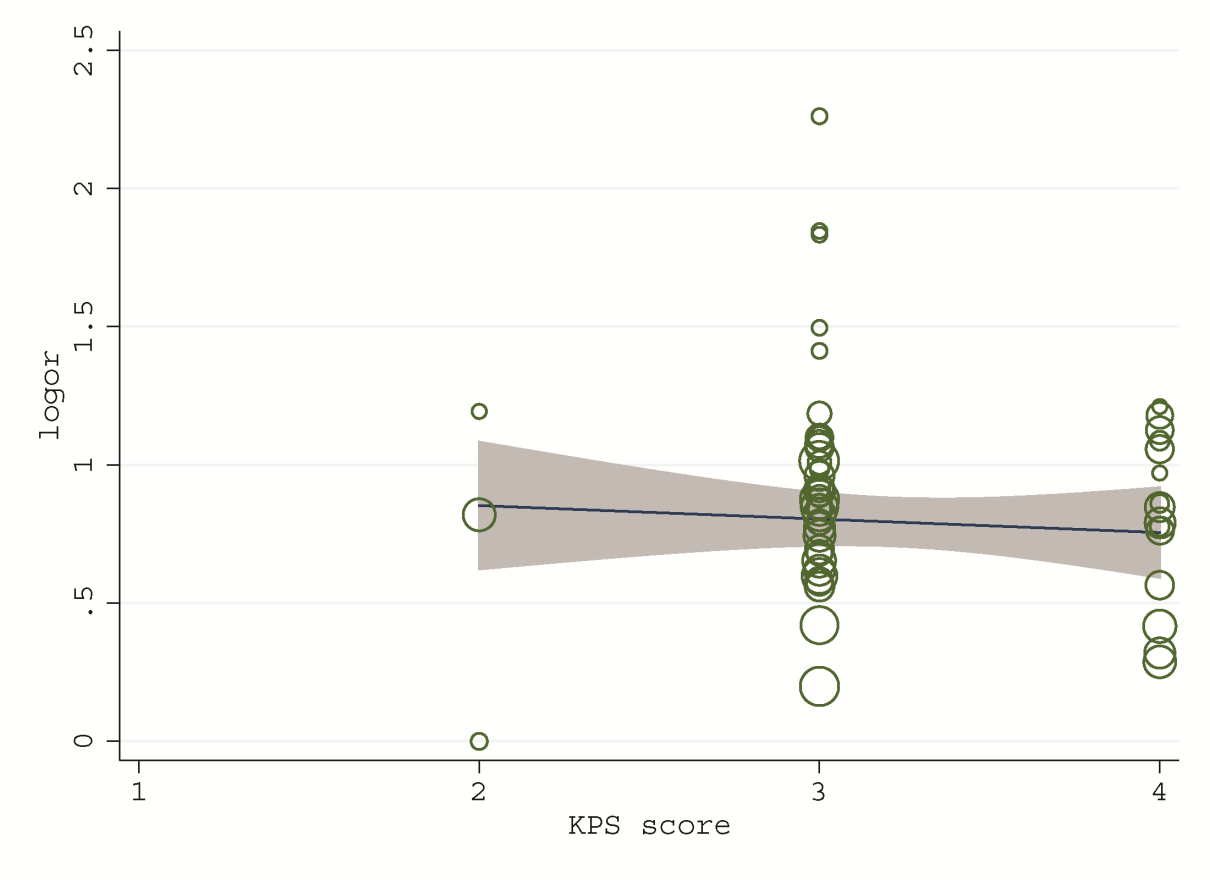


Figure S31. Meta regression of complete response via KPS score


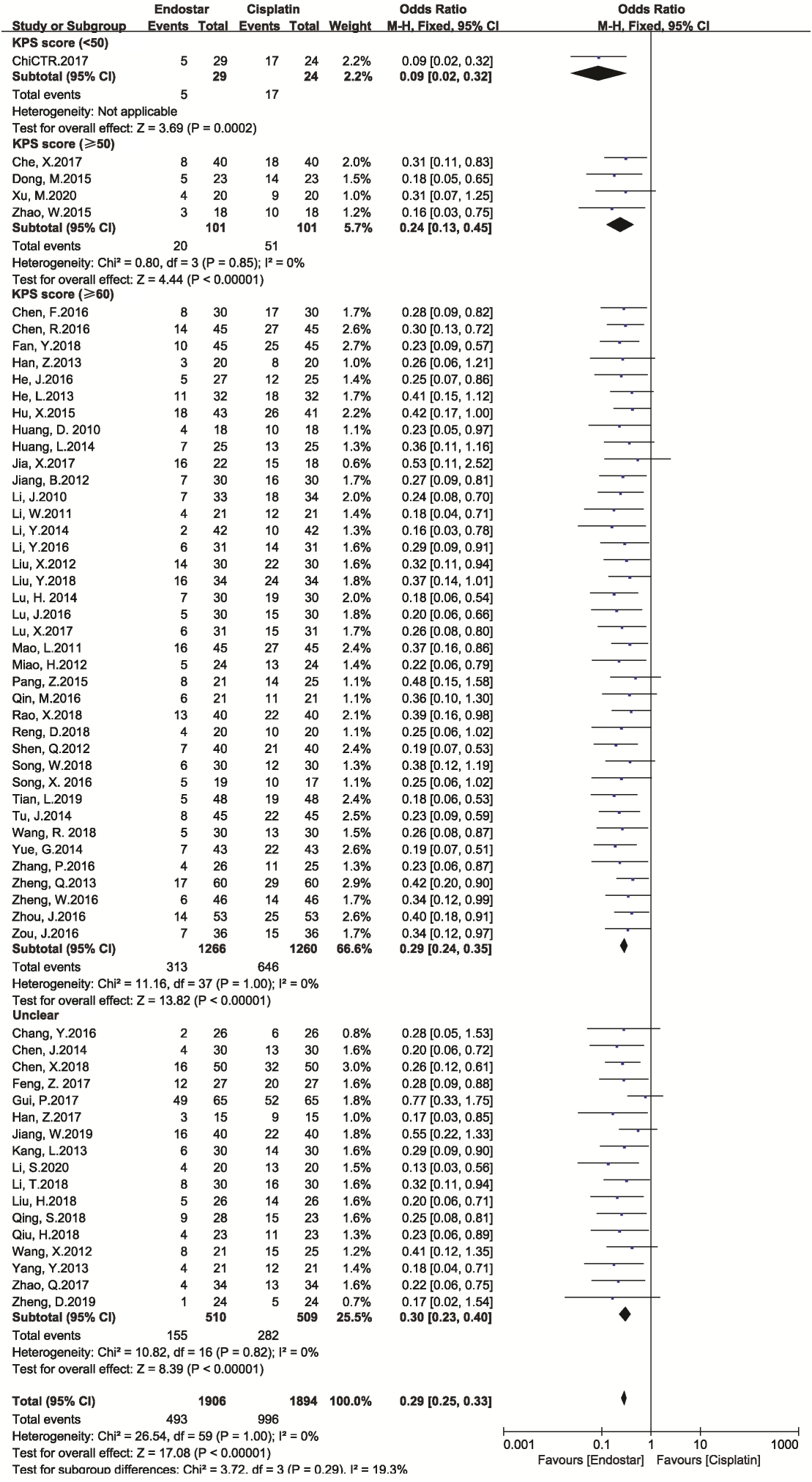


Figure S32. Subgroups analysis of treatment failure via KPS score


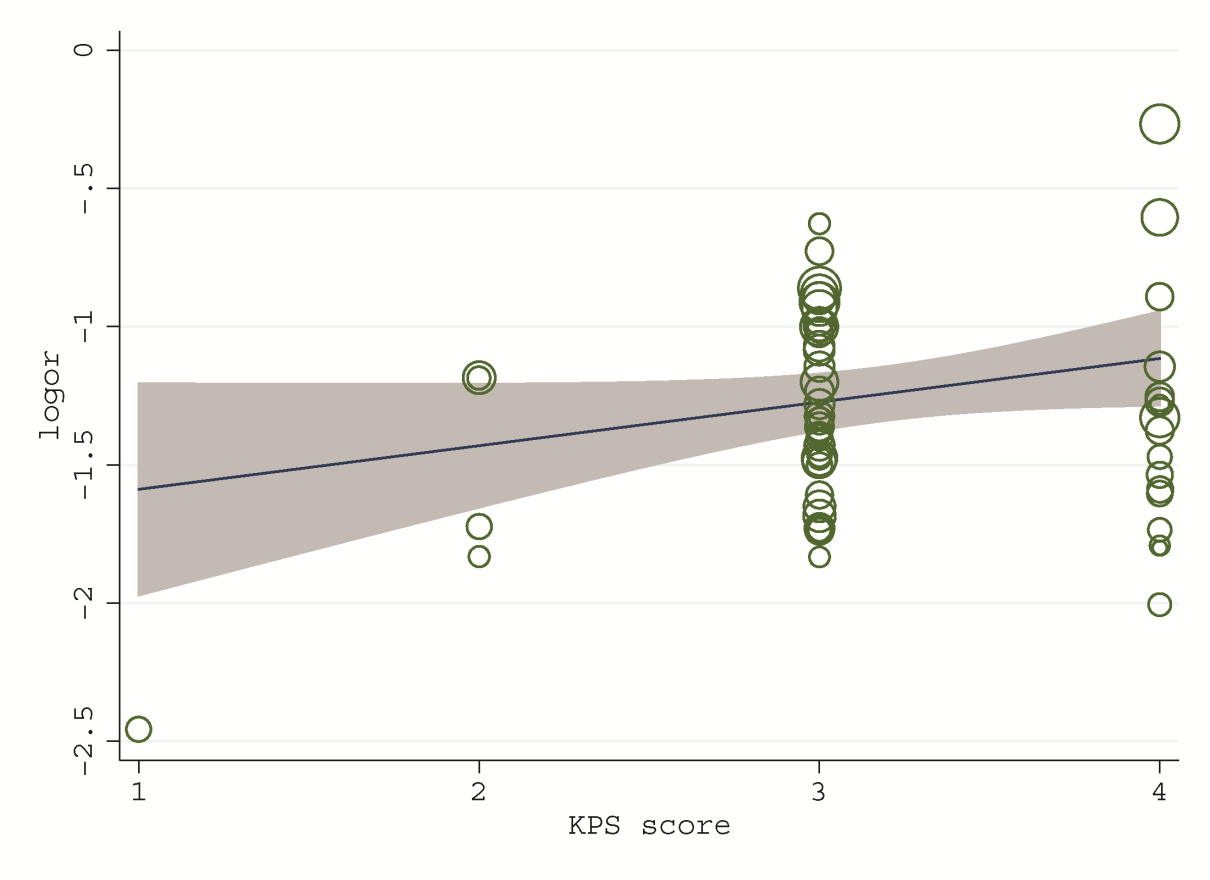
Figure S33. Meta regression of treatment failure via KPS score


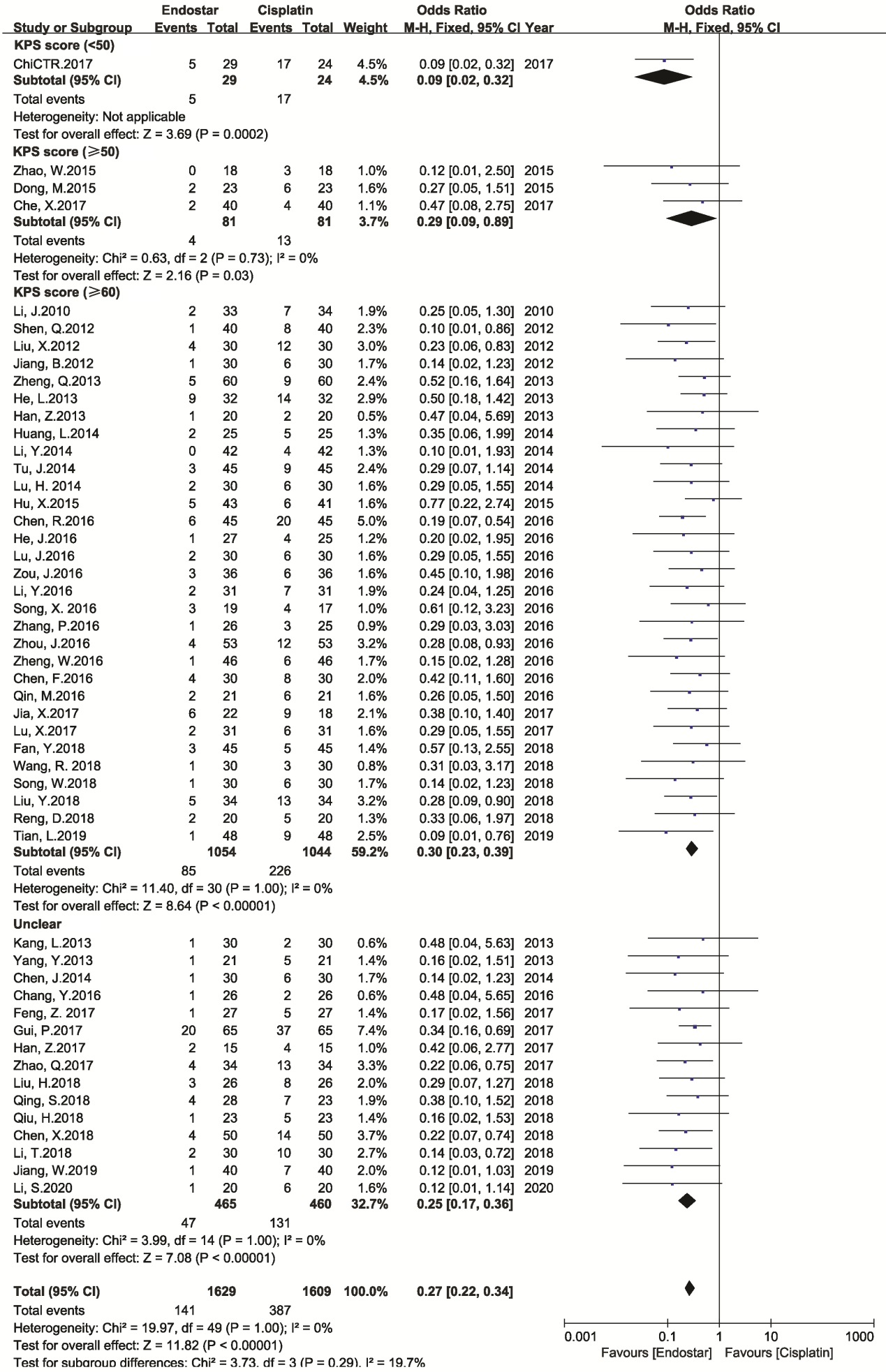


Figure S34. Subgroups analysis of treatment failure via KPS score


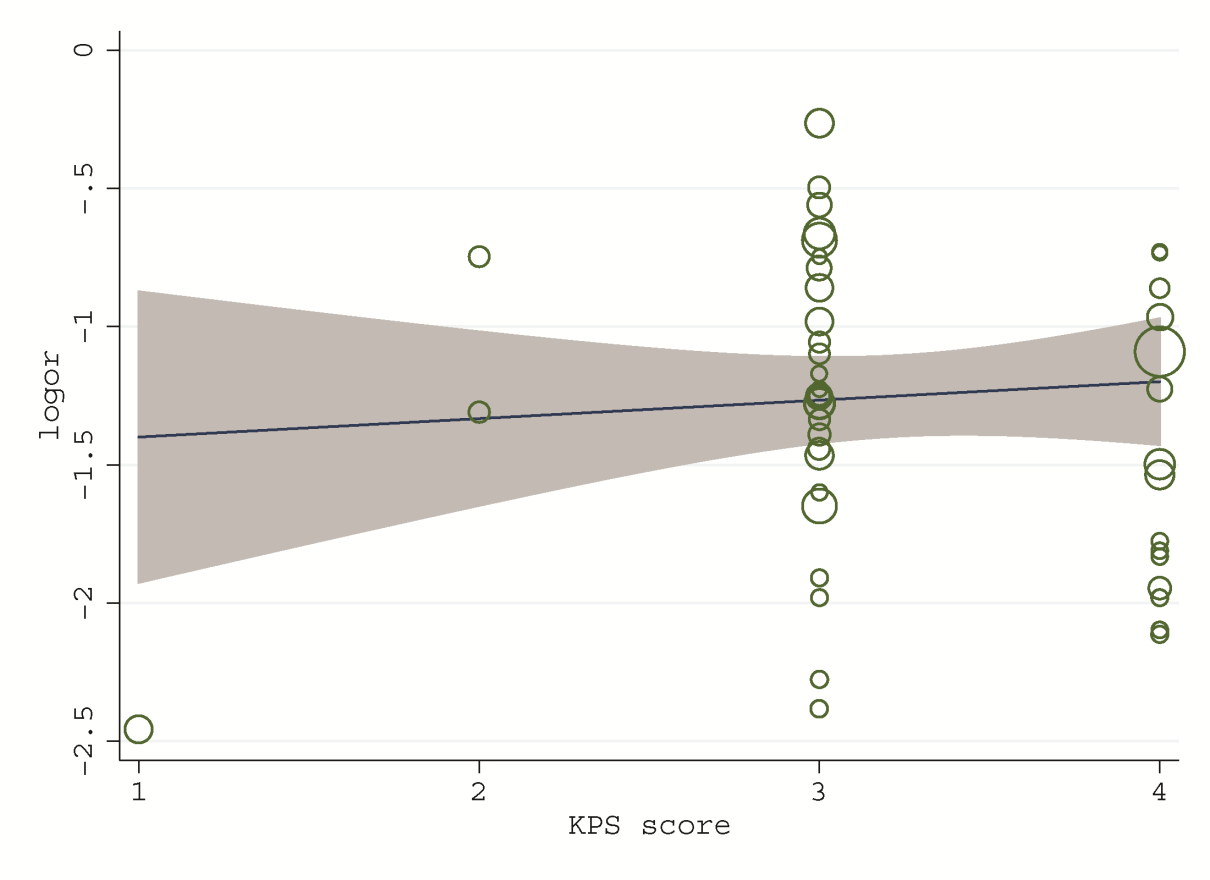
Figure S35.Meta regression of treatment failure via KPS score


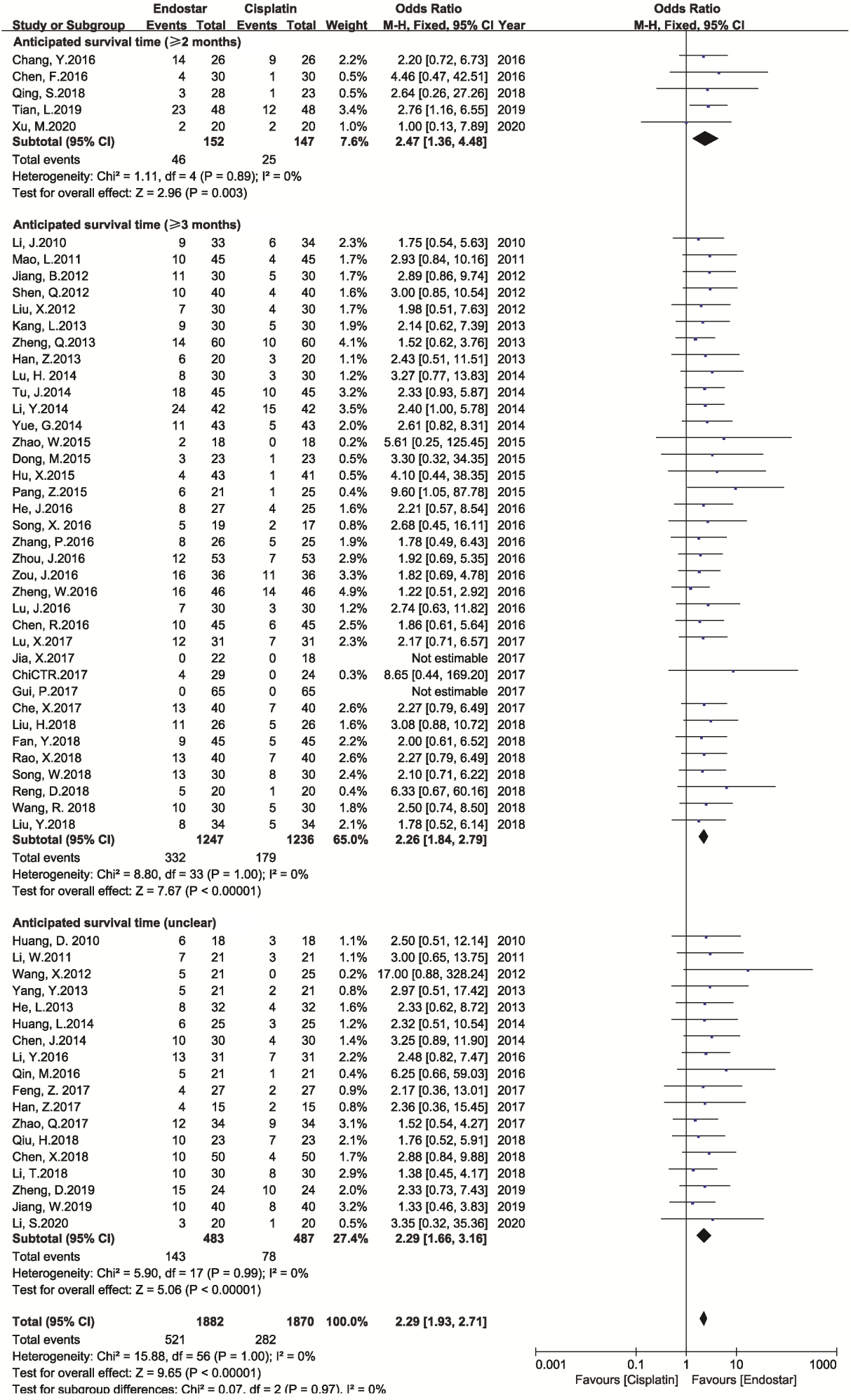
Figure S36 .Subgroups analysis of complete response via anticipated survival time


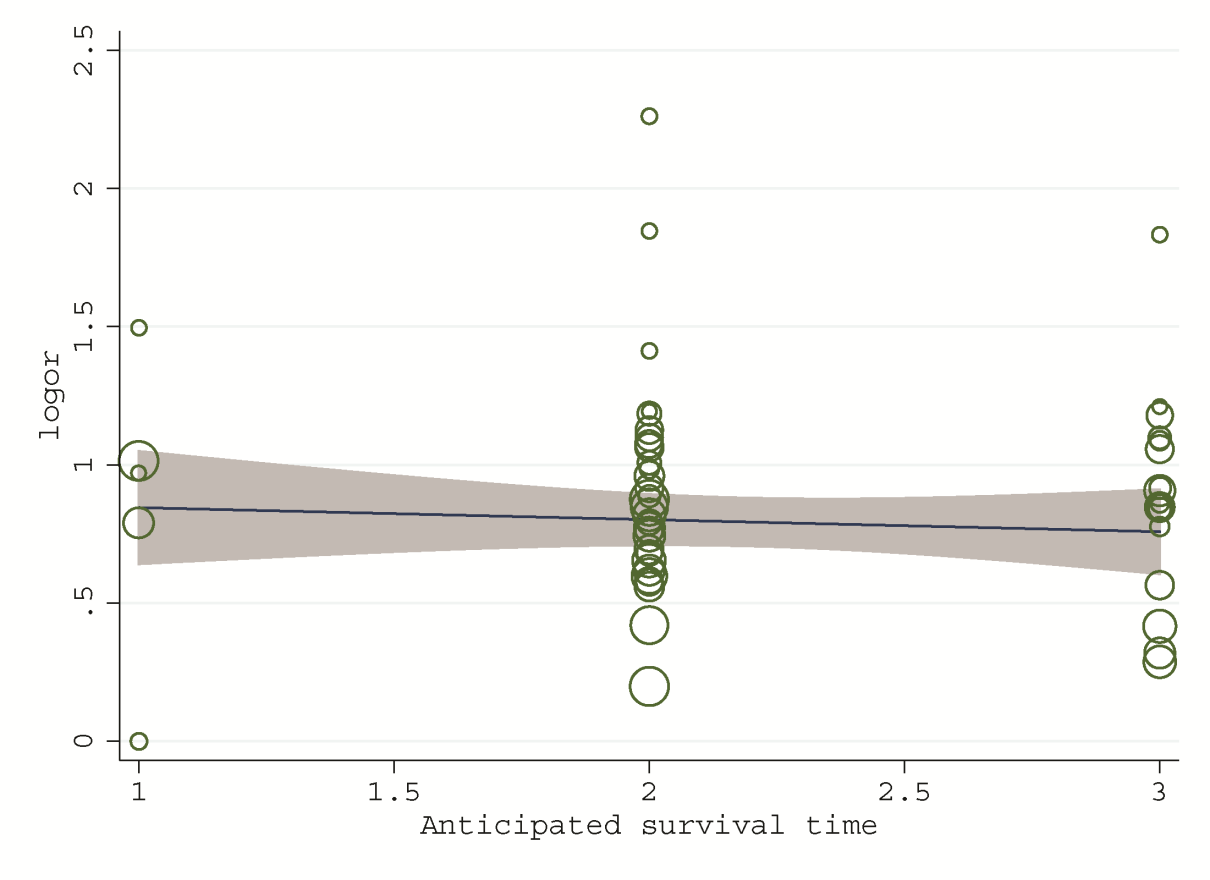


Figure S37.Meta regression of complete response via anticipated survival time


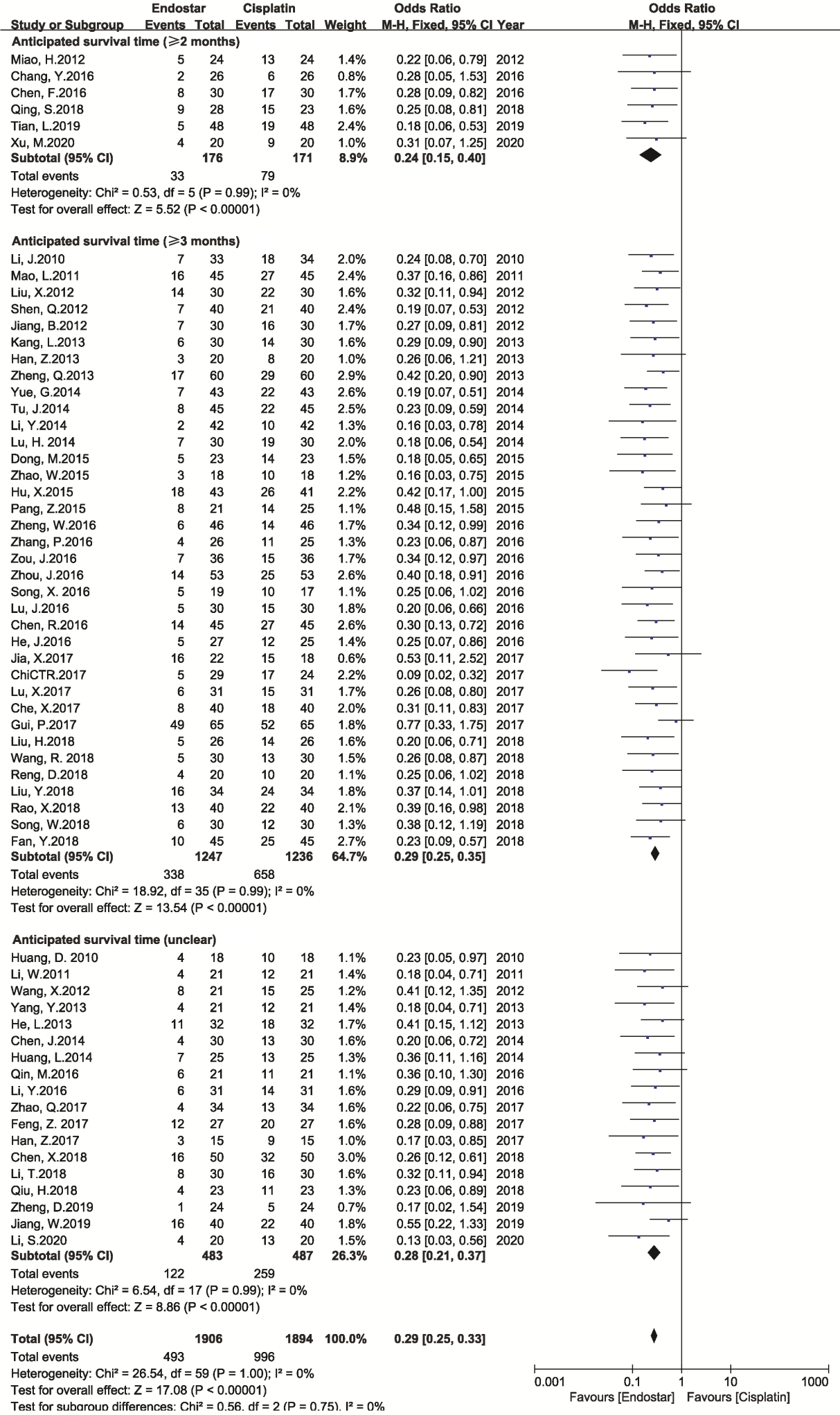


Figure S38.Subgroups analysis of treatment failure via anticipated survival time


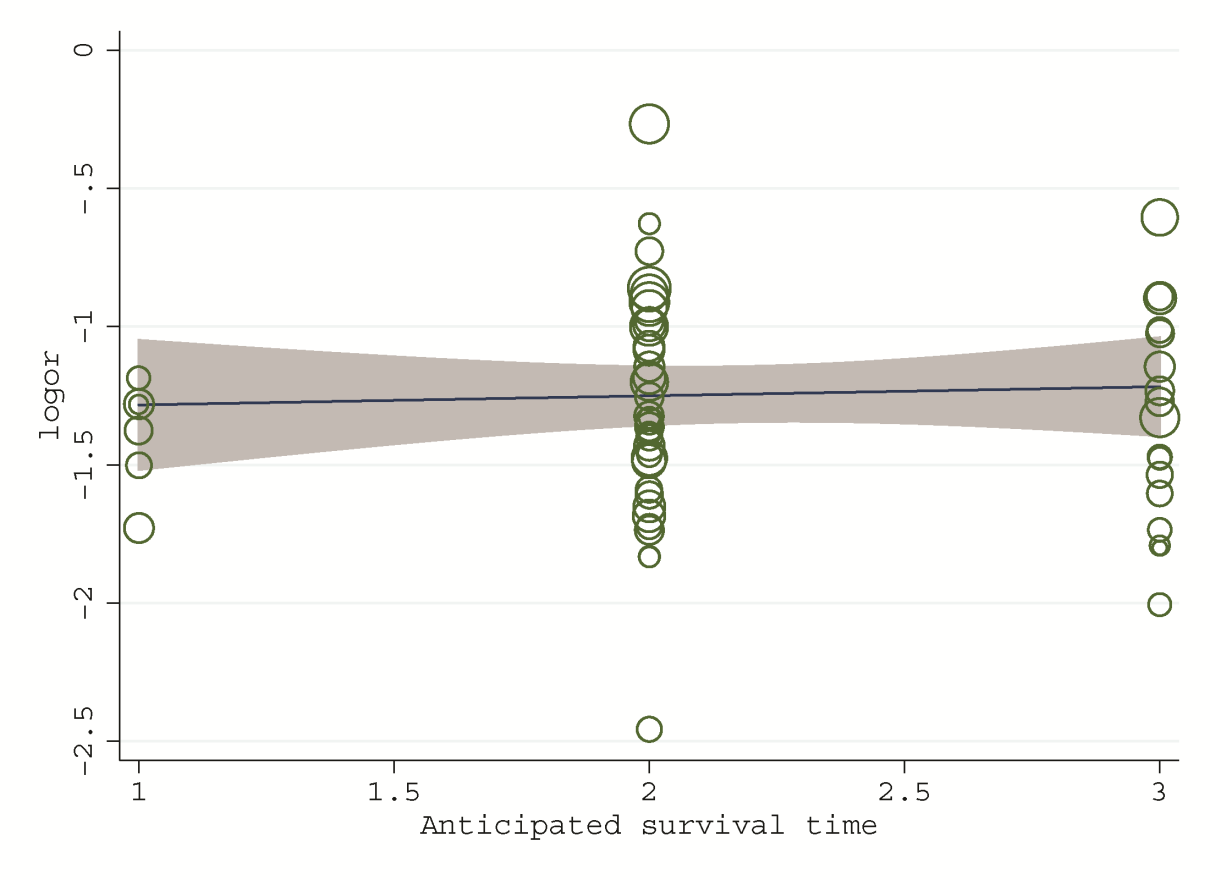


Figure S39. Meta regression of treatment failure via anticipated survival time


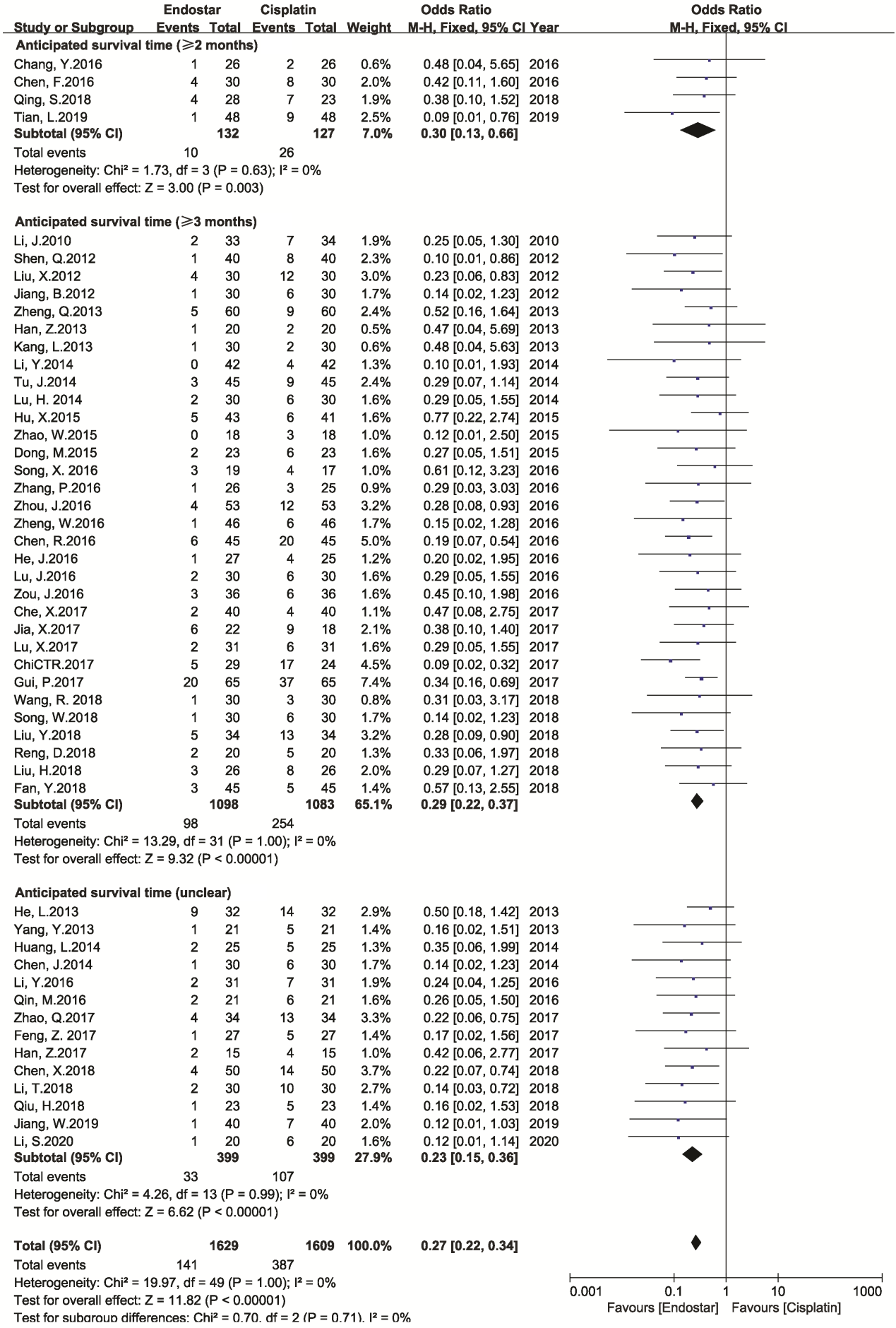


Figure S40. Subgroups analysis of treatment failure via anticipated survival time


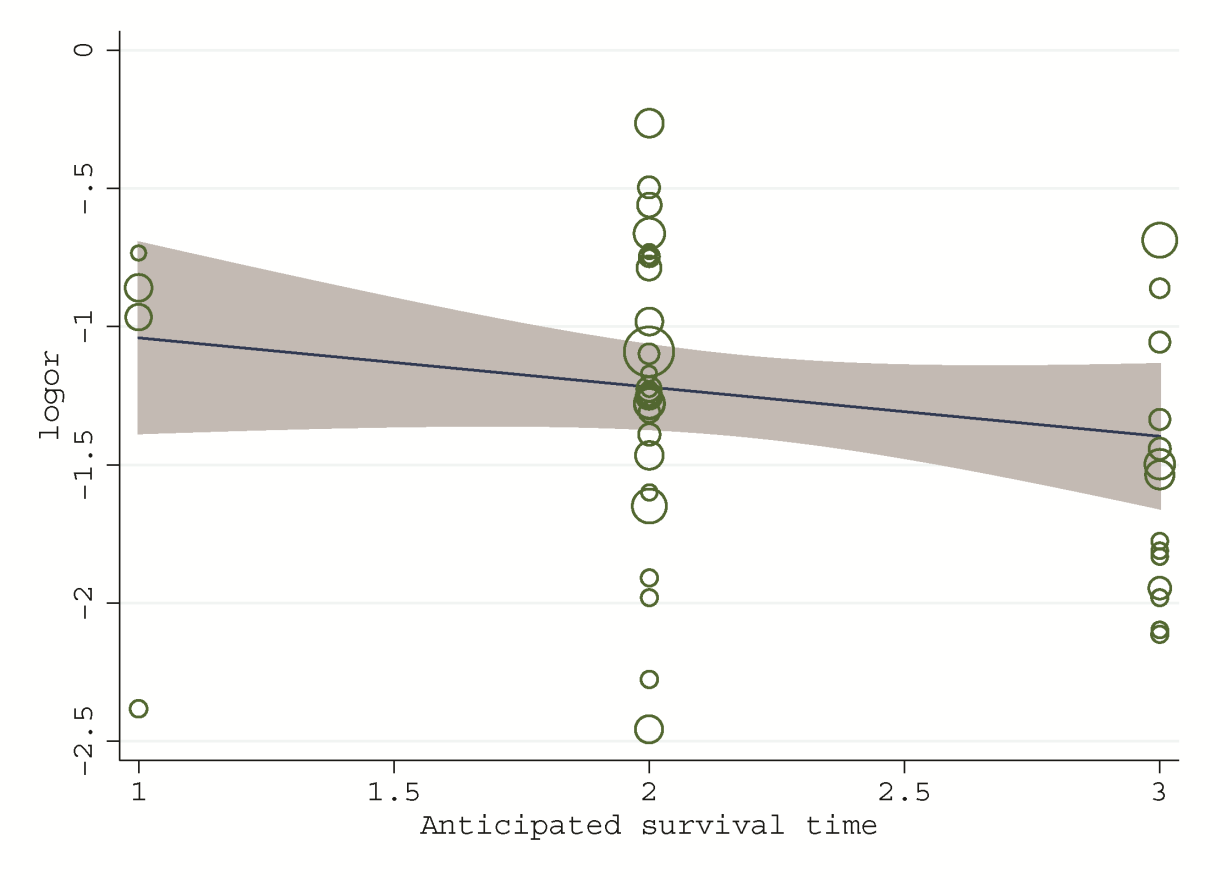


Figure S41. Meta regression of treatment failure via anticipated survival time
