# Supplementary material for: Intrapleural Administration With Rh-Endostatin and Chemical Irritants in the Control of Malignant Pleural Effusion: A Systematic Review and Meta-Analysis
Source: Front Oncol. 2021 Aug 3;11:649999. doi: 10.3389/fonc.2021.649999 (PMC8369576; doi:10.3389/fonc.2021.649999)
Supplement: Supplementary file 1 [file DataSheet_1.zip › Supplementary Material 6B.docx]

**Supplementary Material.6B Figures.S42-71 Subgroups analysis and meta-regression**


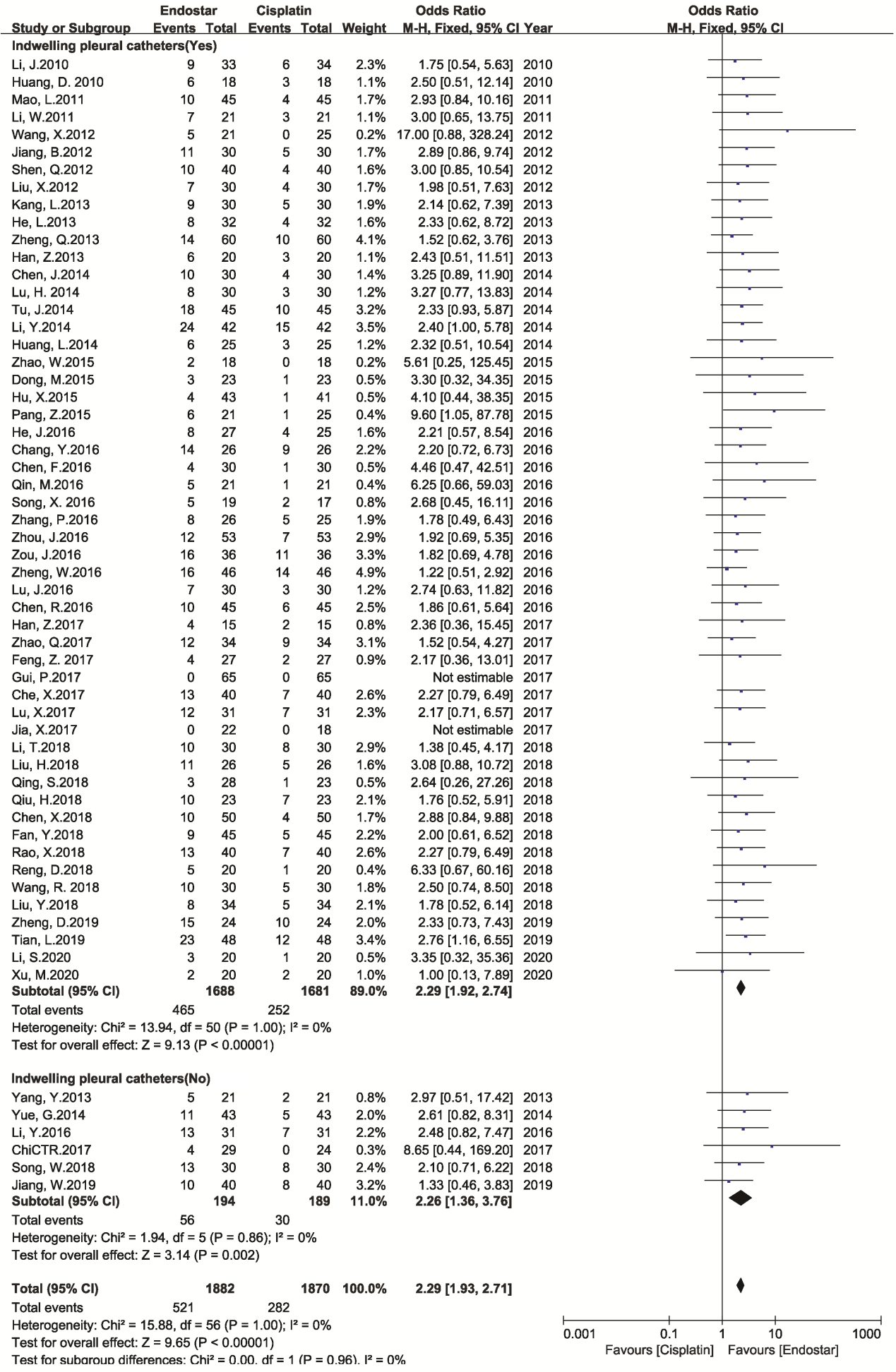


Figure S42. Subgroups analysis of complete response via indwelling pleural catheters


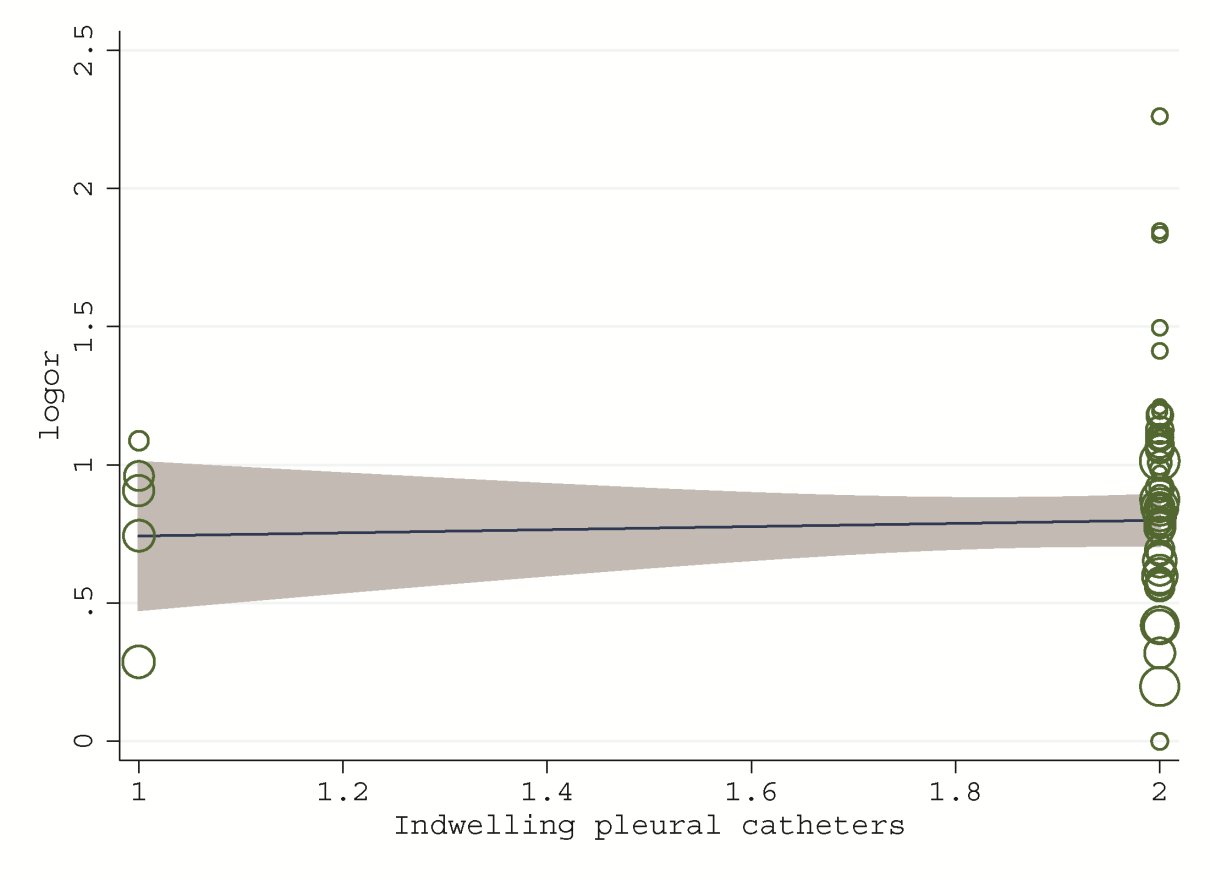


Figure S43. Meta regression of complete response via indwelling pleural catheters


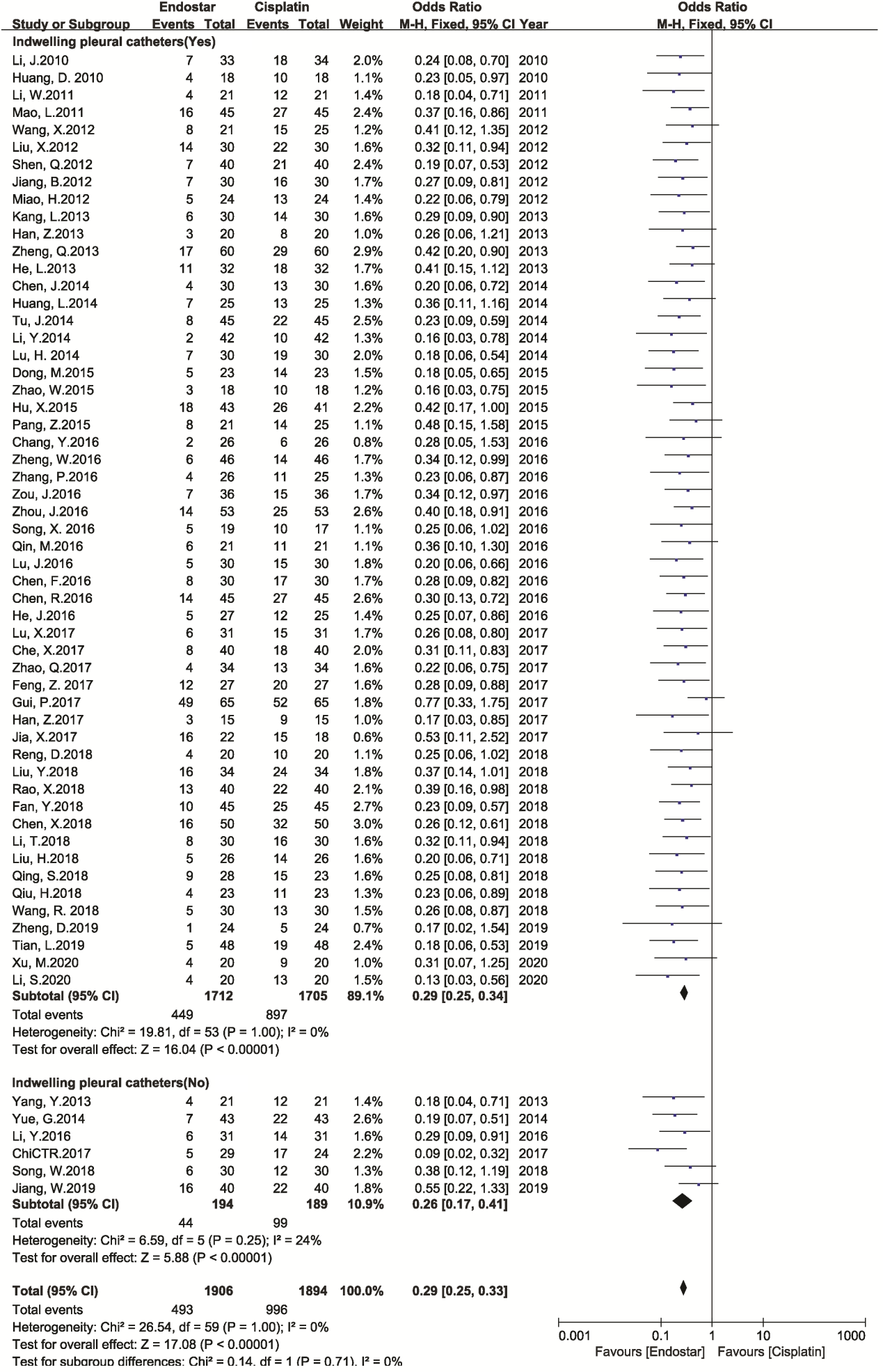


Figure S44. Subgroups analysis of treatment failure via indwelling pleural catheters


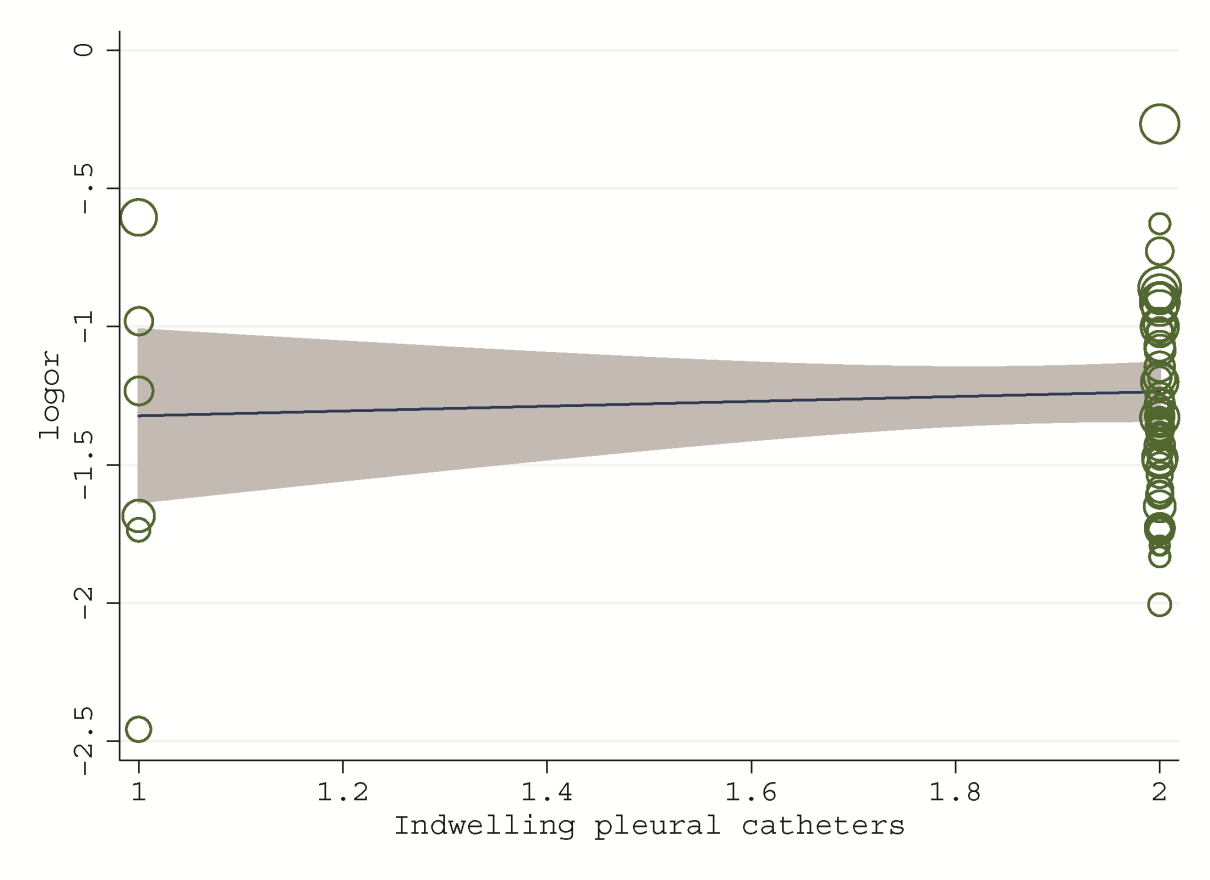


Figure S45. Meta regression of treatment failure via indwelling pleural catheters


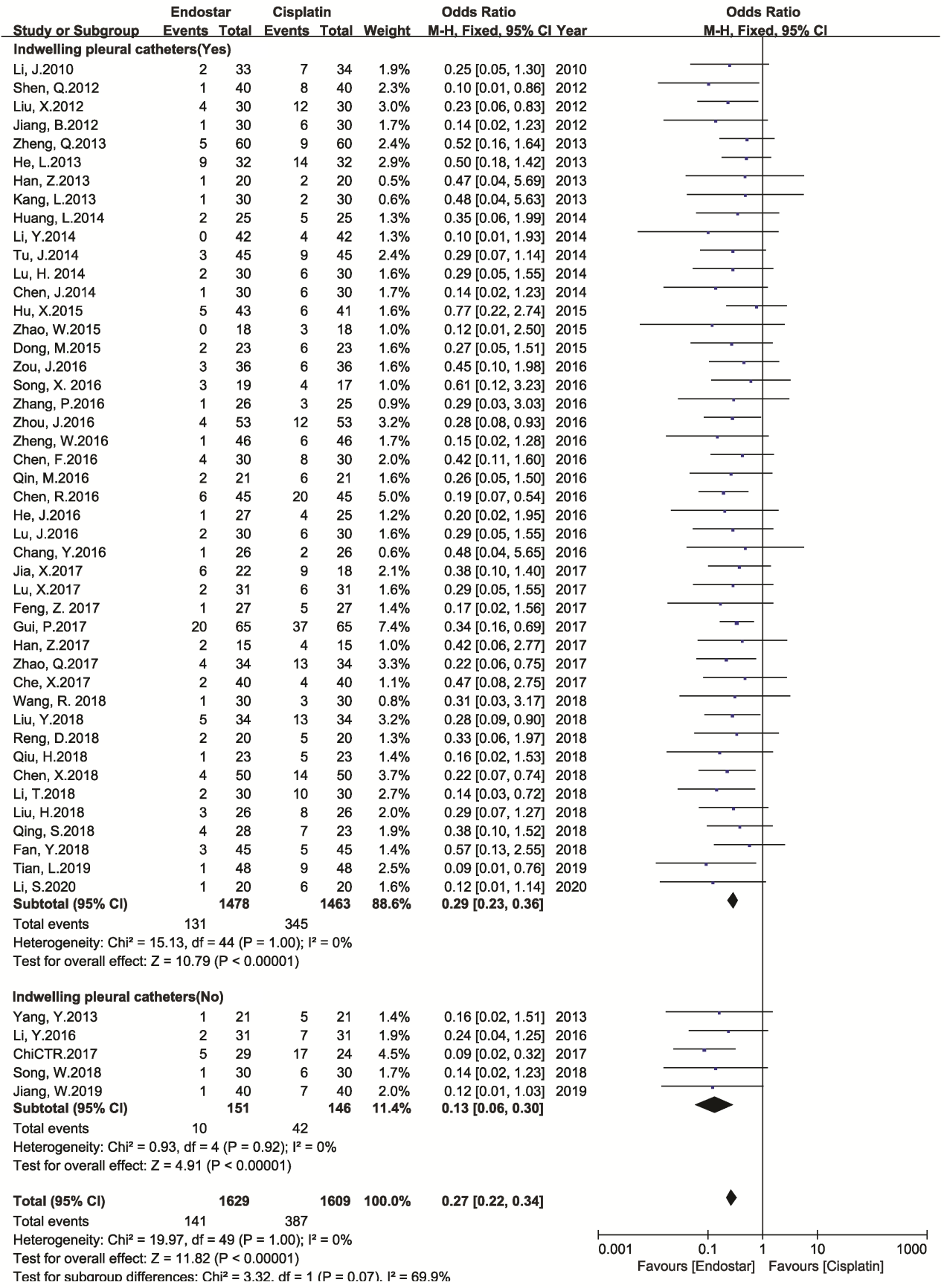


Figure S46. Subgroups analysis of treatment failure via indwelling pleural catheters


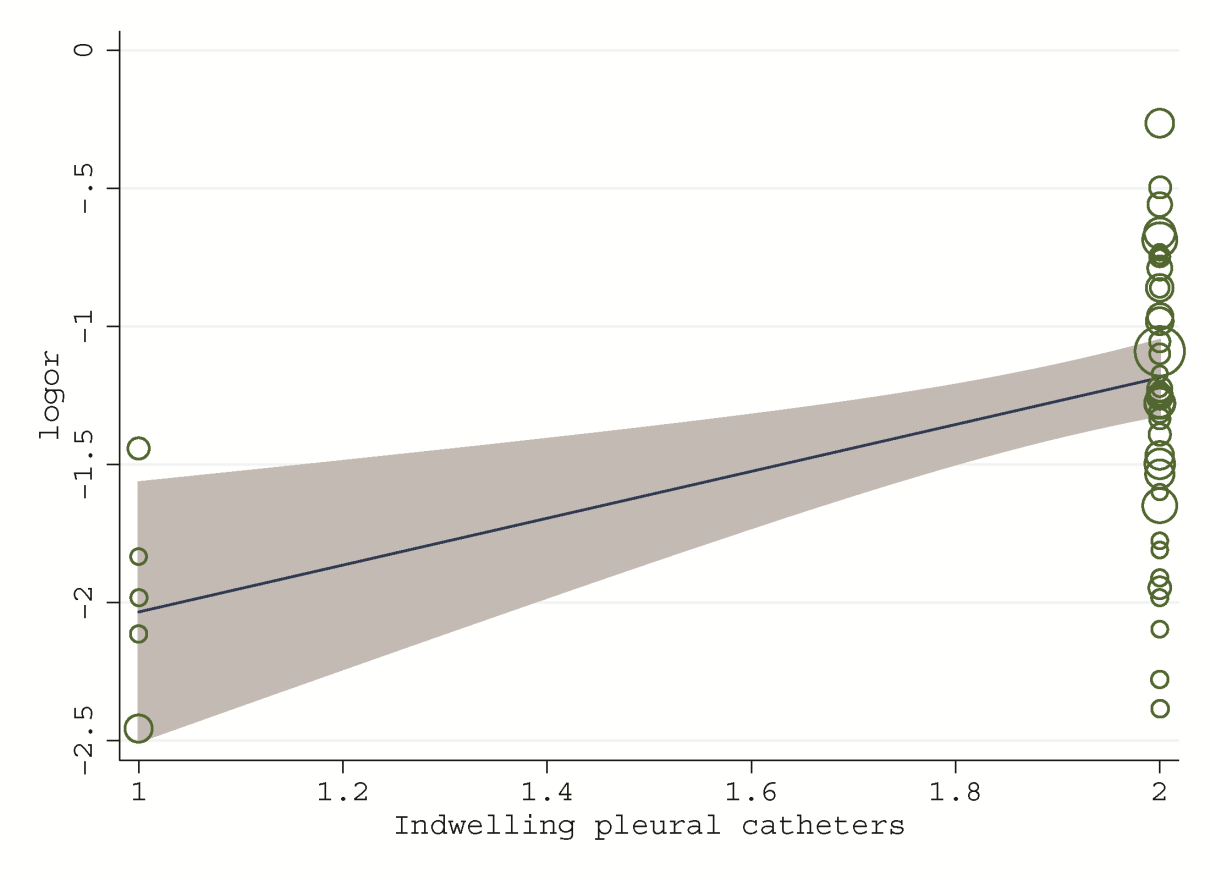


Figure S47. Meta regression of treatment failure via indwelling pleural catheters


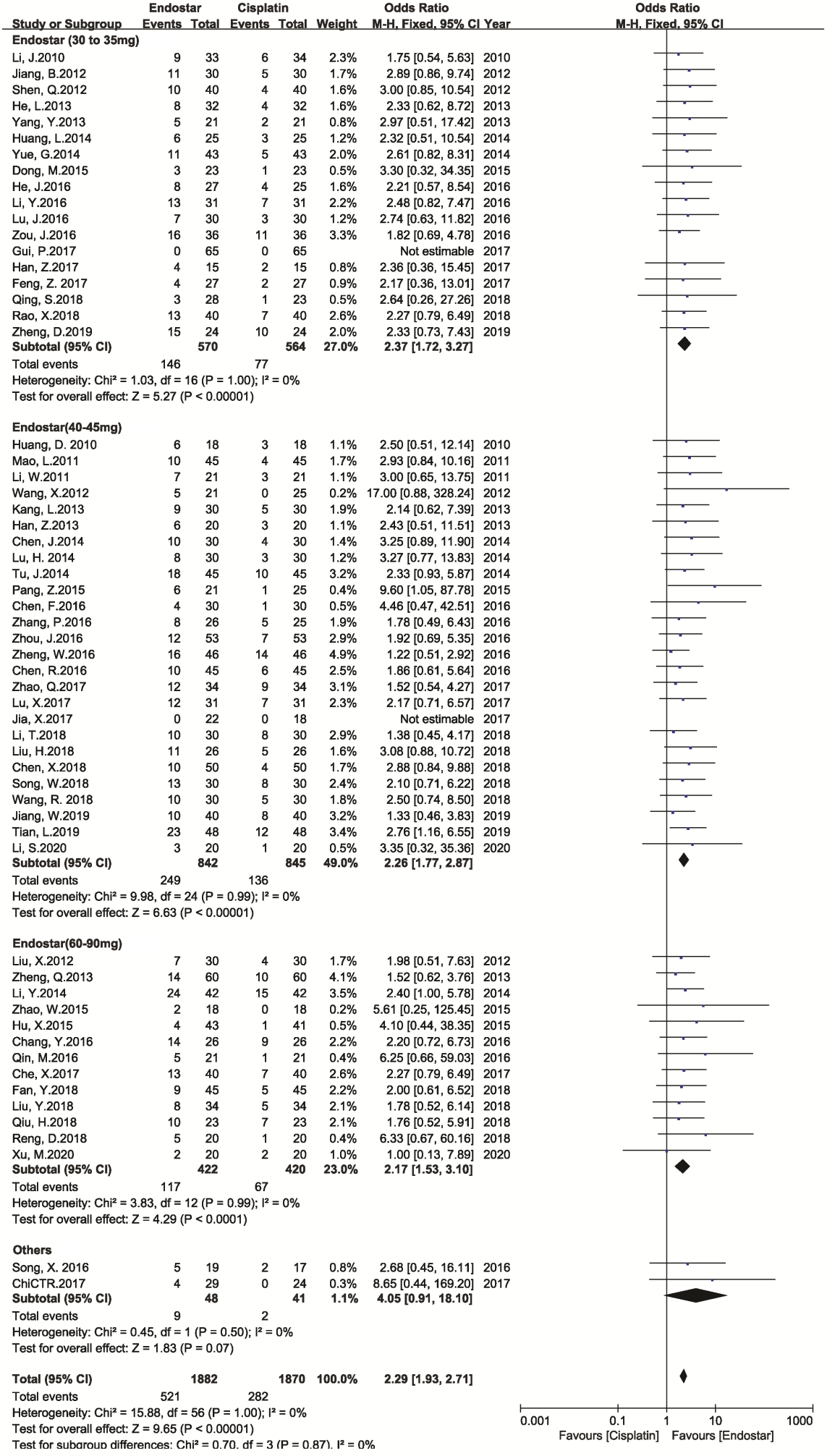
Figure S48. Subgroups analysis of complete response via Endostar dosage


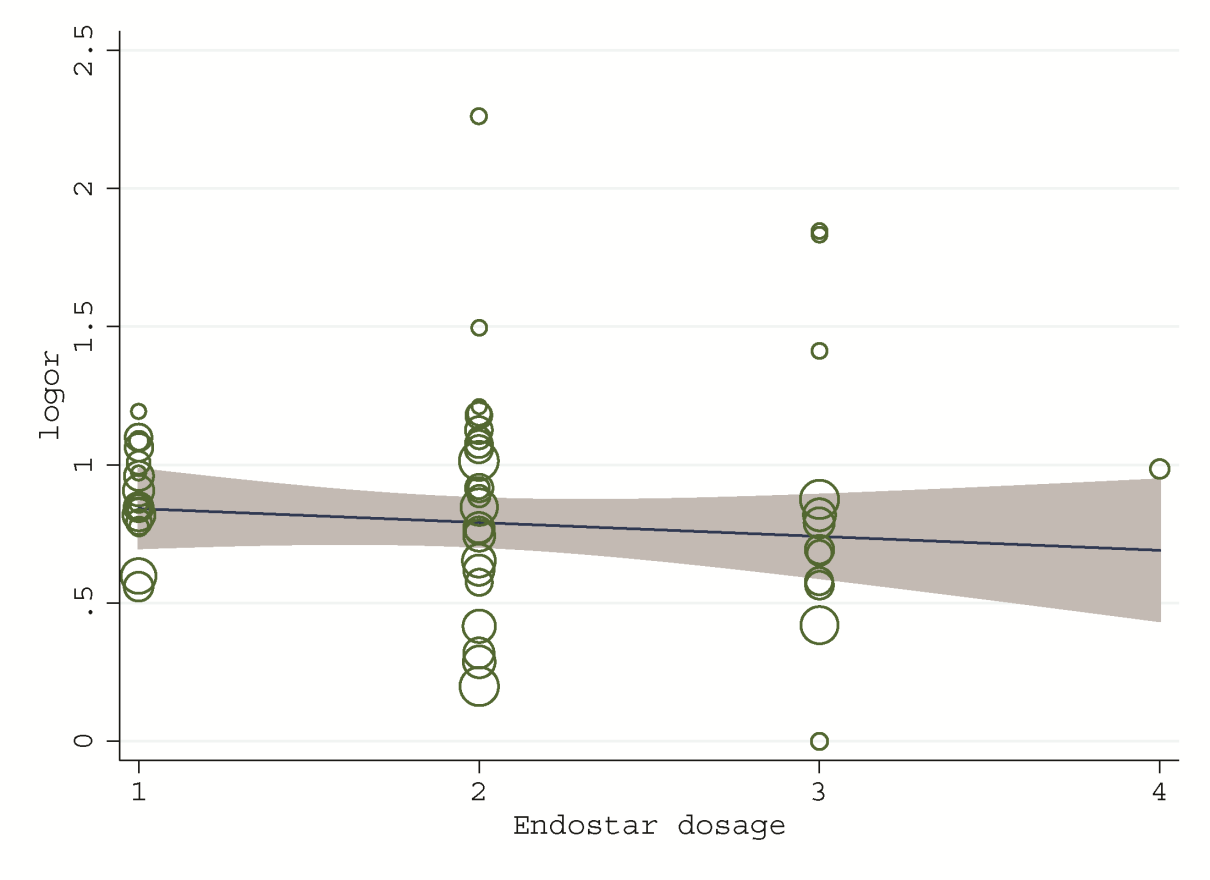


Figure S49. Meta regression of complete response via Endostar dosage


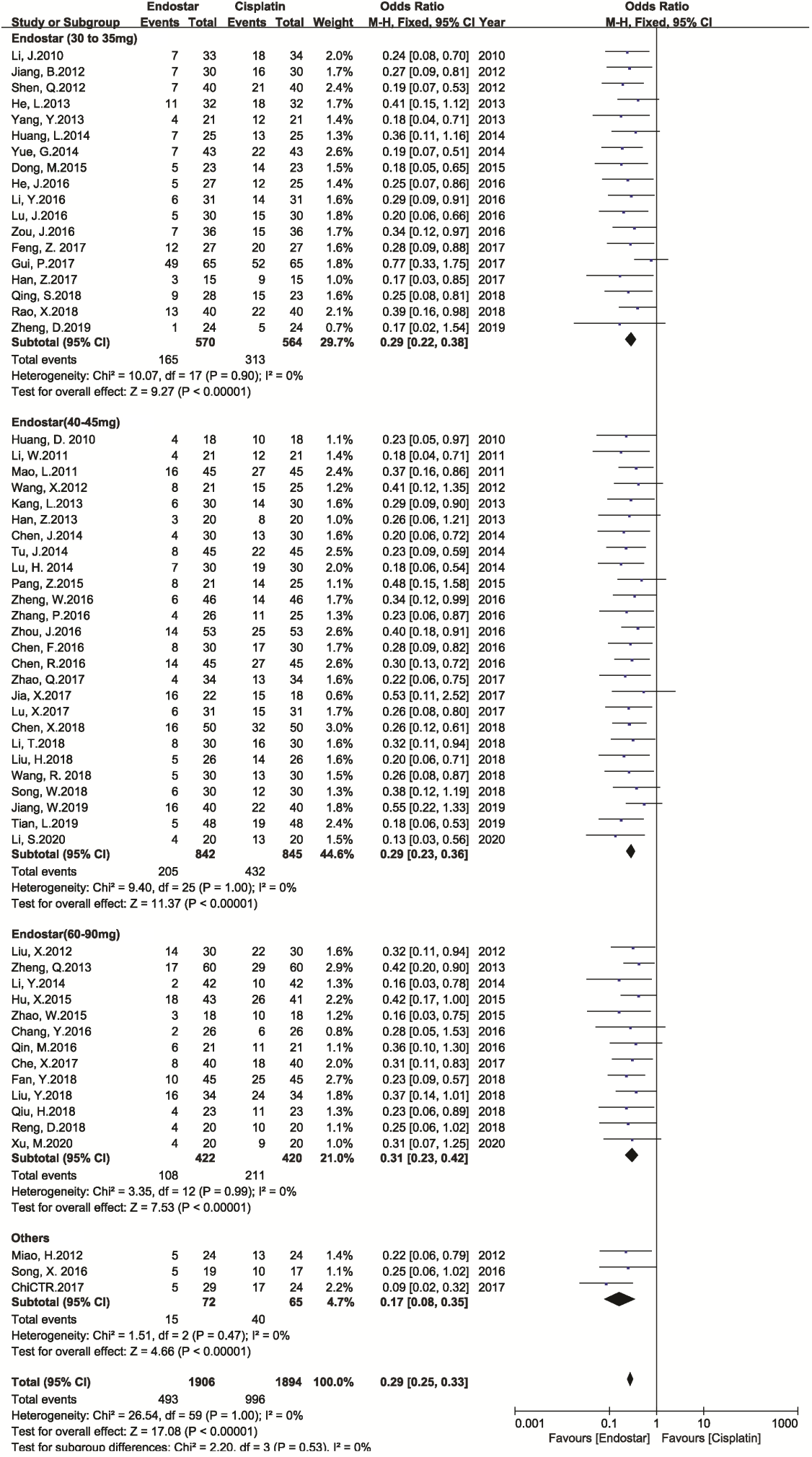


Figure S50. Subgroups analysis of treatment failure via Endostar dosage


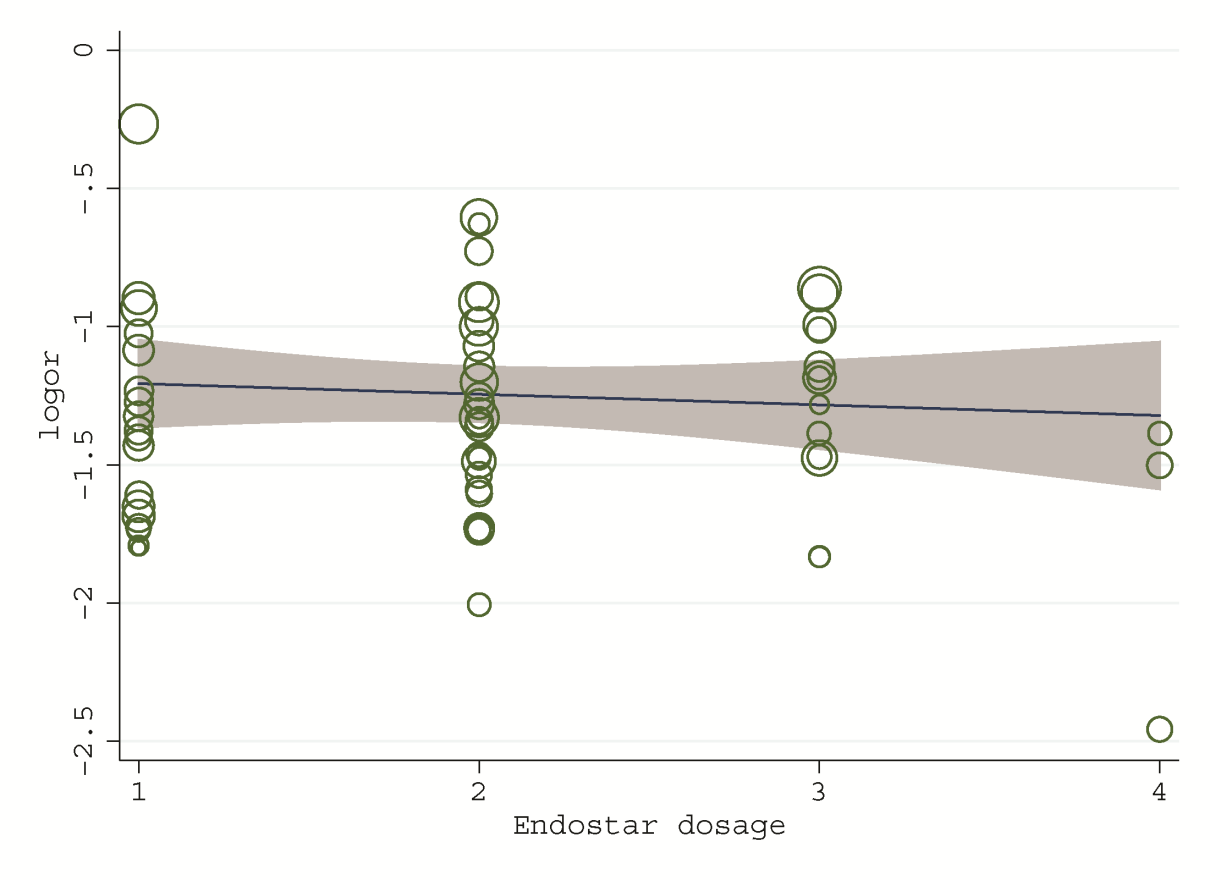


Figure S51. Meta regression of treatment failure via Endostar dosage


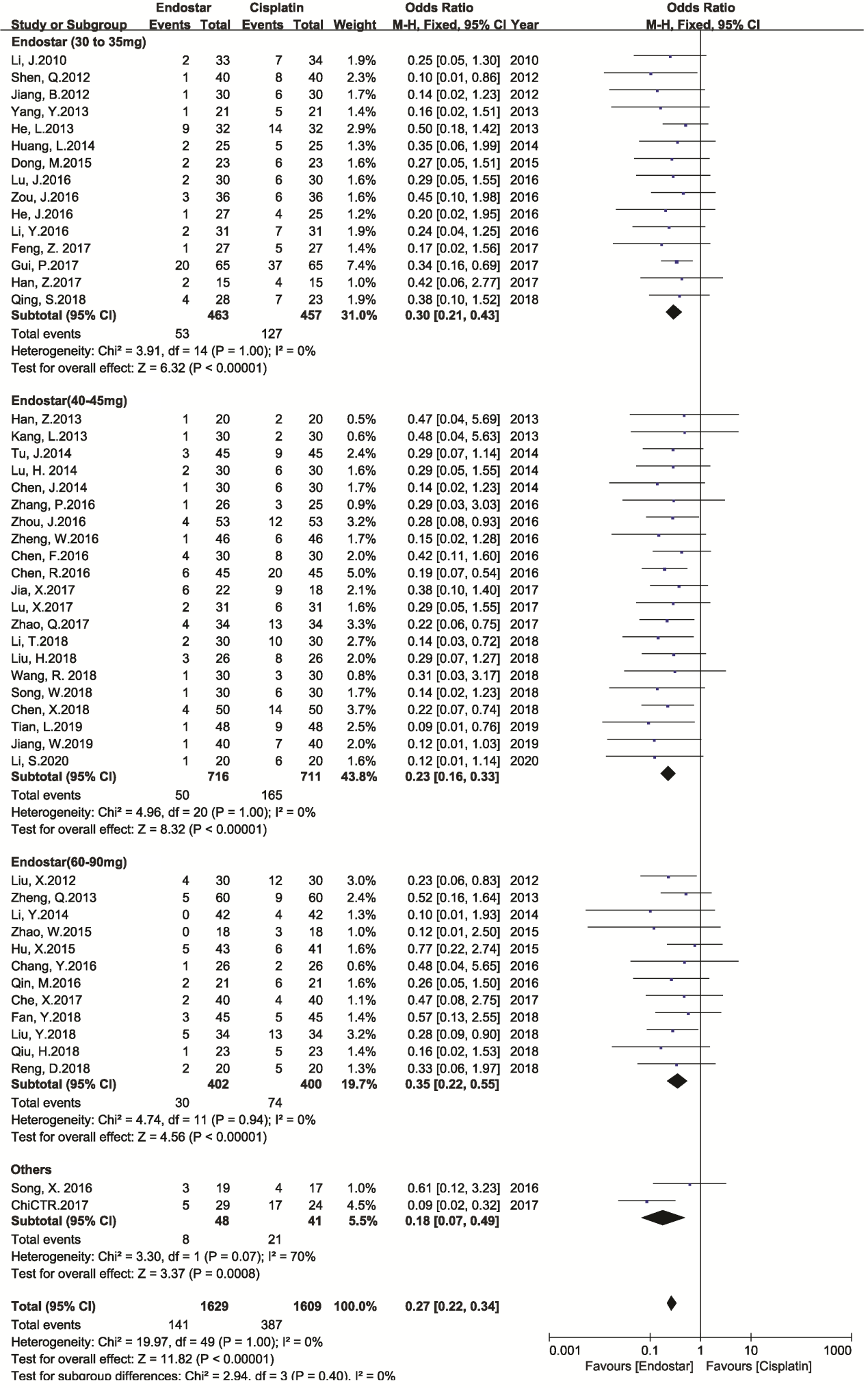


Figure S52. Subgroups analysis of treatment failure via Endostar dosage


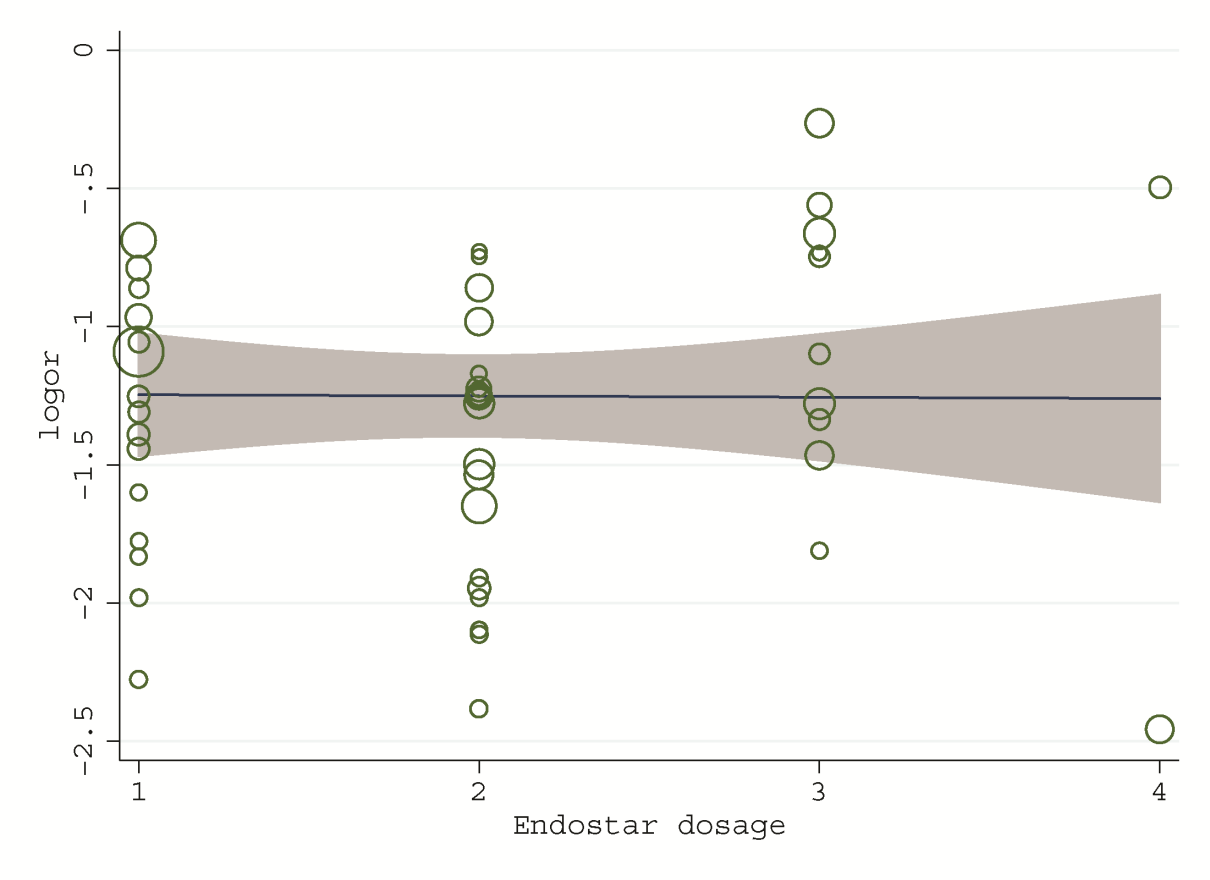


Figure S53. Meta regression of treatment failure via Endostar dosage


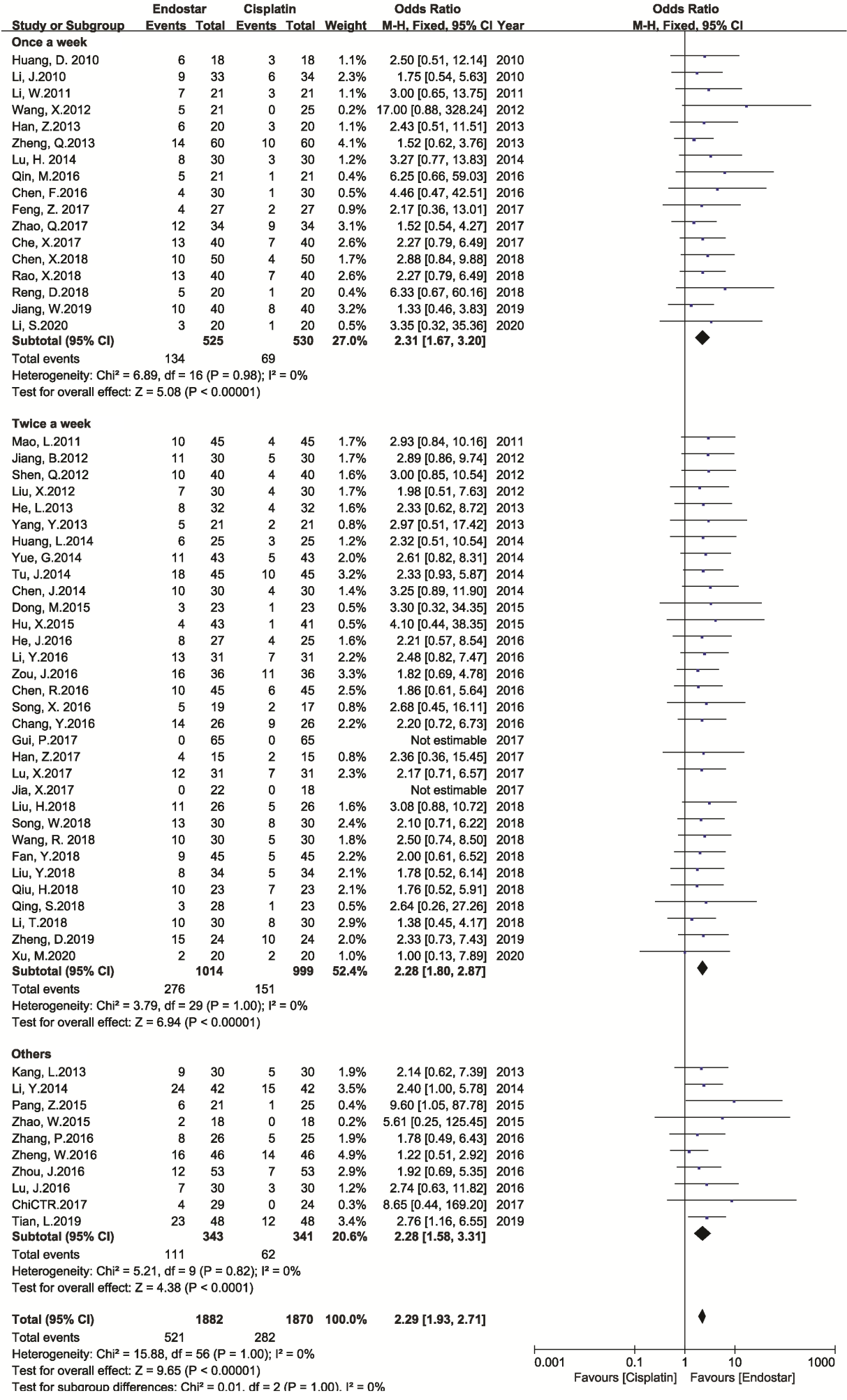
Figure S54. Subgroups analysis of complete response via treatment frequency


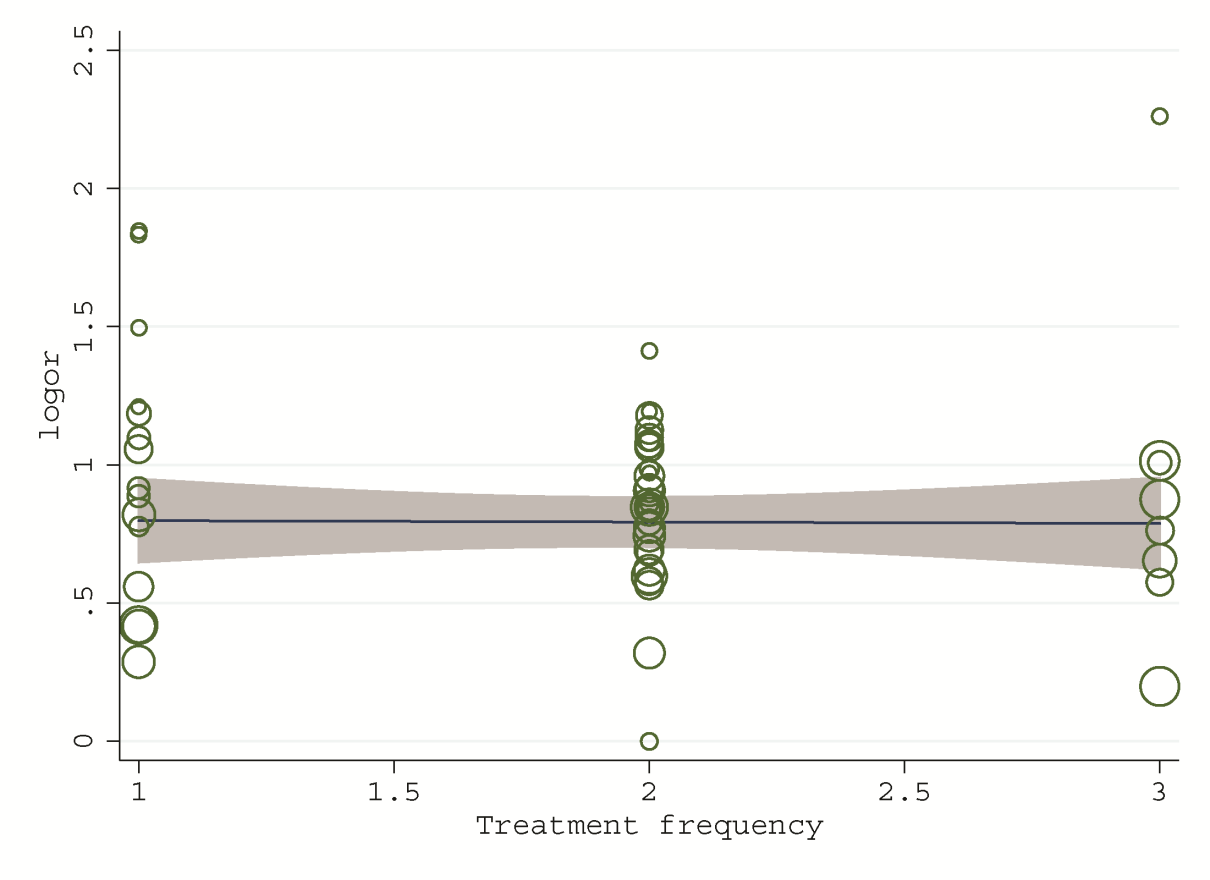


Figure S55. Meta regression of complete response via treatment frequency


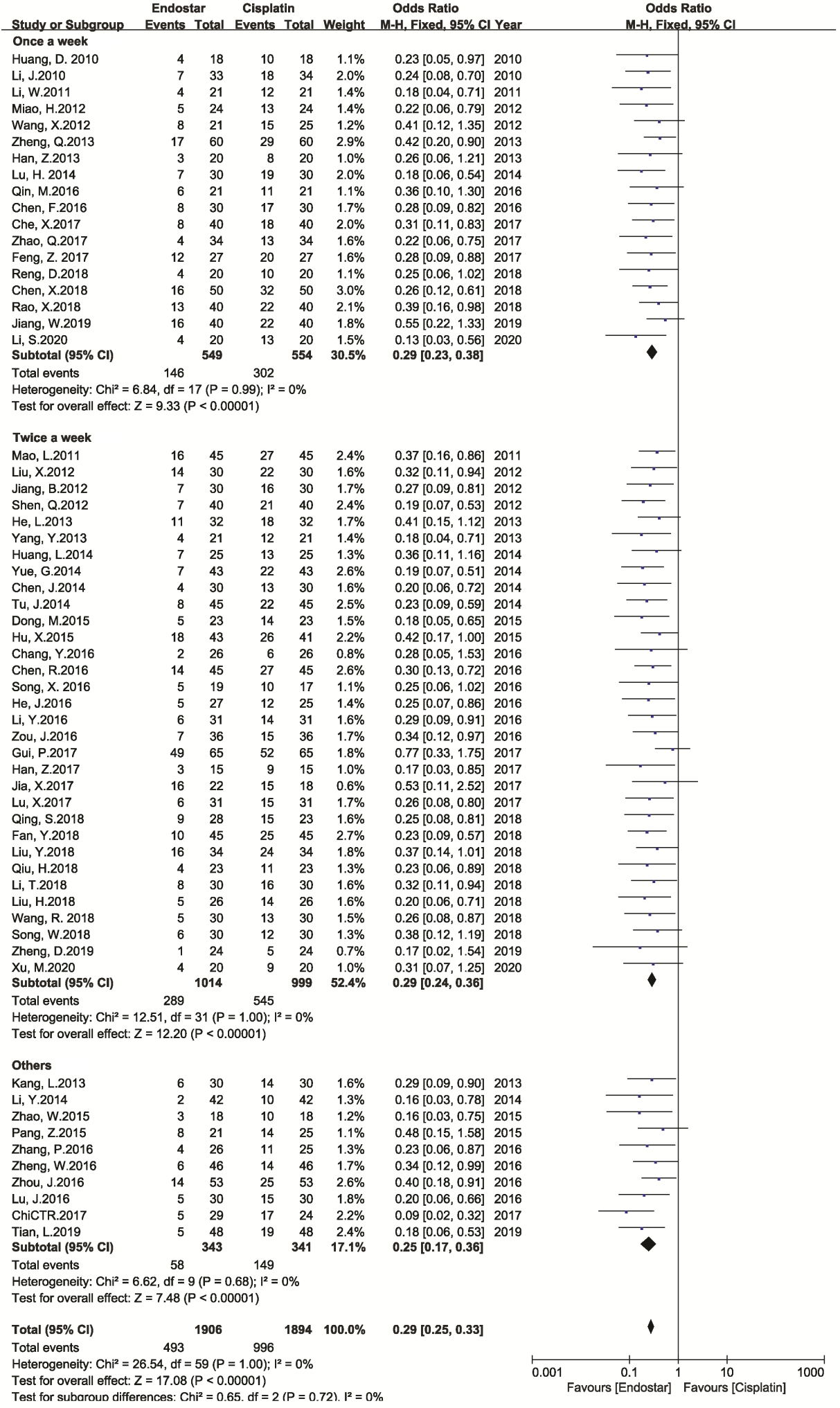


Figure S56. Subgroups analysis of treatment failure via treatment frequency


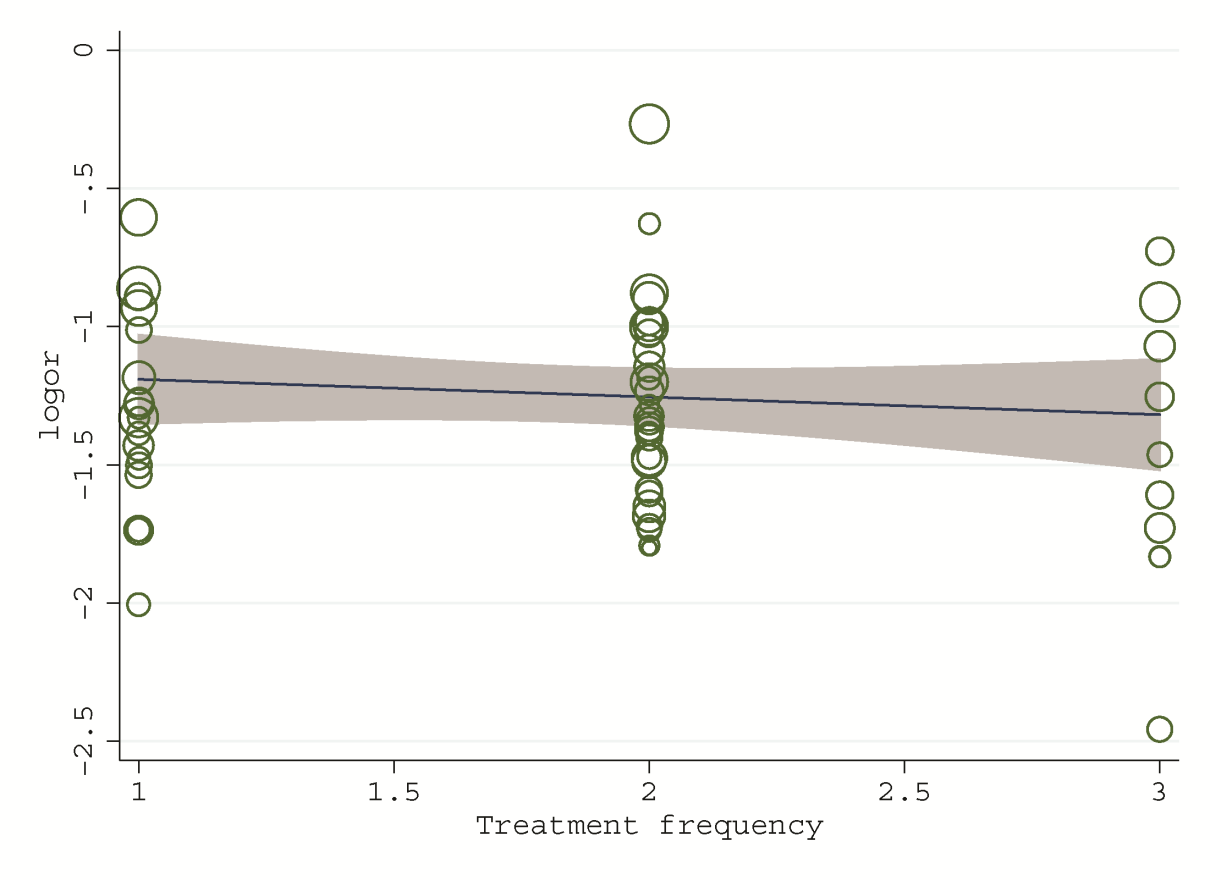


Figure S57. Meta regression of treatment failure via treatment frequency


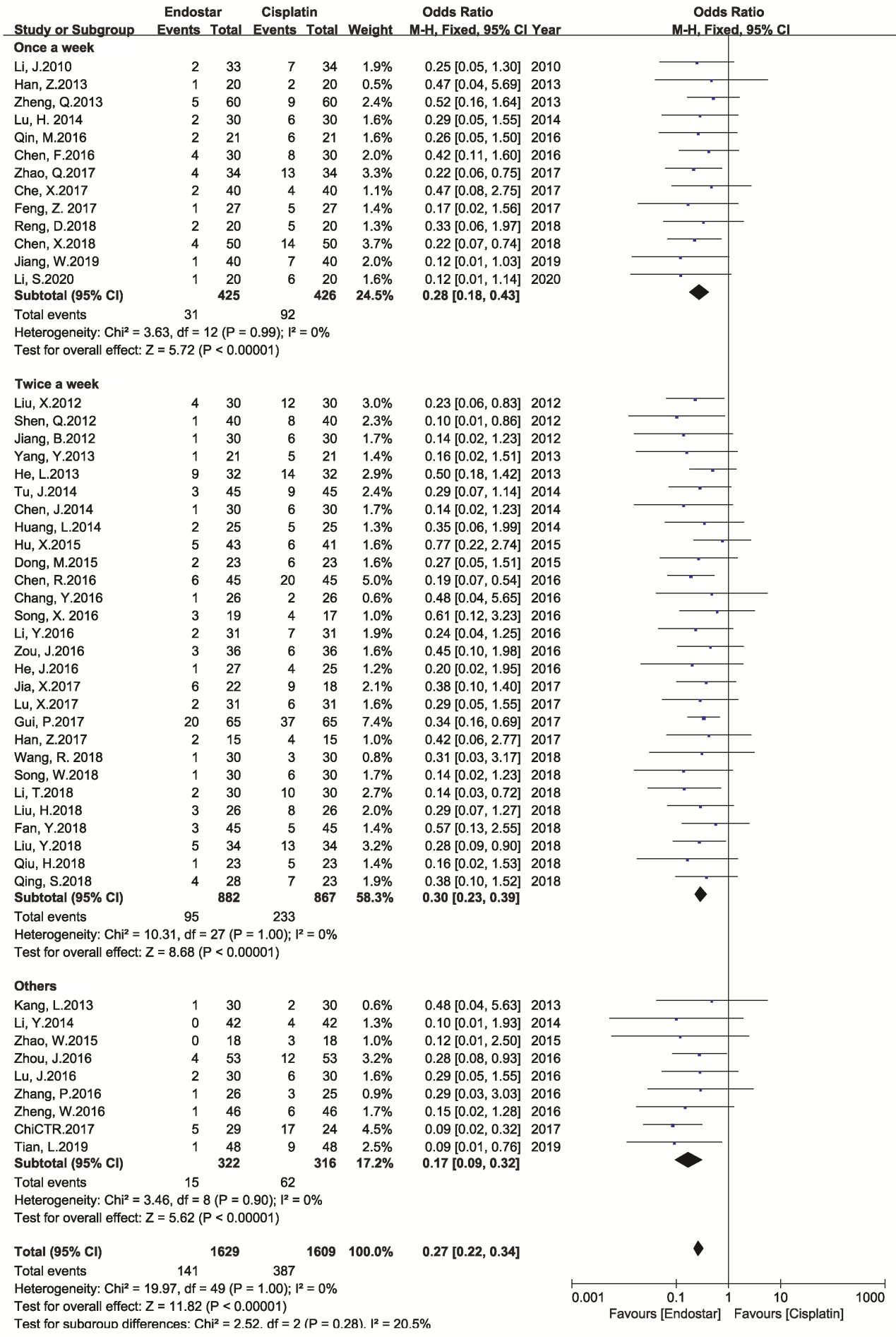


Figure S58. Subgroups analysis of treatment failure via treatment frequency


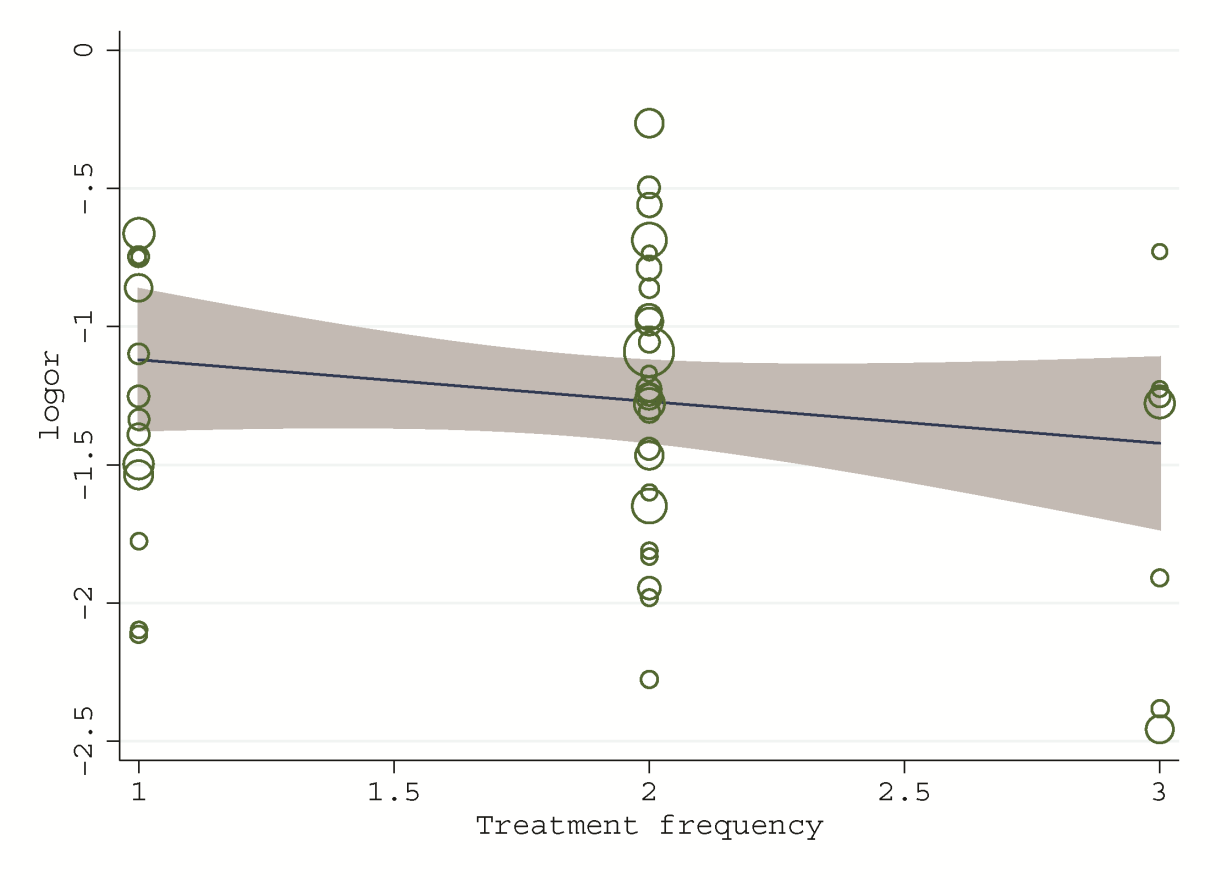


Figure S59. Meta regression of treatment failure via treatment frequency


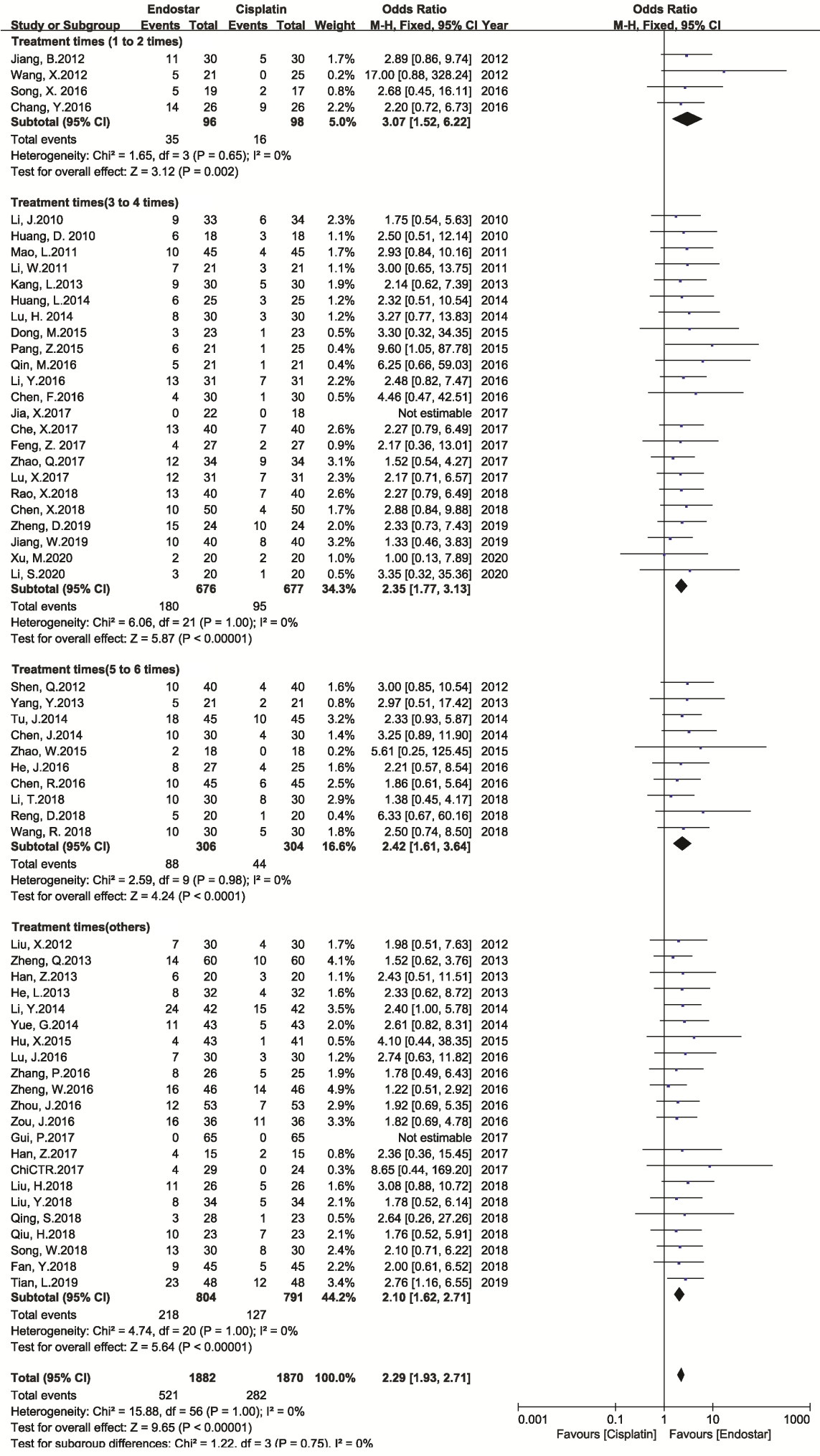


Figure S60. Subgroups analysis of complete response via treatment times


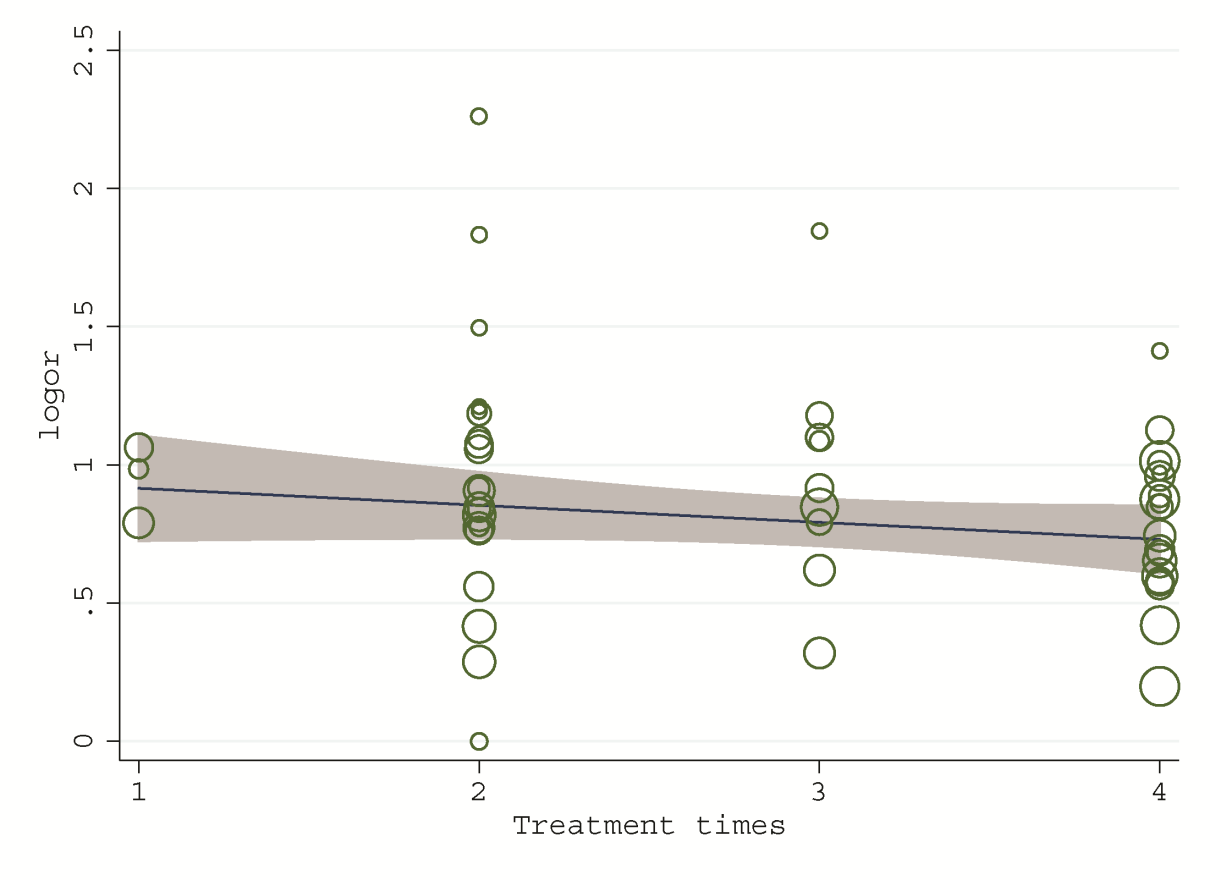


Figure S61. Meta regression of complete response via treatment times


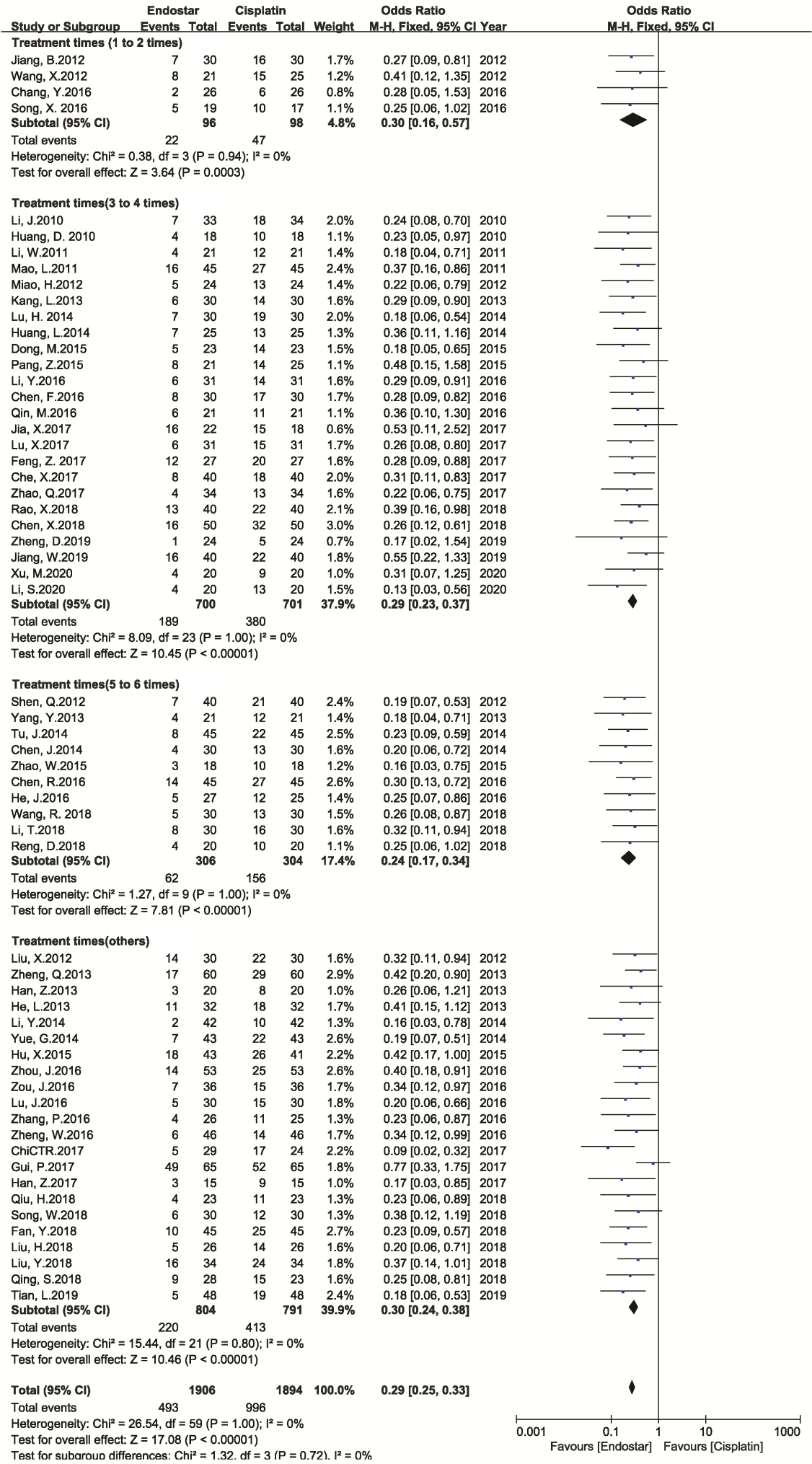


Figure S62. Subgroups analysis of treatment failure via treatment times


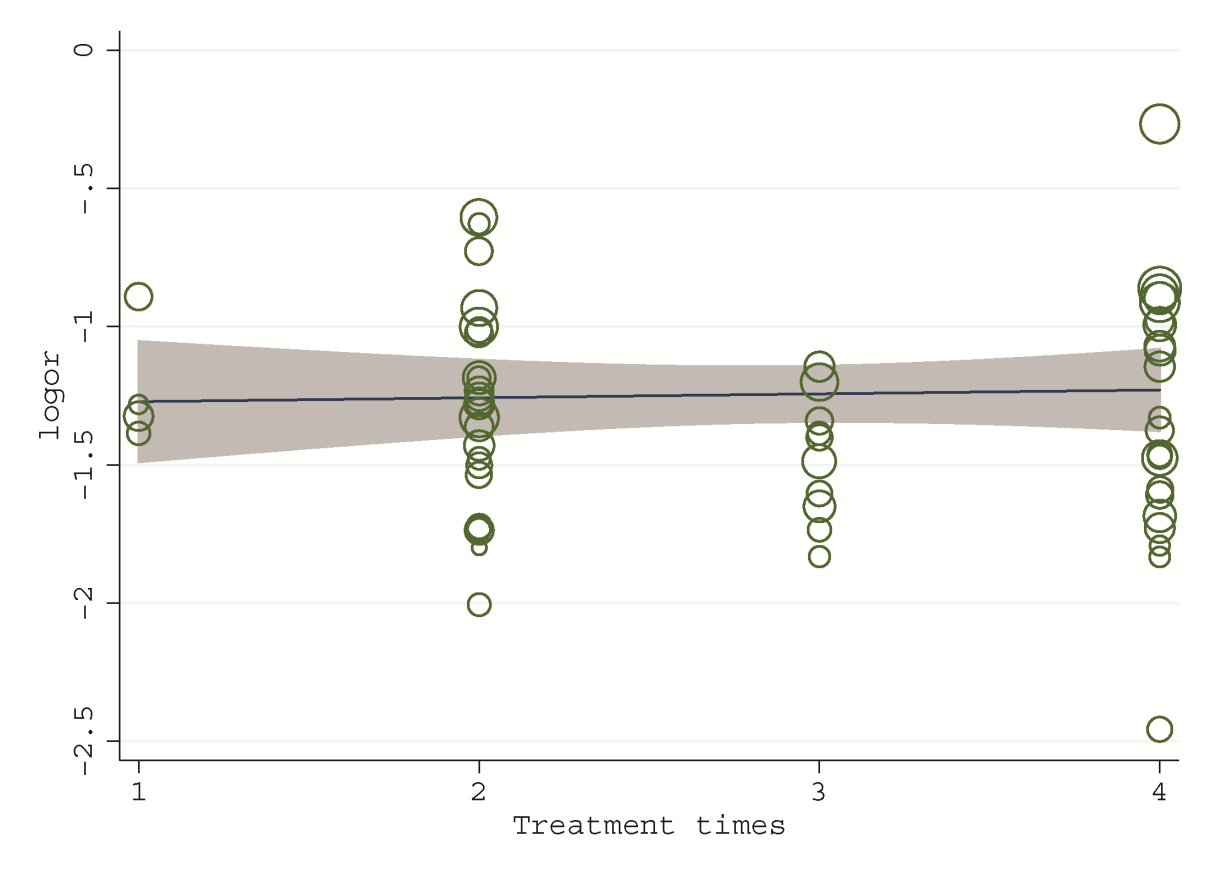


Figure S63. Meta regression of treatment failure via treatment times


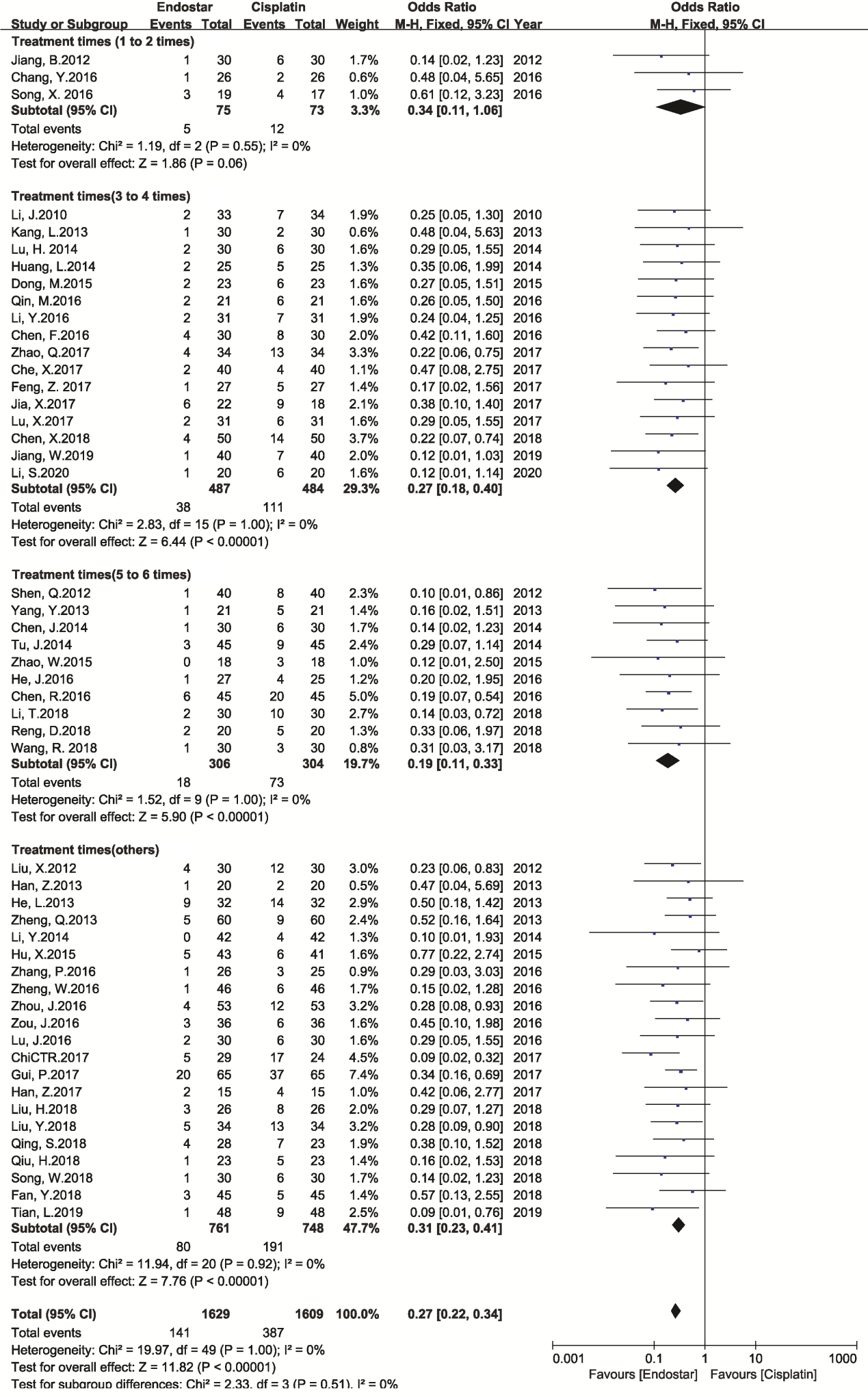


Figure S64. Subgroups analysis of treatment failure via treatment times


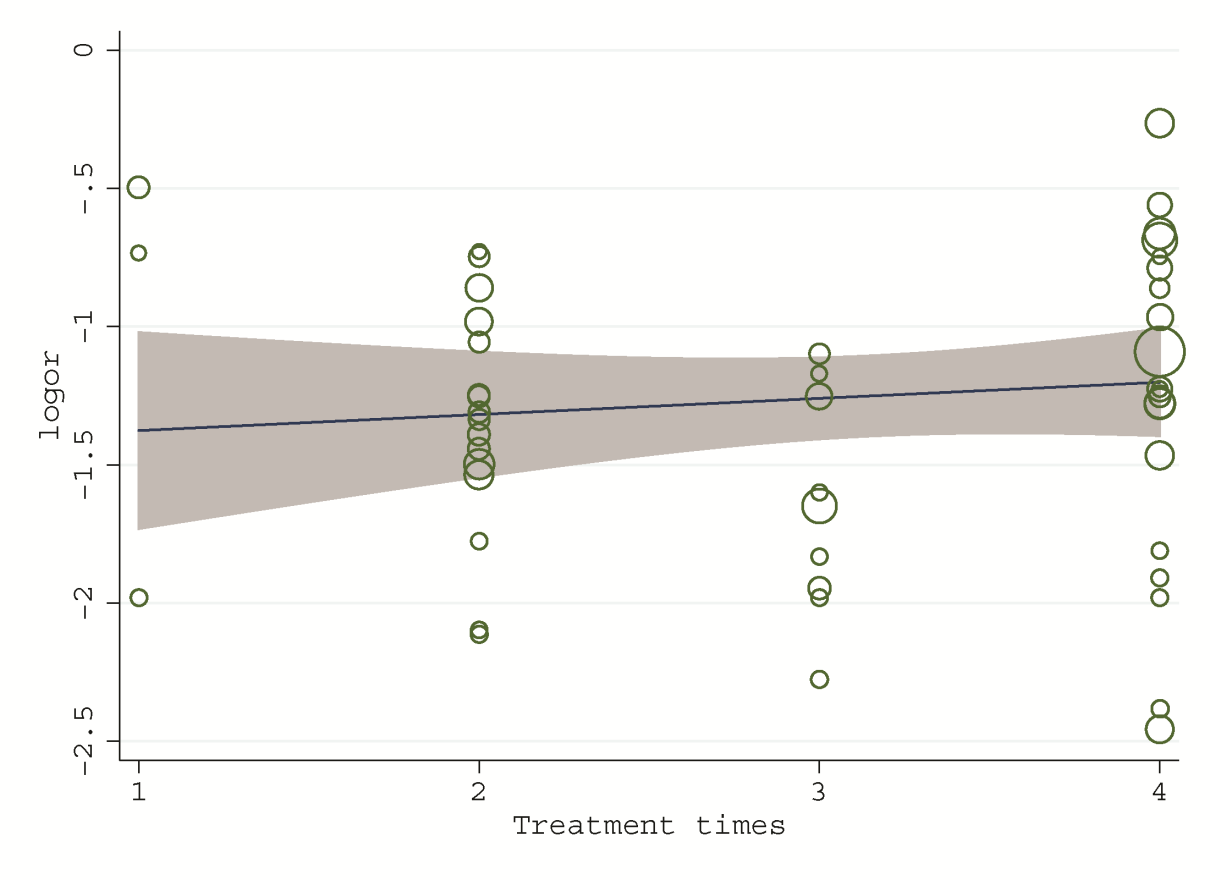


Figure S65. Meta regression of treatment failure via treatment times


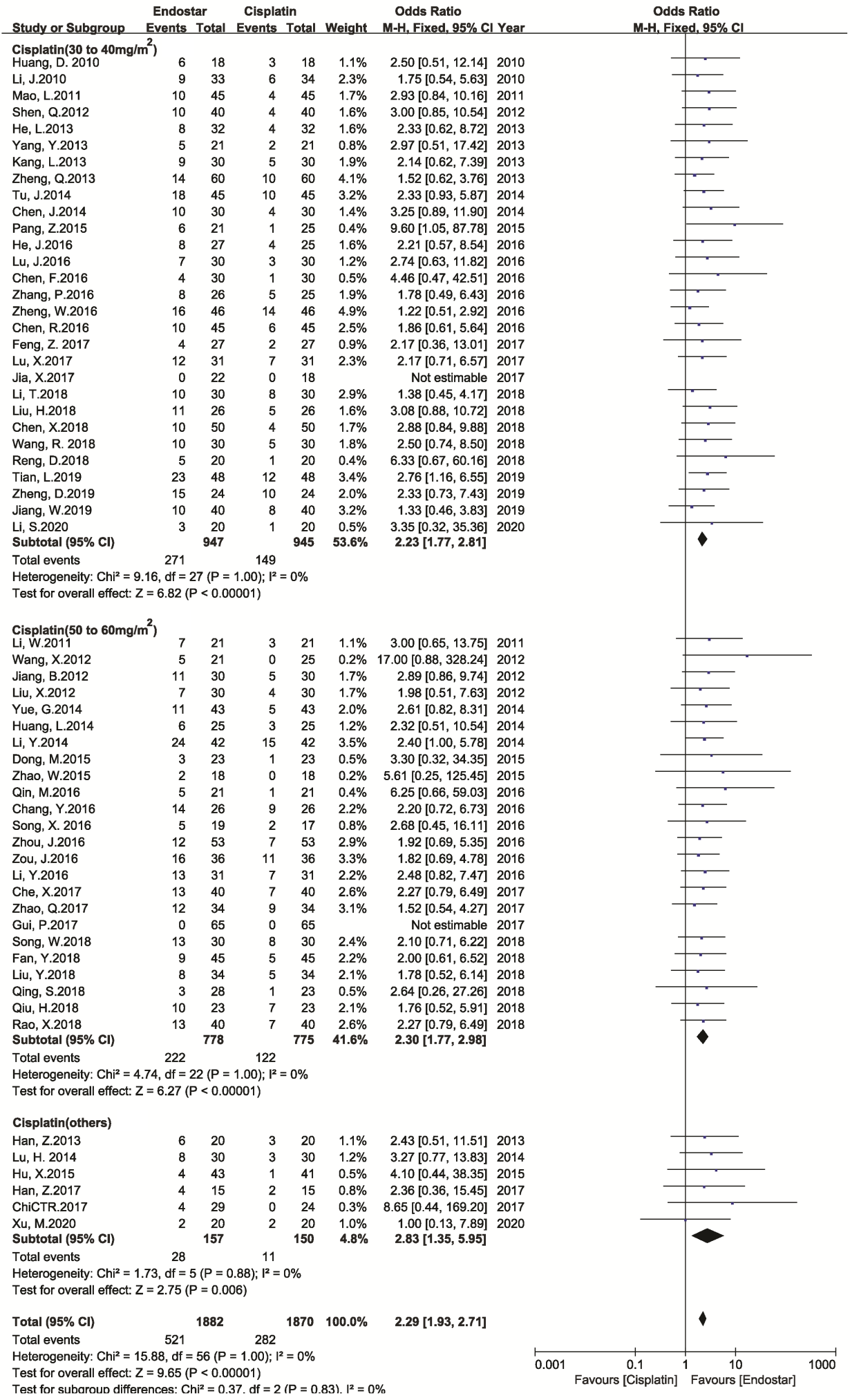
Figure S66. Subgroups analysis of complete response via DDP dosage


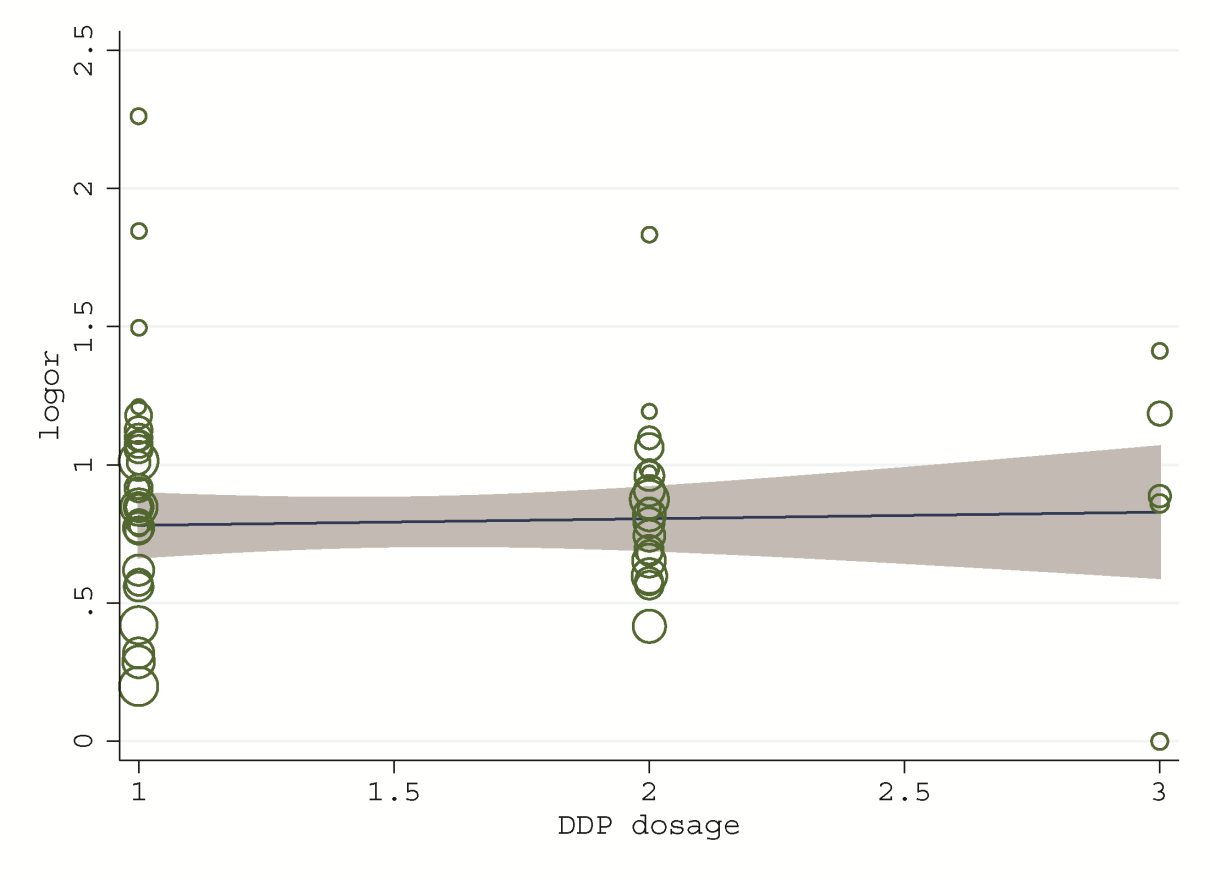


Figure S67. Meta regression of complete response via DDP dosage


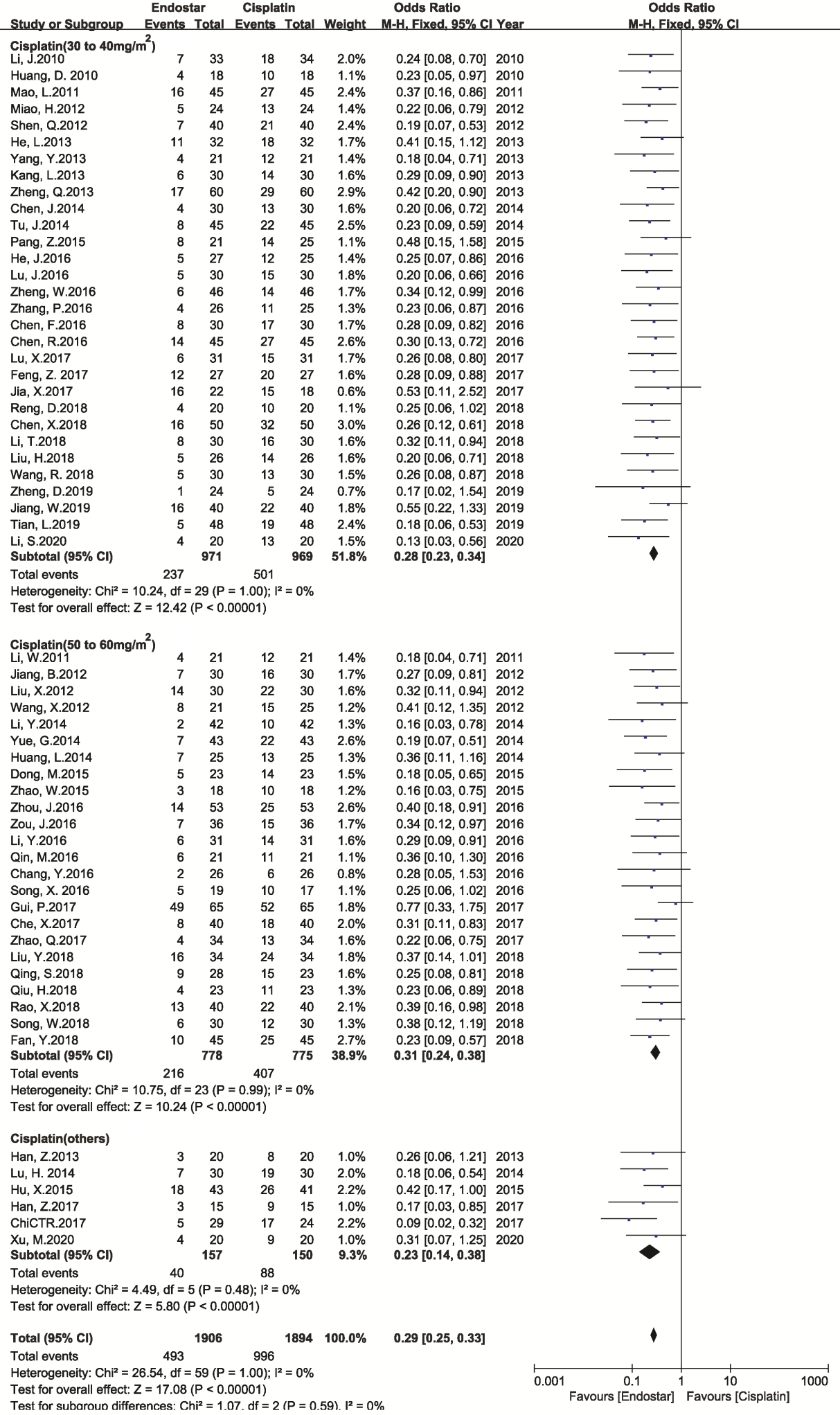


Figure S68. Subgroups analysis of treatment failure via DDP dosage


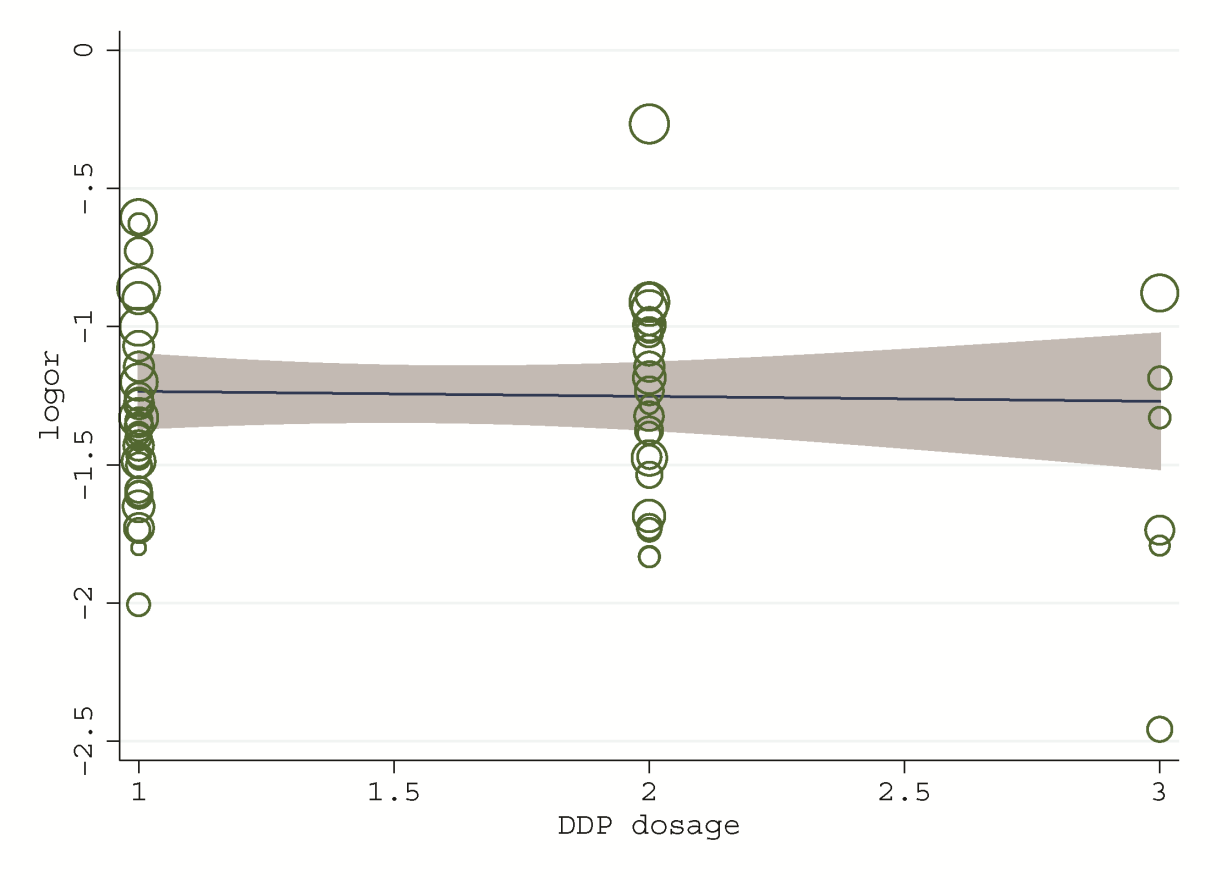


Figure S69. Meta regression of treatment failure via DDP dosage


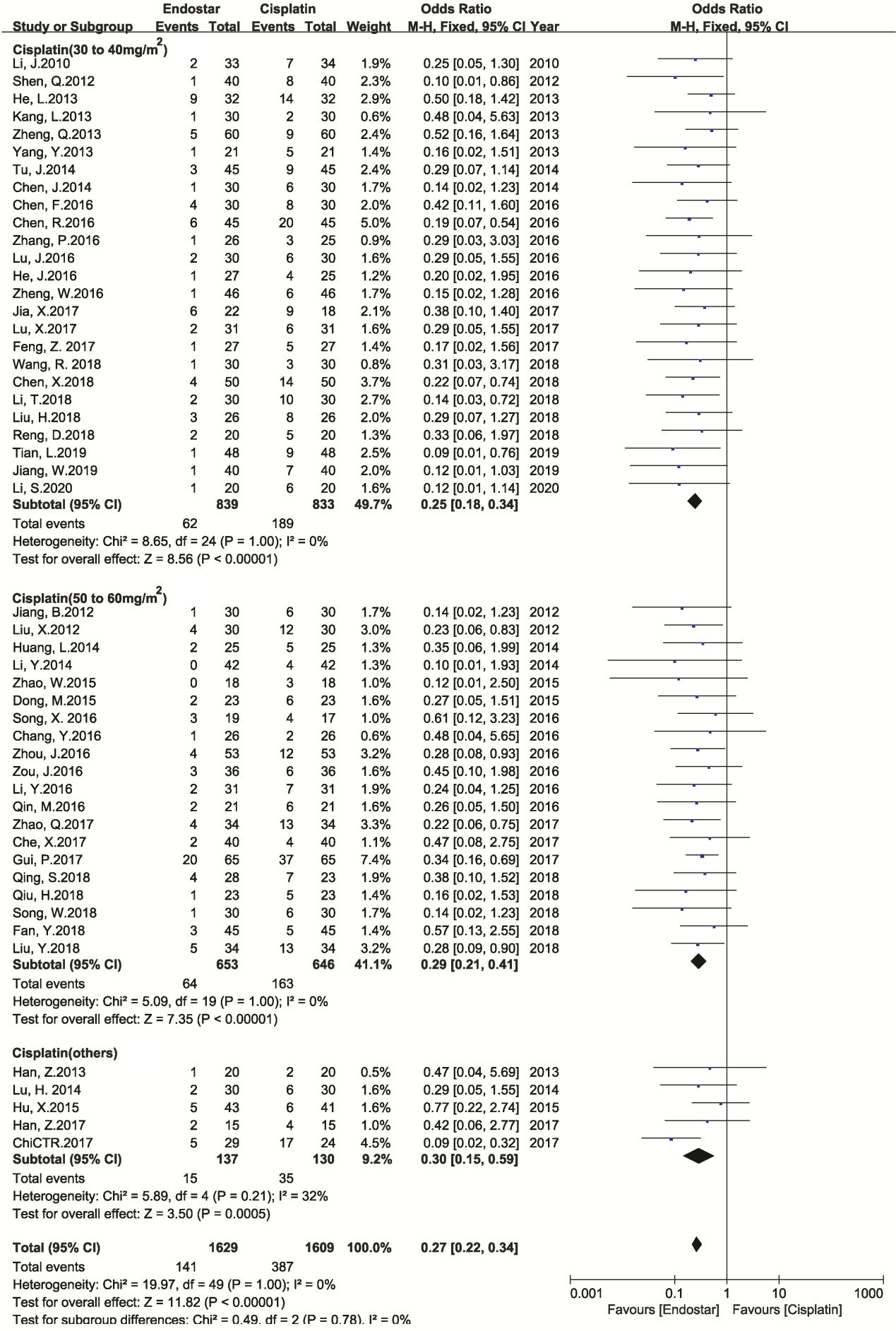


Figure S70. Subgroups analysis of treatment failure via DDP dosage


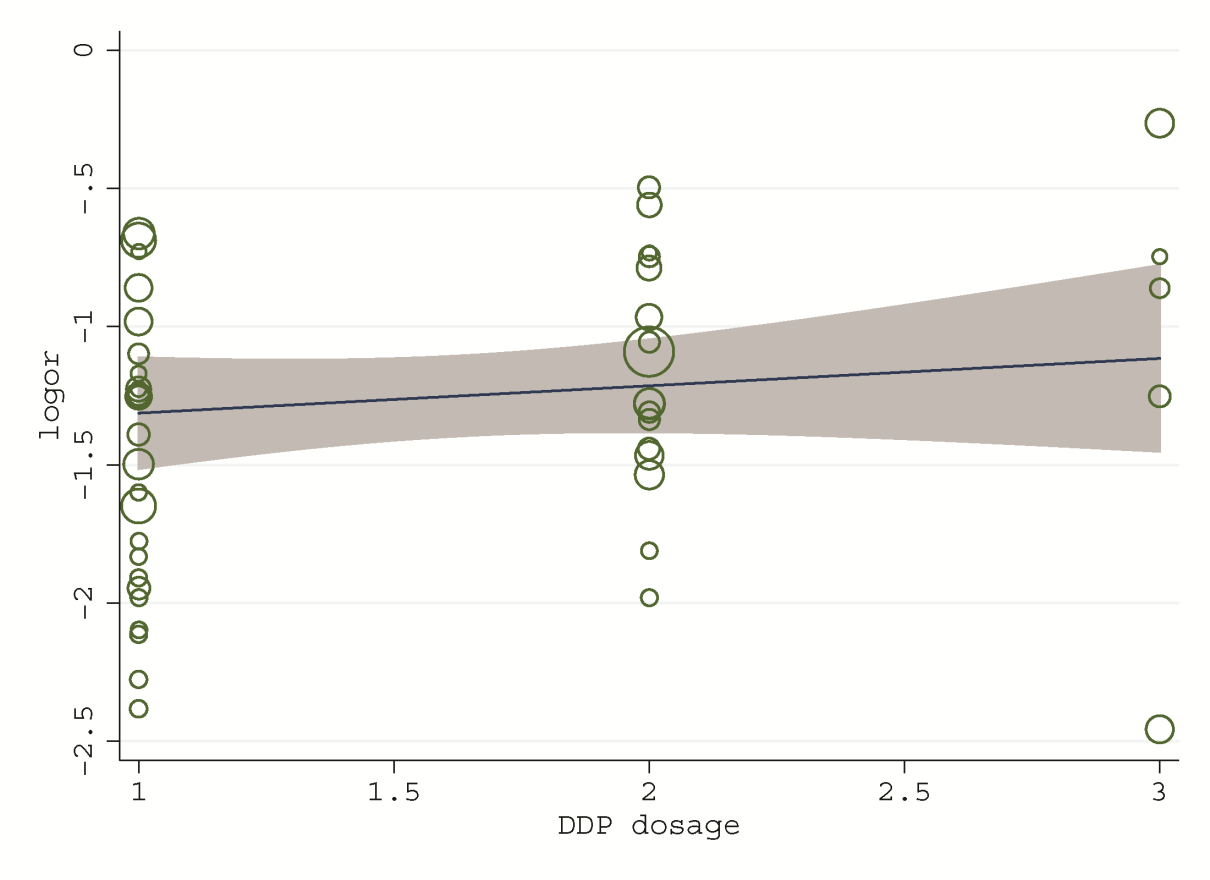


Figure S71. Meta regression of treatment failure via DDP dosage
